# Seagrass genomes reveal ancient polyploidy and adaptations to the marine environment

**DOI:** 10.1038/s41477-023-01608-5

**Published:** 2024-01-26

**Authors:** Xiao Ma, Steffen Vanneste, Jiyang Chang, Luca Ambrosino, Kerrie Barry, Till Bayer, Alexander A. Bobrov, LoriBeth Boston, Justin E Campbell, Hengchi Chen, Maria Luisa Chiusano, Emanuela Dattolo, Jane Grimwood, Guifen He, Jerry Jenkins, Marina Khachaturyan, Lázaro Marín-Guirao, Attila Mesterházy, Danish-Daniel Muhd, Jessica Pazzaglia, Chris Plott, Shanmugam Rajasekar, Stephane Rombauts, Miriam Ruocco, Alison Scott, Min Pau Tan, Jozefien Van de Velde, Bartel Vanholme, Jenell Webber, Li Lian Wong, Mi Yan, Yeong Yik Sung, Polina Novikova, Jeremy Schmutz, Thorsten B.H. Reusch, Gabriele Procaccini, Jeanine L. Olsen, Yves Van de Peer

**Affiliations:** 1 Department of Plant Biotechnology and Bioinformatics, Ghent University, 9052 Ghent, Belgium; 2 VIB Center for Plant Systems Biology, VIB, 9052 Ghent, Belgium; 3 Department Plants and Crops, Faculty of Bioscience Engineering, Ghent University, Coupure Links 653, 9000 Ghent, Belgium; 4 Department of Research Infrastructure for Marine Biological Resources, Stazione Zoologica Anton Dohrn, Villa Comunale, 80121, Naples, Italy; 5 DOE Joint Genome Institute, Lawrence Berkeley National Laboratory, Mail Stop: 91R183, 1 Cyclotron Road, Berkeley, CA 94720, USA; 6 GEOMAR Helmholtz-Zentrum für Ozeanforschung Kiel, Marine Evolutionary Ecology, Wischhofstr. 1-3, 24148 Kiel, Germany; 7 Papanin Institute for Biology of Inland Waters RAS, Borok, Nekouz Distr., Yaroslavl Reg., 152742, Russia; 8 Genome Sequencing Center, Hudson Alpha Institute for Biotechnology, 601 Genome Way, Huntsville, AL 35806, USA; 9 Coastlines and Oceans Division, Institute of Environment, Florida International University-Biscayne Bay Campus, 3000 NE 151^st^ St., Miami, Florida 33181, USA; 10 Department of Agricultural Sciences, University Federico II of Naples, Naples, Italy; 11 Department of Integrative Marine Ecology, Stazione Zoologica Anton Dohrn, Villa Comunale, 80121 Naples, Italy; 12 Institute of General Microbiology, University of Kiel, Kiel, Germany; 13 Seagrass Ecology Group, Oceanographic Center of Murcia, Spanish Institute of Oceanography (IEO-CSIC), Murcia, Spain; 14 Centre for Ecological Research, Wetland Ecology Research Group, Bem tér 18/C, Debrecen, H-4026, Hungary; 15 Institute of Marine Biotechnology, Universiti Malaysia Terengganu, Terengganu, Malaysia; 16 National Biodiversity Future Centre (NBFC), Palermo, Italy; 17 Arizona Genomics Institute, University of Arizona, 1657 E. Helen St, Tucson, AZ 85721, USA; 18 University of Bologna, Department of Biological, Geological and Environmental Sciences, Bologna, Italy; 19 Fano Marine Center, Fano, Italy; 20 Max Planck Institute for Plant Breeding Research (MPIPZ), Department of Chromosome Biology, Carl-von-Linne-Weg 10, 50829 Köln, Germany; 21 Marine Evolutionary Ecology, GEOMAR Helmholtz Centre for Ocean Research Kiel, Düsternbrooker Weg 20, D-24105 Kiel, Germany; 22 Groningen Institute for Evolutionary Life Sciences (GELIFES), University of Groningen, Nijenborgh 7, 9747AG, Groningen, Netherlands; 23 Centre for Microbial Ecology and Genomics, Department of Biochemistry, Genetics and Microbiology, University of Pretoria, Pretoria 0028, South Africa; 24 College of Horticulture, Academy for Advanced Interdisciplinary Studies, Nanjing Agricultural University, Nanjing, China

**Keywords:** Alismatales, convergent evolution, *Cymodocea nodosa*, hexaploidy, *Posidonia oceanica*, *Potamogeton acutifolium*, seagrasses, *Thalassia testudinum*, whole genome duplication (WGD), whole genome triplication (WGT), *Zostera marina*

## Abstract

We present chromosome-level genome assemblies from representative species of each of three independently evolved seagrass lineages, namely *Posidonia oceanica, Cymodocea nodosa, Thalassia testudinum*, and *Zostera marina*. We also include a draft genome of *Potamogeton acutifolius*, belonging to a freshwater sister lineage to Zosteraceae. All seagrass species share an ancient whole genome triplication, while additional whole genome duplications were uncovered for *C. nodosa, Z. marina* and *P. acutifolius*. Comparative analysis of selected gene families suggests that the transition from submerged-freshwater to submerged-marine environments mainly involved fine-tuning of multiple processes, e.g., osmoregulation, salinity, light capture, carbon acquisition and temperature, that all had to happen in parallel, likely explaining why adaptation to a marine lifestyle has been exceedingly rare. Major gene losses related to stomata, volatiles, defense, and lignification, are likely a consequence of the return to the sea rather than the cause of it. These new genomes will accelerate functional studies and solutions — as continuing losses of the ‘savannas of the sea’ are of major concern in times of climate change and loss of biodiversity.

## Introduction

Seagrasses are unique flowering plants, adapted to a fully submerged existence in the highly saline environment of the ocean, where they must root in reducing sediments, endure chronic light limitation, and withstand considerable hydrodynamic forces. In spite of these obstacles, the 80 or so species are among the most widely distributed flowering plants ^1–3^ with recently measured estimates of coverage ranging from 600,000 km^2^ ^4^ to a modeled value of 1,6 million km^2^ ^5,6^. Seagrasses fulfill many critical ecosystem functions and services including carbon sequestration, nutrient cycling, bacterial suppression, and coastal erosion protection ^7–11^. Along with mangroves, saltmarshes, and coral reefs, seagrass meadows are among the most biologically productive ecosystems on Earth. They act as breeding and nursery grounds for a huge variety of organisms including juvenile and adult fish, epiphytic and free-living algae, mollusks, bristle worms, nematodes, and other invertebrates such as scallops, crabs, and shrimp. Their importance for marine megafauna such as sea turtles, dugongs and manatees is unrivalled and their disappearance an important driver of the decline of these marine animals ^12^. Seagrasses also rank amongst the most efficient natural carbon sinks on Earth, sequestering CO_2_ through photosynthesis and storing organic carbon in sediments for millennia ^13^. While occupying only 0.1% of the ocean surface, seagrasses have been estimated to bury 27–44 Tg C_org_ per year globally, accounting for 10-18% of the total C burial in the oceans and being up to 40 times more efficient at capturing organic carbon than land-forests soils ^14^.

Previous work on *Zostera marina* ^15,16^ uncovered several unique gene family losses, as well as metabolic pathway losses and gains, that collectively underly novel structural and physiological traits, along with evidence for ancient polyploidy. Here, we expand on this work and present new chromosome-scale, high-quality reference genomes to understand the specific morphological and physiological adaptations that have enabled their worldwide distribution, except for Antarctica ^1^. These included *Posidonia oceanica* (L.) Delile (Posidoniaceae), *Cymodocea nodosa* (Ucria) Ascherson (Cymodoceaceae), and *Thalassia testudinum* K. D. Koenig (Hydrocharitaceae) to chromosome level assemblies, and a closely related freshwater-submerged alismatid, *Potamogeton acutifolius* Link (Potamogetonaceae), to draft level. Representative seagrass species within each family (Supplementary Figure 1.1) were chosen based on ecological importance, susceptibility to anthropogenic pressure, and availability of an extensive ecological literature. Briefly, *Posidonia oceanica* is the iconic Mediterranean seagrass and the largest in terms of plant size and physical biomass. It is a climax species characterized by extreme longevity and carbon storage capacity. *Thalassia testudium* (turtle grass) is a climax tropical species unique to the greater Caribbean region, with a single sister species endemic to the Indo-Pacific. *Cymodocea nodosa* is restricted mainly to the Mediterranean, Black and Caspian Seas, with an Atlantic extension along the Canary Island archipelago and along the subtropical Atlantic coast of Africa. It is the only temperate species of an otherwise disjunct tropical genus from the Indo-Pacific. The curly pondweed *Potamogeton acutifolius* belongs to the sister family of Zosteraceae and was chosen as its closest submerged freshwater sister taxon. We also included the recently upgraded genome of *Zostera marina* L. ^17^, which is found throughout the northern hemisphere and is arguably the most widespread species on the planet ^18^. To distinguish between adaptations to an aquatic lifestyle, and those unique to the ocean environment, our comparative analysis also included genomes of two recently sequenced emergent freshwater alismatids (which are rooted in underwater substrate, but have leaves and stems extending out of the water), along with the genomes of two distantly related salt-water tolerant mangrove species. In addition, representative transcriptomic data ^16^ of 89 Alismatales species was utilized to gain a more comprehensive view of shared and unique seagrass and freshwater adaptations within the order Alismatales (Supplementary Figure 1.1).

To better understand the extremely rare transition from a freshwater environment to a submerged saline environment, we compared gene family and pathway evolution across species, considering gene loss, as well as gene birth through small and large-scale gene duplication events, and investigated their effect on plant body structure (cell walls, stomata, hypolignification) and also investigated their relationship to physiological adaptations (hypoxia, plant defense, secondary metabolites, light perception, carbon acquisition, heat shock factors and especially salt tolerance mechanisms).

## Results and Discussion

### Genome assemblies and gene annotations

**Figure 1:**
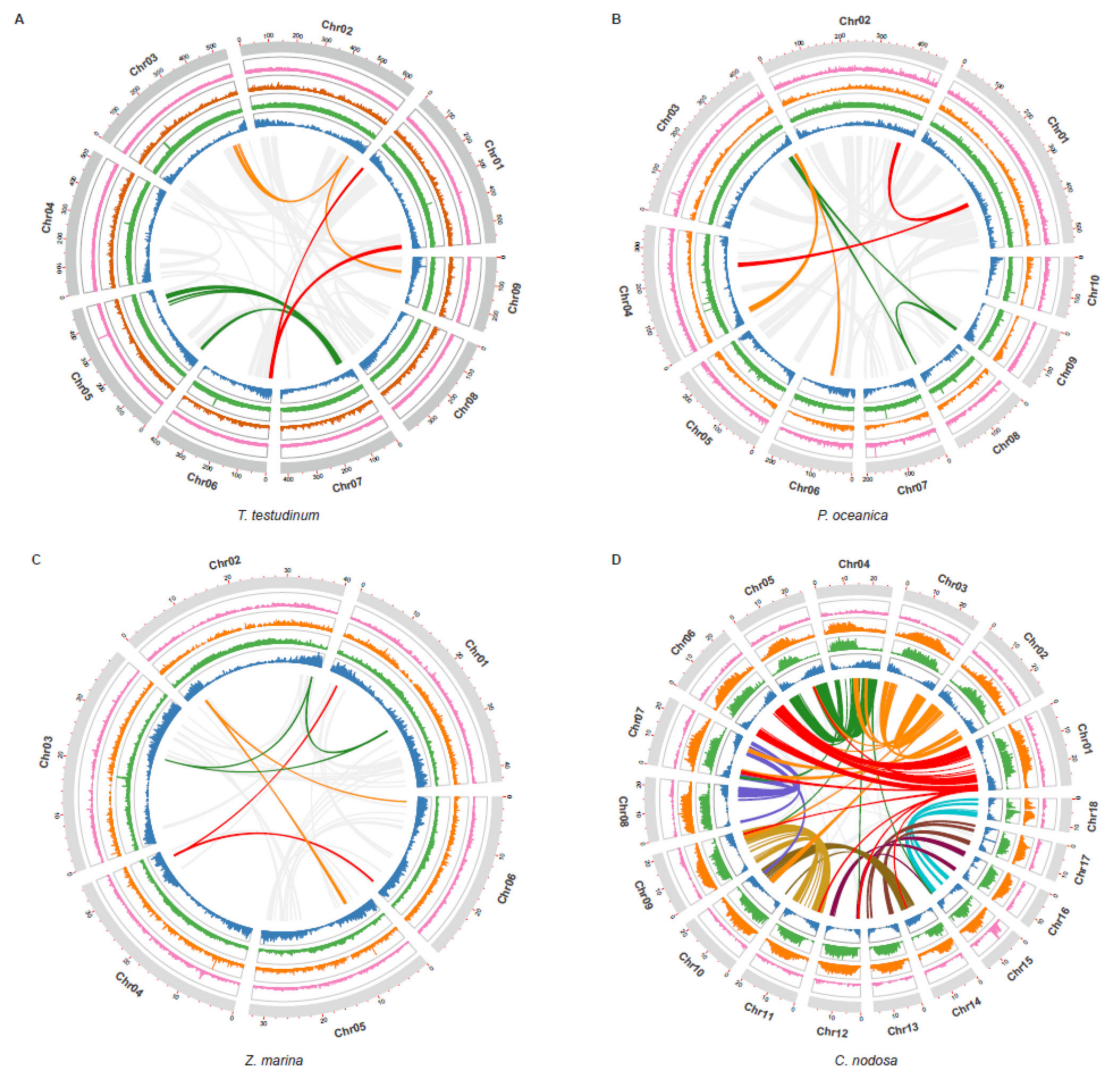
Distribution of the genomic features for the seagrass species *T. testudinum, P. oceanica, Z. marina* and *C. nodosa*. Tracks from the inner to outer side correspond to gene density (blue); LTR/Gypsy density (green); LTR/Copia (orange); DNA transposable elements (pink) and chromosomes (with length in Mb). Curved lines through the center denote synteny between different genomic regions. Grey lines in A, B and C reflect synteny involving the WGD, whereas the three colored lines represent synteny with WGTs. Colored lines in D represent synteny and strong intragenomic conservation and should not be compared with colors in A, B and C (see text for further details). The distribution of the genomic features for the longest scaffolds of *P. acutifolius*, can be found in Supplementary Figure 2.1.2.

We assembled the genomes of *T. testudinum, P. oceanica*, and *C. nodosa* to chromosomal level using a combination of short sequence reads, PacBio HiFi, PacBio long reads, and Hi-C chromosome mapping. The novel seagrass genomes varied in haploid chromosome number from 6 to 18 and were very different in size, while containing approximately the same number of gene models (Supplementary Table 2.1.4). Further details of genome assembly and annotation, based on a combination of *ab initio* prediction, homology searches, RNA-aided evidence, and manual curation can be found in Methods, Supplementary Table 2.1.4., Supplementary Note 2.1, and Supplementary Table 2.1.3. BUSCO scores of >95% demonstrate the high level of completeness in the genomes. The prediction of non-protein coding RNA families (i.e., rRNAs, tRNAs, snoRNAs) for *Z. marina, C. nodosa, P. oceanica, T. testudinum*, and *P. acutifolius* can be found in Supplementary Note 3.1 and Supplementary Table 3.1.). Figure 1 shows the distribution of different genomic features along the reconstructed pseudochromosomes for the different seagrass species. Information on plastid and mitochondrial genomes can be found respectively in Supplementary Note 2.2 and Supplementary Note 2.3.

Information on Nuclear-mitochondria (NUMTs) and nuclear-chloroplast (NUPTs) integrants can be found in Supplementary Note 2.4 and Supplementary Table 2.4.

### Genome Evolution

#### Transposable elements

Transposable elements (TE) comprise more than 85% of the genomes of *T. testudinum* and *P. oceanica*, as compared to only 65% for *C. nodosa* and *Z. marina*, and 40% for *P. acutifolius* (Supplementary Table 4.1). Long terminal-repeat retrotransposons (LTR-REs) are the major class of TEs and account for 72%, 66%, 46% and 42% in *T. testudinum, P. oceanica, C. nodosa* and *Z. marina*, respectively. LTR/Gypsy elements account for 63.18% in *T. testudinum*, 57.8% in *P. oceanica* and 32.11% in *Z. marina*, whereas the proportion of LTR/Copia elements was higher than that of LTR/Gypsy in *C. nodosa* and *P. acutifolius*. Bursts of TEs (especially LTRs) create new genetic variation that may be adaptive under conditions of stress. Over evolutionary time, different TE loads and distributions among species provide clues related to habitat differences and stress resistance ^19,20^. The insertion times of LTRs in the seagrass genomes (Methods) indicates a massive LTR/Gypsy burst around 200 thousand years ago (Kya) in *T. testudinum* (see y-axis), a moderate burst around 400 Kya in *P. oceanica* and *Z. marina*, but not in *C. nodosa*. By contrast, an expansion in Copia-elements happened around 2 Mya in *C. nodosa* but was weaker in *P. oceanica*, and nearly absent in *T. testudinum* and *Z. marina*. The recent TE gypsy burst (200 Kya) and older Copia burst (2 Mya median) coincide with drastic environmental fluctuations during Pleistocene ice ages (Supplementary Figure 4.1) and the timing of the trans-Arctic dispersal of *Z. marina* to the Atlantic from the Pacific ^18^. The Gypsy bursts at 400 and 200 Kya correspond to Marine Isotope Stage MIS12 and MIS6, two heavy glaciations that were followed by rapid warming ^21^.

#### Whole genome duplication, ancient (hexa)polyploidy and dating

Next, we revisited the established whole genome duplication (WGD) in *Z. marina* ^15^ and investigated whether evidence for ancient polyploidy could be found in the other seagrasses, which are all behaving as functional diploids ^22^. To this end, we used inferred age distributions of synonymous substitution rate (K_S_) for paralogs retained in collinear regions (anchor pairs), along with gene-tree/species-tree reconciliation methods (see Methods, Supplementary Note 4.2.1 and Supplementary Note 4.2.2). First, K_S_ distributions of all seagrass species showed peaks indicative of ancient WGDs (Supplementary Figure 4.2.1)^16^. This was supported by intra- and intergenomic collinearity analysis (see Supplementary Note 4.2.1). Comparison of *P. oceanica* and *T. testudinum* with a reconstructed ancestral monocot karyotype (AMK ^23^) shows a clear 3:1 synteny relationship, while a comparison of *Z. marina* with the AMK exhibits a 1:6 synteny relationship (Supplementary Figure 4.2.2). *Cymodocea nodosa* was also found to show a 6:1 relationship compared to the AMK, while showing a 2:1 relationship with its sister species *P. oceanica* (Supplementary Figure 4.2.3), providing strong support for an additional WGD in *C. nodosa* after diverging from the *P. oceanica* lineage. Likewise, the freshwater species *P. acutifolius* was found to show a collinear relationship of 6:1 with the AMK and a 2:1 relationship with *P. oceanica*, and a 2:2 relationship with *C. nodosa*, while the colinearity relationship with its sister species *Z. marina* was more obscure (Supplementary Figure 4.2.4). However, these findings provide evidence that also *P. acutifolius* experienced an additional WGD event after its divergence with *P. oceanica* and *C. nodosa*. Of note, the overall 1:3 or 1:6 synteny relationships with the AMK suggested a hexaploid rather than a tetraploid ancestry for seagrasses and relatives.

**Figure 2:**
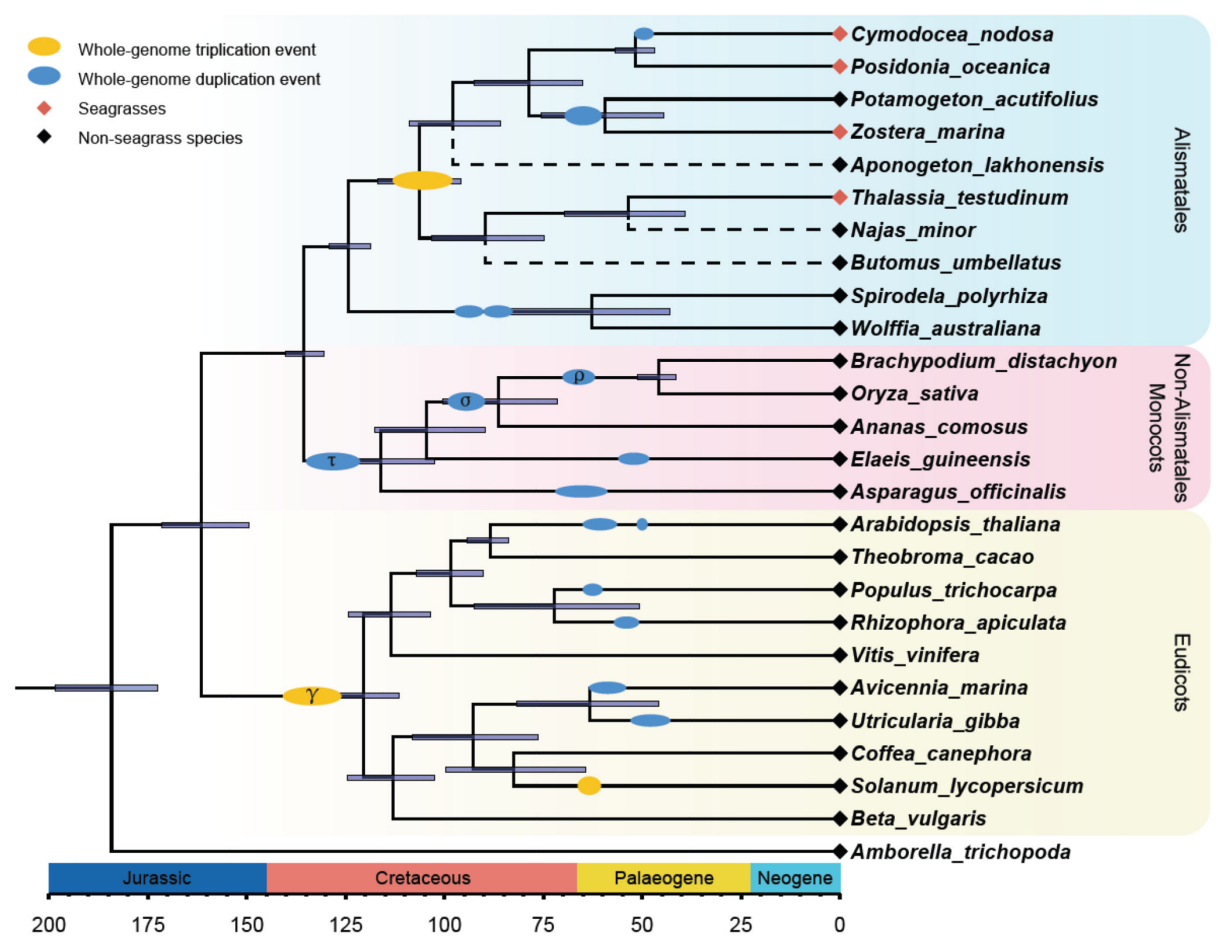
Time-calibrated phylogeny and WGT/WGD events across flowering plants that have chromosome-level genome assemblies. The tree was inferred from 146 single-copy genes and show WGDs and WGTs based on inferences from the current study and previous analyses (Supplementary Table 4.2 and Supplementary Figure 4.2.8). For a more comprehensive tree showing the phylogenetic position of seagrasses within Alismatales, see Supplementary Figure 1.1. The dashed lines represent additional freshwater Alismatales species (phylogenetic position inferred using transcriptome data), mainly added for illustrative purposes to show non-monophyly of seagrass species. All branches have bootstrap support >98%. See text and Methods for details.

Second, based on a K_S_ analysis using ksrates ^24^, we were able to confirm that this paleohexaploidy is shared by *P. oceanica, C. nodosa, Z. marina*, and *P. acutifolius*, while the analysis was inconclusive for *T. testudinum* (Supplementary Figure 4.2.5). To resolve this issue, we applied a gene-tree/species-tree reconciliation approach using WHALE ^25^, which confirmed that the ancient whole genome triplication (WGT) event is shared by all seagrasses, and *P. acutifolius*. WHALE also supported the younger WGD in *Z. marina* is shared with *P. acutifolius* (Supplementary Note 4.2.2 and Supplementary Figure 4.2.6). Phylogenomic dating of the WGT (see Methods and Supplementary Note 4.2.3) further shows that most gene duplicates are reconciled on the branch leading to the most recent common ancestor (MRCA) of Potamogetonaceae, Zosteraceae, Posidoniaceae, Cymodoceaceae and Hydrocharitaceae, at approximately 86.96 (89.89 - 79.81) Mya (Figure 2 and Supplementary Figure 4.2.7a). Recently, Chen et al. ^16^ also reported a WGD shared by all core Alismatales (Supplementary Figure 1.1). However, these authors suggested a WGD rather than a WGT, which can be attributed to the lack of structural data, since their study was based solely on transcriptome data. Independent absolute dating of the shared WGD for *P. acutifolius* and *Z. marina* confirmed an earlier obtained date for the *Zostera* WGD of approximately 65 Mya (Supplementary Figure 4.2.7c-f), coinciding with the K/Pg boundary ^15^, which was also used to date a recent within-species phylogeographic study for *Z. marina* ^18^.

### Adaptation to the Marine Environment

**Figure 3:**
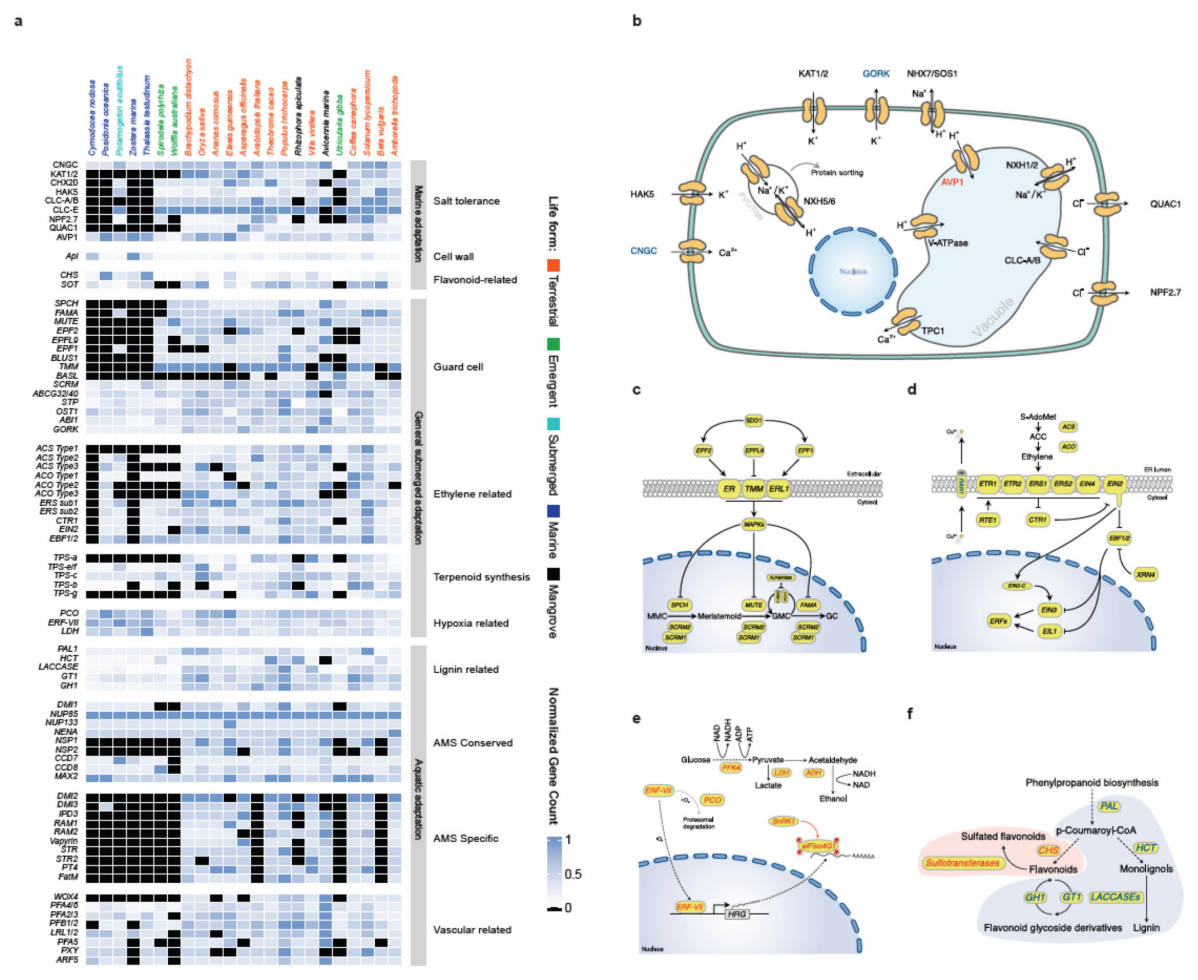
The loss, contraction, and expansion of gene families involved in the adaption to a marine environment. **a)** The normalized gene copy numbers for 4 seagrasses and 19 representative non-seagrass species. The normalization on the family dataset divides the gene count number of each species by the largest gene copy number within that family. The species order on the top of the heatmap is the same as that in Figure 1. The colors correspond to the different life-forms. The orange ones are terrestrial species; the green ones are emergent species (floating-leaved); the light blue ones are submerged species; the navy-blue ones are marine species (seagrasses) and the black ones are mangroves **b)** Salt stress signaling implies different ion channels. *HAK5* encodes HIGH-AFFINITY POTASSIUM TRANSPORTER 5; CNGC, CYCLIC NUCLEOTIDE GATE CATION CHANNELs; *AVP1* encodes Vacuolar H^+^-PPases **c)** Stomata differentiation from meristemoid mother cells (MMC) to guard mother cell (GMC), to guard cells. **d)** Ethylene synthesis and signaling. **e)** The hypoxia-responsive signaling in which the direct (*ERF-VII*) and indirect responsive (*SnRK1*) pathways are expanded. The rate-limiting enzyme (encoded by *PFK4*) in the glycolysis pathway, along with Lactate dehydrogenase (encoded by *LDH*), a rate-limiting enzyme in fermentation, are also expanded. **F)** Simplified schematic of the lignin and flavonoid biosynthesis pathways. Only steps that have significantly changed are shown. *PAL* encodes phenylalanine ammonialyase, which is the gateway enzyme of the general phenylpropanoid pathway; *CHS* encodes chalcone synthase, which is the first enzyme of flavonoid biosynthesis that directs the metabolic flux to flavonoid biosynthesis; *GT1* encode flavonoid glycosyltransferases, which catalyze the final step of flavonoid biosynthesis to generate various flavonoid glycoside derivatives; *GH1* encode flavonoid beta-glucosidase & myrosinase, which are responsible for the recycling of carbohydrate-based flavonoids; *HCT* encode Hydroxycinnamoyl‐CoA shikimate/quinate hydroxycinnamoyl transferase, channels phenylpropanoids via the “esters” pathway to monolignols”; *LACCASEs* encode the final enzymes in the pathway that oxidize monolignols to facilitate their polymerization into lignin. Panels d) e) f) genes in red are expanded; blue means contracted; The dashed line in the pathway means multiple metabolic steps.

All three seagrass lineages characterized in this study share many specific morphological and physiological adaptations to their specific environment. Historically, a number of features were proposed as prerequisites for marine angiosperm life, such as tolerance to submergence, tolerance to salinity, hydrophilous pollination, and a capacity for vegetative anchorage ^26,27^. Previous studies have already reported genes potentially linked to the adaptation to the marine environment ^15^, while a recent study that conducted a broad transcriptome-based sampling of Alismatales uncovered some patterns of gene loss and gain also likely associated with aquatic and/or marine adaptation ^16^. Discrimination between aquatic (i.e., freshwater) and marine adaptations is not necessarily easy. To achieve greater insights into both adaptations, we used a common set of species for which full genome information is available (four seagrasses, three freshwater alismatids, and 16 other angiosperms, Figure 2 and Supplementary Note 4.3). We also utilized the extensive transcriptome dataset of Chen et al. (^16^) and broadly assessed commonalities and differences in gains and losses across gene families (further referred to as orthogroups, see Methods and Extended Data Table 1-10). The most important findings on adaptation to both aquatic-submerged, and marine conditions are summarized in Figure 3 (and Supplementary Figure 5.1).

#### Use it or lose it - convergence and specificity of gene losses

Under water, stomata are not required and may even be harmful for a submerged lifestyle because of the intrusion of water. Hence, seagrasses, and to a limited extent also freshwater alismatids, e.g., *P. acutifolius*, have reduced the number of genes involved in their development. Specifically, out of 30 orthogroups containing guard cell toolkit genes ^28^, eleven have been convergently and completely lost in seagrasses, while six others were significantly contracted compared to non-seagrass genomes (Figure 3a and Extended Data Table 1). Lost gene families include positive (SMF transcription factors), negative (EPIDERMAL PATTERNING FACTOR1 AND 2 (encoded by *EPF1, EPF2*), and TOO MANY MOUTHS (encoded by *TMM*)) regulators of stomatal development, as well as stomatal function (encoded by *BLUS1, KAT1/2* and *CHX20*) (Figures 3a and 3c). Gene losses and contractions in the guard cell toolkit are also seen in the submerged freshwater alismatid *P. acutifolius* studied here, and to a less extreme degree in the floating alismatid *S. polyrhiza* (Figure 3a and Extended Data Table 1).

The aqueous habitat of seagrasses is also not conducive to emitting volatile substances as signals. Accordingly, we observed a convergent loss of orthogroups associated with volatile metabolites and signals. This includes the biosynthesis of triterpenes, and the volatile systemic acquired resistance signal, methyl salicylate ^29^ (Extended Data Table 2). Probably a more dramatic gene loss relates to ethylene biosynthesis and signaling (Extended Data Table 2). Two species, *C. nodosa* and *Z. marina*, do not contain *ACS* or *ACO* genes and hence, are not expected to produce ethylene or its precursor 1-aminocyclopropane 1-carboxylic acid (ACC). Moreover, they seem to have lost the ability to respond to ethylene, as indicated by a severe contraction of the early ethylene signal transduction components (Figure 3a and 3d) ^15,16,30^. In contrast, the downstream ethylene transcription factors (encoded by *EIN3/EIL1/2*) have been retained in all seagrasses, suggesting they can still exert ethyleneindependent functions. Remarkably, and unlike *C. nodosa* and *Z. marina, T. testudinum* and *P. oceanica*, as well as freshwater submerged species, retained some components for functional ethylene biosynthesis and signaling, as was also reported by Chen et al. ^16^. As diffusion of ethylene into water is extremely slow compared to diffusion in the air, ethylene rapidly accumulates in submerged organs. Such accumulation typically serves as a signal for submergence, and activating adaptive responses, such as formation of aerenchyma, adventitious rooting, shoot elongation, quiescence and priming the metabolism for efficient low-oxygen responses ^31,32^. However, while the accumulation of ethylene can be considered beneficial for the flooding tolerance of land plants, high levels and prolonged exposure to ethylene can have detrimental effects, such as stunted growth, senescence and abscission of leaves and flowers, root growth inhibition, and increased stress sensitivity ^33^. One possible mechanism that may prevent the accumulation of deleterious levels of ethylene, and thus explain its retention in *T. testudinum* and *P. oceanica*, is via epiphytic and endophytic bacteria that express ACC deaminases. This hypothesis is supported by the presence of multiple ACC deaminases in the metagenome of *P. oceanica* sediments ^34^, but needs further study.

Seagrasses increase their morphological flexibility to withstand hydrodynamic wave and current forces by a reduction in vascular tissues, the main site of lignification ^35^, consistent with the absence of vascular proliferation factor encoded by *WOX4*, and a contraction of the number of pericycle cell identity transcription factors (Figure 3a and Extended Data Table 3). This finding seems a more general adaption to aquatic lifestyles, as also suggested by analysis of the transcriptomes of different Alismatales (Supplementary Figure 5.1, this study, and ref (^16^)). The most severe reduction of the vascular bundle is seen in *Z. marina* which even lacks a pericycle^36^, a finding that correlates with the loss and divergence of the vascular proliferation regulators encoded by *PXY* and *MONOPTEROS/ARF5* (Figure 3a and Extended Data Table 3). Notably, the lack of *MONOPTEROS/ARF5* in *Z. marina* is further reflected in its inability to form an embryonic primary root ^37^. The general cellular hypolignification in seagrasses is reflected in the reduction in the number of *LACCASE*s encoding the final enzymes in the lignin pathway, which oxidize monolignols to facilitate their polymerization into lignin ^38,39^ (Figures 3a, 3e and Extended Data Table 4). The reduced need for the monolignol production is matched by a reduction of respectively PHENYLALANINE AMMONIA LYASE (encoded by *PAL*), and HYDROXYCINNAMOYL-COA SHIKIMATE/QUINATE HYDROXYCINNAMOYL TRANSFERASE (encoded by *HCT*) genes, which constitute entrance points into phenylpropanoid biosynthesis ^40^ (Figures 3a, 3f and Extended Data Table 4). Gene family contractions in lignin biosynthesis are also observed for the submerged freshwater species *P. acutifolius* and the freshwater floating species *S. polyrhiza* (Figure 3a).

Arbuscular mycorrhizal symbiosis (AMS) were and are critical for plant terretrialization ^41,42^ and are found in salt marsh plants, mangrove forests, and freshwater ecosystems ^43–45^. There is currently no evidence for any seagrass species to form mycorrhizal associations ^46^, which is reflected in the absence (secondary loss) of AMS-specific genes, with the sole exception of DMI3 in *P. oceanica* (Figure 3a). Gene loss of AMS-specific genes is also seen in freshwater submerged and floating species (Figure 3a). We also investigated so-called AMS-conserved genes, which have non-symbiotic roles ^47^ and discovered that seagrasses and *P. acutifolius* consistently retained a specific set of these conserved genes (DMI1, NUP85, NUP133, NENA, CCD7, CCD8 and MAX2) (Figure 3a). The absence of NSP1 and NSP2 is not unique to seagrasses but seems to be rather a common adaptation observed in aquatic environments (Supplementary Figure 5.1) and Proteales species ^48^.

The pathogen landscape of the marine environment is associated with a different composition of plant resistance (R-genes) genes. In the seagrasses, there are fewer genes containing nucleotide-binding leucine-rich repeat receptors (NLRs) as compared to most other plants (Extended Data Table 5, Supplementary Note 5.2, Supplementary Table 5.2 and Supplementary Figure 5.2.1). As in many monocots, NLRs with a Toll/interleukin-1 receptor/resistance protein (TIR) domain are also completely absent in all seagrass lineages, as well as a few other NLR genes from the leucine rich repeat (LRR) domain. It is currently unclear what selective pressure was responsible for the unique R-gene composition of the seagrasses. Lower counts of disease resistance genes have also been observed for other aquatic plants ^49^.

Temperature fluctuations are much slower and show a lower amplitude in the marine compared to terrestrial environment ^50^. Accordingly, we observed a reduction in the number of plant heat shock transcription factors (*HSF*s) that are involved in the rapid activation of stress-responsive genes upon temperature changes, and which have been linked to the evolutionary adaptation of plants to the terrestrial environment ^50^. Seagrasses contain only about half the number of *HSF*s as compared with terrestrial plants (Extended Data Table 5, Supplementary Note 5.3 and Supplementary Table 5.3). Notably, only seagrasses belonging to the tropical genera retained some of the key heat stress-related *HSF*s from WGD and WGT events (Extended Data Table 5), which is consistent with their warmer native environment and higher heat stress tolerance compared to temperate seagrasses (*P. oceanica* and *Z. marina*).

### Multi-level “tweaking” to adapt to the marine environment

#### Protective flavonoids and phenolics

Most seagrasses, except *C. nodosa*, seem to have greatly expanded the number of CHALCONE SYNTHASEs, which channel *p*-coumaroyl-CoA into flavonoid biosynthesis at the expense of monolignol biosynthesis (Figure 3a, 3f, and Extended Data Table 6). Flavonoids provide protection against UV and fungi, while enhancing recruitment of N-fixing bacteria ^34,51,52^. Flavonoids and other phenolics in seagrasses can be sulphated by the activity of cytosolic sulphotransferases to increase their water solubility and bioactivity in the marine environment ^53,54^. For example, the sulphated monolignol, zosteric acid (*O*-sulfonated *p*-coumaric acid) is an antifouling agent that prevents biofilm formation at the leaf surface ^55^. Cytosolic sulphotransferases are expanded in seagrasses, but significantly contracted in *Potamogeton*. However, flavonoid glycosyltransferases and flavonoid beta-glucosidases are contracted in both (Figure 3a, 3f, and Extended Data Table 6). Jointly, these data illustrate how rerouting precursors of the lignin biosynthesis pathway likely facilitated two traits, i.e., reduced rigidity, which appears to be a general aquatic adaptation, and sulphated protection, which contributes to the evolution of the marine lifestyle of seagrasses ^34,54^. In the case of *P. oceanica*, secreted phenolic compounds, together with anoxia, both inhibit microbial consumption of sucrose from root exudates ^34^.

### Diverse mechanisms of cellular salt tolerance

Salt tolerance in flowering plants is a complex trait that involves multiple cellular processes ^56^. In the extreme case of invasion of highly-saline, marine environments, one might assume wholesale changes in salt tolerance mechanisms and/or the evolution of specialized features, such as salt glands in mangrove species. To date, no obvious specialized structures involved in salt tolerance have been identified in seagrasses. Instead, it seems that canonical salt tolerance mechanisms have been fine-tuned or “tweaked” towards higher efficiency on multiple levels. A major challenge associated with the marine environment is to prevent the accumulation of noxious levels of Na^+^ and Cl^-^, while allowing the efficient uptake of the essential ion K^+^. Angiosperms employ secondary Na^+^ transport mechanisms based on Na^+^/H^+^ antiporters fueled by a strong electrochemical H^+^ gradient. Surprisingly, no notable gene gains or losses were observed among the putative sodium transporting NHXs (*NHX1* and *SOS1/NHX7*), except for *C. nodosa*, which contains a few extra copies of *NHX1* and *SOS1* orthologs (Extended Data Table 7). Instead of an increased number of genes, we observed similar amino-acid substitutions in regulatory domains of *SOS1* orthologs in all four species (Supplementary Figure 5.4.1), indicating the possibility of altered regulation of *SOS1/NHX7* in these species, a notion that is also supported by the loss of *SOS3*, a key regulator of *SOS1* activity in *C. nodosa* (Extended Data Table 7). The electrochemical H^+^ gradients that fuel Na^+^ transport is established via H^+^ ATPases (encoded by *AHA*), V-ATPases and vacuolar H^+^-PPases (encoded by *AVP1*). Of these genes, only the *AVP1* genes were obviously expanded in all the seagrasses, containing almost twice the number of *AVP1* genes found on average in other angiosperms (Figure 3a and 3b). Interestingly, the expansion of *AVP1*-like genes can, at least partly, be linked to the ancient WGT followed by their specific retention, suggesting that these additional AVP copies were co-opted for adaptation to a marine lifestyle (Supplementary Figure 5.5.2). Indeed, overexpression of such PPases has been shown to improve salt tolerance in several angiosperms (e.g., *Arabidopsis*, poplar, sugar cane) ^57–59^, by enhancing Na^+^ sequestration in the vacuole ^60^. Analysis of the K^+^-channel repertoire in seagrasses reveals the loss of Shaker-type K^+^ channels (Supplementary Figure 5.4.2) ^61^, and a greatly reduced number of CYCLIC NUCLEOTIDE GATE CATION CHANNELs (Figure 3a, 3b and Extended Data Table 7). Moreover, the constant high K+ concentrations in seawater (9.7mM) renders high-affinity K+ transport systems superfluous, explaining the absence of *AtHAK5* in all seagrass genomes (Figure 3a and 3b). Also, the Cl^-^ transporter repertoire is reduced in seagrasses (Figure 3a and 3b), and seagrasses lack orthologs for NPF2.4 and ALMT12/QUAC1, CLC-A, B and CLC-E, likely reflecting their adaptation to a marine lifestyle (Figure 3a and 3b).

Maintaining the elasticity of the cell wall is another critical component of salt tolerance. The elasticity and structural strength of the cell wall are mainly dictated by components such as cellulose and pectins that cross-link the cellulose microfibrils. The bivalent cation Ca^2+^ stiffens the cell wall by establishing electrostatic bond between pectin strands. The excess of monovalent Na+ in seawater may displace the divalent calcium and hinder dimerization of homogalacturonan chains that are present in canonical pectin ^62^. In addition to the canonical pectin polysaccharides, seagrasses deposit apiogalacturonan in their cell walls ^63^. The borate-bridges that cross-link apiogalacturonan chains are less sensitive to sodium displacement, providing an advantage to plants grown under high salt condition ^64^. One of the few known key enzymes in the synthesis of apiogalaturonan is UDP-D-apiose/UDP-D-xylose synthase (encoded by *Api*), which converts UDP-D-glucuronate into UDP-D-apiose ^65^. Its expansion in seagrasses (in particular in *Zostera* and *Cymodocea*) is reflected in the cell-wall composition of seagrasses and therefore likely contributes to salt tolerance (Figure 3a). In addition, the apiogalacturonan could provide a way to incorporate boron into the cell wall, and protect seagrasses against its toxic effects.

Compared to terrestrial lineages, no major changes were observed for cellulose and hemi-cellulose biosynthesis (Extended Data Table 7). Notably, most of the salt related evolutionary changes in seagrasses are not reflected in the genomes of mangrove species (*Avicinea marina* and *Rhizophora apiculate*), which is consistent with the independent evolution of salt tolerance in mangrove species ^66,67^.

#### Coping with hypoxic sediments

The solubility of oxygen in seawater is limited (typically around 10 mL O_2_ L^-1^), while the sediments in which seagrasses grow are oxygen-free and reducing below a sediment depth of a few mm. This increases the O_2_ demand/draw-down by extensive belowground root-rhizome tissues that often comprise >50% of total plant biomass. Consistent with the increased risk of hypoxia, all seagrasses have expanded their repertoire of Plant Cysteine Oxidases (encoded by PCOs) and group VII Ethylene Responsive (*ERF-VII*s) genes, for direct sensing and transcriptional adjustment to hypoxia (Figure 3a, 3e and Extended Data Table 8). As expected, most *ERF-VII*s had higher expression in rhizomes and roots as compared to leaves (Supplementary Figure 5.5.1). Also, *P. acutifolius* contains an expanded hypoxia response machinery, reflecting its adaptation to submergence (Figure 3a). This is also supported by the transcriptome data of other Alismatales (Supplementary Figure 5.1) ^16^. Again, many, if not most, *ERF-VII* members reside within syntenic blocks retained from the WGT event in seagrasses, especially for *P. oceanica* and *T. testudinum* (Supplementary Figure 5.5.2). Such increases in the number of genes through whole genome duplication is also true for multiple hypoxia-related genes. Some examples are: (1) the *PFK4* gene family, which encodes the rate-limiting enzyme in the glycolysis pathway (including enolases), expanded in both seagrasses and *P. acutifolius*, and derived from the WGT event (Supplementary Figure 5.5.2); (2) Lactate dehydrogenase, a rate-limiting enzyme in lactate fermentation, that is also expanded in seagrasses (Figure 3a and Extended Data Table 8) and has been shown to provide higher waterlogging tolerance in *Arabidopsis* upon overexpression ^68^; and (3) genes encoding the energy-sensing sucrose nonfermenting kinase *SnRK1* ^69^ and *eIFiso4G1* (the dominant regulator in translational regulation by *SnRK1* under hypoxia ^70^) (Extended Data Table 8) are increased as a result of the WGT (Supplementary Figure 5.5.2). In conclusion, we speculate that the increase and specific retention of many hypoxia responsive genes, subsequent to the WGT (dated at ~86 Mya), might have coincided with the Cenomanian-Turonian anoxic event (~91± 8.6 Mya, ^71,72^); if true, this low oxygen period may have helped to select for hypoxia tolerance in submerged species. In *C. nodosa* and *P. acutifolius*, additional recent lineage specific WGDs and tandem duplications may have also contributed to further expansion of the hypoxia responsive genes as a possible adaptation to submergence.

#### Light perception and photosynthetic carbon acquisition

Seagrass growth and zonation are constrained by light availability, as ocean waters rapidly attenuate photosynthetic active radiation with depth and modify its spectral quality, enriching blue while reducing red wavelengths ^73^. Most seagrass species grow in shallow water and even in the clearest waters, only a few species reach depths of 40 m or more. Dissolved inorganic carbon (DIC) is mainly available as bicarbonate (HCO_3_^−^) in seawater (nearly 90% DIC at normal pH) that needs to be exploited via special acquisition systems, as it cannot diffuse passively across the cell plasma membrane ^74^. The availability of dissolved CO_2_ for photosynthesis is instead limited to ~1% of the DIC pool, hence submerged plants and algae evolved CO_2_-concentration and convergent evolution of HCO_3_- to CO_2_ mechanisms (CCMs) to overcome this low availability. A recent report identified an evolutionary adaptation of RuBisCO kinetics across submerged angiosperms from marine, brackish-water and freshwater environments that correlates with the development and effectiveness of CCMs ^75^.

The analysis of genes related to inorganic carbon (Ci) acquisition revealed a slight increase in extracellular *α-CA* (encoding Carbonic Anhydrase α-type) copy number across the studied species (Supplementary Note 5.6.1). In *P. oceanica* and *P. acutifolius*, extra genes again have been specifically retained following the WGT event, although some copies also evolved local tandem duplications. *α-CA* OG0013954 was found to be specific to seagrasses (except for *T. testudinum*) and *P. acutifolius* (Extended Data Table 9 and Supplementary Table 5.6), and most of the corresponding genes are highly expressed in leaves (Supplementary Figure 5.6.1). This supports their involvement in Ci acquisition and possibly CCMs, as the presence of external CAs catalyzing the apoplastic dehydration of HCO_3_^−^ to the RuBisCO substrate CO_2_, together with a higher activity of the extrusion proton pumps ^76^, likely evolved to alleviate dissolved inorganic carbon limitation in most seagrass species ^77^.

Our findings of a retention of 15 C4-related genes after WGT or WGD events (of which two encode PEPC) support the hypothesis that *C. nodosa* could be a C4 species ^78^, similar to what has been observed in *P. acutifolius* (Extended Data Table 9). Notably, none of the studied seagrass species possesses the Serine-residue characteristic of C4 Phosphoenolpyruvate carboxylase (PEPC), thus likely ruling out that a terrestrial-like C4-based (biochemical) CCM system is operating in seagrasses. This would suggest the presence of some kind of C3-C4 intermediate metabolism. Alternatively, homologs to C4 genes could have a role in the resistance of seagrasses to a variety of abiotic stresses, including salt stress ^79^.

Consistent with an augmented need for light capture, seagrasses show an expansion of LHCB (encoding light-harvesting complex B) as compared to freshwater plants that occur close to the water surface (Supplementary Figure 5.6.2 and Supplementary Note 5.6.2). Only *C. nodosa* had a number of LHCB genes comparable to the freshwater *P. acutifolius* and *Spirodela* spp. Other components of the photosynthetic machinery, including Photosystems I and II, are similar in gene number to other species, either freshwater or terrestrial (Supplementary Figure 5.6.2). Seagrasses have conserved the full repertoire of orthologous genes encoding photosensory proteins and components of the light signaling systems (Supplementary Figure 5.6.4 and Supplementary Note 5.6.3) that evolved in the green lineages during the different stages of plant terrestrialization ^80^.

Species-specific adaptation to UV tolerance and downstream regulation, and its relation to light habitat features during the invasion of the marine environment, appear to have differed among seagrass lineages (Supplementary Note 5.6.3). Those living at lower latitudes with intense UV-B radiation throughout the year (*T. testudinum* and *C. nodosa*) have kept the typical *UVR8* of land plants along with their main regulatory proteins (encoded by *RUP1,2*). In contrast, *Z. marina*, as a higher latitude species, has lost the genes for both photoreceptors and their main negative regulatory proteins (Supplementary Figure 5.6.4), consistent with its lower exposure to UV-B radiation. In *P. oceanica*, a species restricted to the Mediterranean, the orthologous gene for *UVR8* lacks the sequence region C27 engaged in the regulation of UVR8 reversion state from the activated to the inactivated state. The species-specific adaptation in the UV-signaling and its negative feedback regulation (Supplementary Figure 5.6.5), further reinforce the idea that ‘tweaking’ and not massive change of key traits and their regulatory mechanisms facilitated the invasion of the marine environment.

Perception of surrounding light cues is also critical for the entrainment of the circadian clock system which in turn is essential for regulation of basic physiology and the life cycle, e.g., daily water and carbon availability, and hormone signaling pathways ^81^. All seagrass species, except *T. testudinum* have lost the *TIMING OF CAB1* (encoded by *TOC1*) gene (Supplementary Figure 5.6.4). The general reduction of clock genes in aquatic species suggests that the “absence of drought”, has led to a reduction of the regulatory daily-timing constraints for some metabolic and developmental plant processes. We find it interesting that all seagrasses have retained some genes related to the phytochromes light-signaling pathway. These include *PIF*s and *LAF1* (Supplementary Figure 5.6.4) following WGT and WGD events, as well as genes related to the circadian clock and photoperiodism such as GI and ZTL (Supplementary Figure 5.6.4).

#### No Apical Meristem (NAC) Transcription Factors (TF)

NAC transcription factors (TF) are among the largest plant-specific-transcription factor (TF) families involved in signaling crosstalk events. They mediate development and aging programs and environmental stress signals. While a comparable number of sequences are found in seagrasses as compared to land plants, freshwater and mongrove species, specific orthogroups were restricted to seagrasses. One of them is annotated as Transcription factor JUNGBRUNNEN 1 (encoded by *JUB1*), a central longevity regulator that is also involved in (salt) stress tolerance. A detailed screening of sequences annotated as JUB1 across other plant genomes reveals sequence similarities and functional reorganizations among JUB1 found in *C. nodosa* and *P. oceanica*. Besides the sequence similarity between the two species, only *C. nodosa* sequences are expressed (Supplementary Note 5.7 and Supplementary Figure 5.7). This difference in functional regulation could potentially be linked to the different ecological tolerance of the two species to environmental factors. Although the two species can coexist, *C. nodosa* can colonize enclosed and shallow environments, which have higher fluctuation range and speed of salinity, light and temperature.

#### Nitrogen Metabolism

Key genes linked to nitrogen uptake/transport and assimilation have been retained in all seagrasses examined, although nitrate transporters (encoded by NRTs) are strongly contracted (Extended Data Table 10 and Supplementary Note 5.8). This implies that seagrasses may have evolved alternative mechanisms for nitrogen uptake and utilization. Although our results are not particularly revealing in this regard, recent work on seagrass microbiomes has shown that nitrogen acquisition involves nitrogen-fixing bacteria in the roots ^82^ and that epiphytic micro-organisms on the leaves mineralize amino acids via their heterotrophic metabolism ^83^. Gaining a more mechanistic understanding of the plant role in these interactions, is now possible for future investigations, given these new genomes.

#### Flower Development

**Figure 4:**
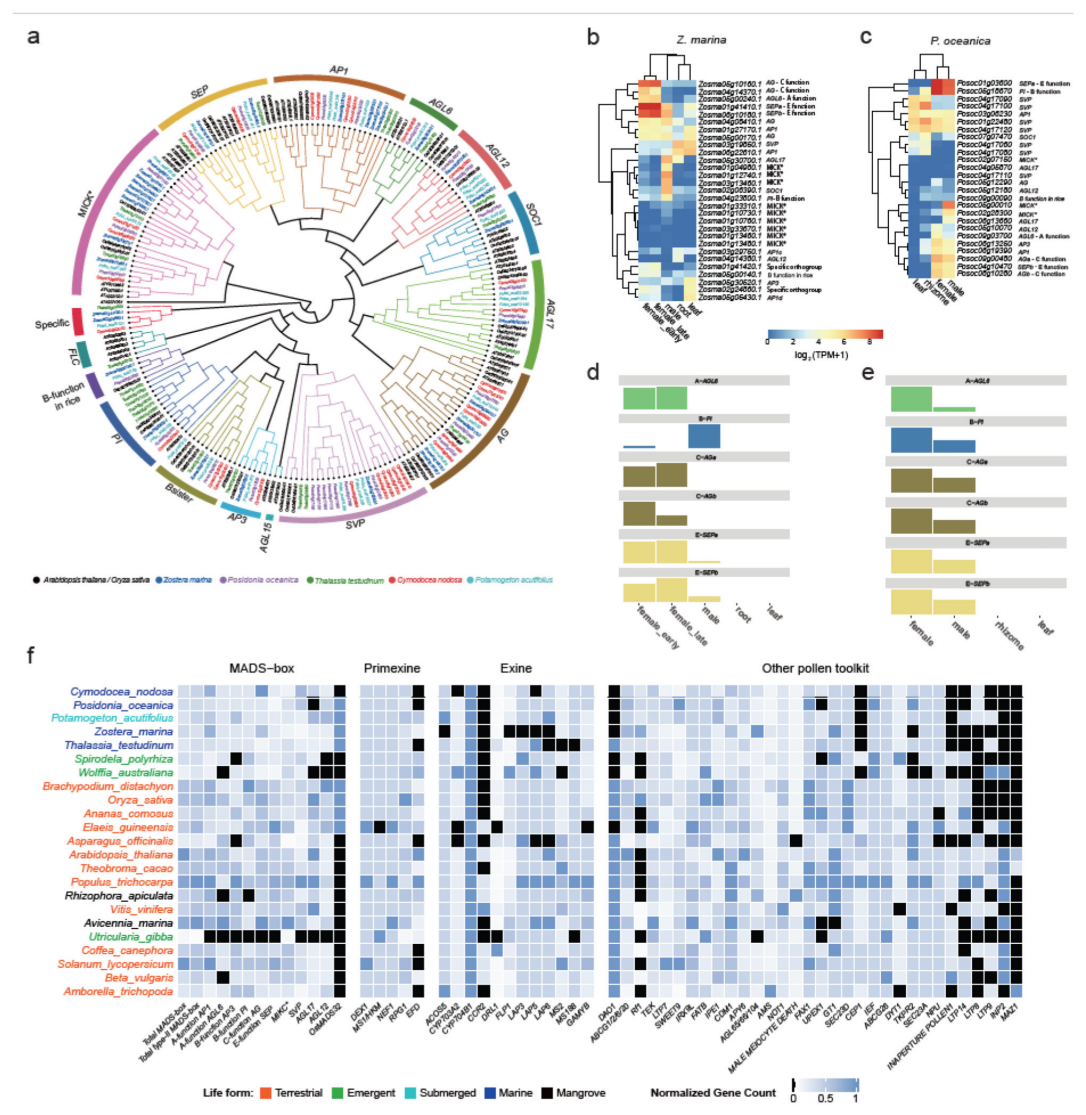
Flower development (like MADS-box genes) and pollen toolkit genes. a) Phylogenetic tree of type II MADS-box genes in seagrasses and *P. acutifolius*, including *Arabidopsis thaliana* (AT) and *Oryza sativa* (Os) for reference. b) Gene expression patterns for type II MADS-box genes from various organs of *Z. marina*. Expression values were scaled by log2(TPM+ 1). c) Gene expression patterns for type II MADS-box genes from various organs of *P. oceanica*. Expression values were scaled by log2(TPM+ 1). d) The flowering ABCE model in *Z. marina* specifying female and male organs as proposed based on gene expression values (bar heights) from b. e) The flowering ABCE model in *P. oceanica* specifying female and male organs as proposed based on the gene expression values (bar heights) from c. f) Normalized gene copy numbers for MADS-box and pollen toolkit genes for 4 seagrasses and 19 representative non-seagrass species. Normalization for each gene family was obtained by dividing the number of genes in that gene family for a particular species by the largest gene copy number within that family (considering all species). Genes in black are absent. Taxa are arranged phylogenetically and colored by life form.

Sexual reproduction in seagrasses occurs underwater (hydrophilous) by completely submerged male and female (unisexual) flowers. Their floral structures are simplified, often having reduced, or no, sepals and petals, which may represent an adaption to hydrophilous, and mostly abiotic, pollination ^84^. However, this striking morphological adaption is not reflected by a striking loss of genes defining the well-known ABC(D)E model for floral organ-specification ^85,86^ (Supplementary Table 5.9 and Figure 4a). In *Z. marina*, the B-function (encoding *PISTILATA*) homolog seems to be mainly expressed in the staminate (“male”) flower, while two C-function homologs (*AGAMOUS; AGa* and *AGb*) were mainly expressed in the pistillate (“female”) flower (Figure 4d), suggesting involvement of the B-function only in stamen development, and C-function in carpel development. In *P. oceanica*, the expression patterns differ from those in *Z. marina*, but largely agree with previously ascribed roles in floral organ patterning: B-function *PI* and C-function *AG* homologs are highly expressed in both staminate and pistillate flowers (Figure 4e). However, in both seagrasses, one A-function homolog, *AGL6*, is highly expressed in pistillate flowers, indicating the possibility of A-function neofunctionalization, transitioning from a role in sepals and petals to one being associated with pistillate flower development. The two *SEP* E-function homologs of three seagrasses are highly expressed in pistillate and staminate flowers, indicating an essential role of these flower-specific co-factors in organ specification. The discrepancy between the floral simplification and the presence of all types of floral organ identity genes in the seagrass genomes may reflect the instability of the floral ground plan between alismatid lineages ^87^, and is possibly affected by neofunctionalisation and shifts in expression domains of floral identity genes.

Hydrophilous pollination is extremely rare outside the seagrasses, leading to the proposal that it is one of the defining features of seagrasses ^26^. The majority of seagrasses have flexible, filiform pollen in which a rigid exine layer is structurally reduced or absent ^88^, likely facilitating hydrophilous pollination. Consistent with the loss or severe reduction of the exine layer, many genes involved in the biosynthesis and secretion of the exine layer (Supplementary Note 5.9) are absent in *Z. marina* ^15^, while *C. nodosa, P. oceanica*, and *T. testudinum* show partial gene loss (Figure 4f). It will be of interest to also investigate the role of pollen-specific genes, such as an orthologs of RESTORER OF FERTILITY 1 (encoded by *RF-1*), in the evolution of hydrophylous pollination. Supplementary Figure 5.9 shows flower and pollen development toolkit gene family expansion and contraction values for 96 species, including the 90 species-transcriptome data set of Chen et al. ^16^.

## Conclusion

Seagrasses are now recognized as foundational species for invaluable ecosystems that provide multiple functions and services ^9^. They prevent erosion and hence preserve coastal seascapes, serve as biodiversity hotspots for associated animals, algae and plants, and have recently been proposed as a nature-based solution for climate mitigation owing to their carbon storage capacity in belowground biomass ^89^. Seagrasses also represent an extremely rare adaptation in the world of flowering plants, unlike (re-)adaptation to freshwater environments, which occurred at least 222 times in embryo-bearing plants ^90^. As far as is known, in part due to an extremely poor fossil record, seagrasses have evolved only on three different occasions from freshwater ancestors to (a group of) species that lives continuously submerged in a highly saline environment, including subaqueous pollination (except in *Enhalus acoroides* ^91^). Why only 84 species, spread across the three lineages, emerged in a time interval of 100 Mya, remains unresolved, but it may be related to high ocean connectivity on one hand ^92^, while within-species, ecological tolerance and phenotypic plasticity is high ^93^.

Comparative genome analysis has unveiled considerable convergence in seagrasses, but mainly for processes and pathways that have become redundant or even detrimental in a submerged marine environment. These include genes for stomata development, ethylene biosynthesis and signaling, pollen-coat formation, disease resistance, and heat shock transcription factors (HSFs). Jointly, these results illustrate that the invasion of the marine environment is associated with a significant loss of genes in multiple pathways that are no longer needed, a compelling example of “use it or lose it.”

Clear evidence of convergent positive (or gain of function) adaptation among the different lineages of seagrasses is harder to establish. Rather than unveiling major biological innovations including the rewiring of biological networks, adaptation to the marine environment seems mainly to involve the fine-tuning of many different/supportive processes that likely all had to happen in parallel, possibly explaining why the transitioning to a marine lifestyle has been exceedingly rare. For instance, adaptation of seagrasses to a marine (saline) environment was not accompanied by massive changes to individual salt tolerance traits, but rather involved more subtle changes in gene copy number and regulatory mechanisms, along with structural adaptations of the cell walls. This gradual modulation of preexisting mechanisms is consistent with the presence of multiple less extreme halophytes within alismatid families ^94^. The fine-tuning of many biological processes may also have facilitated the considerable phenotypic plasticity displayed by seagrass populations allowing their colonization from the tropics to the poles.

Many of the genes co-opted in different pathways in seagrasses seem to have been specifically retained following WGDs and WGTs that occurred long ago, suggesting important interdependencies of large-scale (or major) genome evolution events and evolutionary adaptation. Prime examples identified here are hypoxia-responsive genes, genes involved in salt tolerance, flavonoid metabolism, carbon acquisition, and C4-like photosynthesis. Therefore, the co-option of extra genes specifically retained following ancient whole genome duplications likely played a crucial role in facilitating survival in a marine environment.

We expect that the new, high-quality, seagrass genomes presented here will accelerate experimental and functional studies and contribute to transformative solutions in the management and conservation of seagrass ecosystems, which is an urgent concern in times of climate change and marine biodiversity crisis given the continuing worldwide loss of seagrass meadows.

## Methods

### Sampling metadata, DNA and RNA preparation

Whole plants from each species were collected from the field, transported to the lab in a cool box, cleaned, frozen in LN_2_ and then stored at -80°C. Collection and processing information are summarized in Supplementary Table 1.1. All samples were made with collection permits and followed the CBD-Nagoya Protocol. Care was taken to use tissue harvested from the basal area of young, clean leaves (10-cm pieces) to minimize epiphytic diatoms and bacteria If necessary. The seagrass tissues were then sent by overnight courier on dry ice to the Arizona Genomics Institute, Tucson, AZ, USA for extraction of nucleic acids (https://www.genome.arizona.edu). Quality controlled nucleic acid samples were then shipped on dry ice to the Joint Genome Institute (JGI) in Berkeley, CA, USA (https://jgi.doe.gov/) for further diagnostics and sequencing library preparation. For *P. acutifolius*, nucleic acids were extracted, QC’d and sequenced at the Max Planck-Genome-Centre Cologne, Germany (https://mpgc.mpipz.mpg.de/home/).

High Molecular Weight (HWM) DNA was extracted from young leaves of *T. testudinium, P. oceanica*, and *C. nodosa*, using the protocol of Doyle and Doyle (1987)^95^ with minor modifications. Young leaves, that had been flash frozen in LN2 and kept frozen at -80C, were ground to a fine powder in a frozen pestle and mortar with LN2 followed by very gentle extraction in CTAB buffer (that included proteinase K, PVP-40 and β-mercaptoethanol) for 20 mins at 37°C, followed by 20 mins at 50°C. Following centrifugation, the supernatant was gently extracted twice with 24:1 chloroform: iso-amyl alcohol. The upper phase was adjusted to 1/10^th^ volume with 3M Sodium acetate (pH=5.2), gently mixed, and DNA precipitated with iso-propanol. DNA was collected by centrifugation, washed with 70% EtOH, air dried for few minutes and dissolved thoroughly in 1x TE at room temperature. Size was validated by pulsed field electrophoresis. HMW DNA for *P. acutifolius* was extracted from 2 g of young leaves with the NucleoBond HMW DNA kit (Macherey Nagel). Quality was assessed with a FEMTOpulse device (Agilent) and the quantity was measured by a Quantus fluorometer (Promega).

RNA was extracted from seagrass leaves, rhizomes, roots, and flowers (Supplementary Table 1.1) with the NucleoSpin RNA Plant and Fungi Kit (Macherey-Nagel, USA), and checked for integrity by capillary electrophoresis using an Agilent (Santa Clara, CA, USA) 2100 Bioanalyzer with the Agilent RNA 6000 Nano Kit following manufacturer’s instructions. RNA was extracted from leaves and roots of *P. acutifolius* with the RNAeasys Plant Kit (Qiagen), including an on-column DNase I treatment. Quality was assessed with an Agilent Bioanalyser and the quantity was calculated by an RNA-specific kit from Quantus (Promega).

### Genome Sequencing

The genomes of *T. testudinium, P. oceanica*, and *C. nodosa* were determined following a whole genome shotgun sequencing strategy and standard sequencing protocols. Sequencing reads were produced using the Illumina NovaSeq platform and the PacBio SEQUEL II platform at the Department of Energy (DOE) Joint Genome Institute (JGI) in Walnut Creek, California, and the Hudson Alpha Institute in Huntsville, Alabama. One 400bp insert 2x150 Illumina fragment library and one HiC library was sequenced for each organism. Technical sequencing statistics are summarized in Supplementary Table 2.1.1. Prior to assembly, Illumina fragment reads were screened for PhiX contamination and reads composed of >95% simple sequences were removed. Furthermore, Illumina reads <50bp, after trimming for adapter and checking for quality (q<20), were also removed. For the Illumina sequencing, the final combined read set consisted of 4,284,278,120 high-quality reads with 161x coverage for *T. testudinium*, 6,543,657,580 high-quality reads with 327x coverage for *P. oceanica*, and 693,903,610 high-quality reads with 208x coverage for C. nodosa. For the PacBio sequencing, a total of 18 PB chemistry 3.1 chips (30-hour movie time) were sequenced with a HiFi read yield of 231.8 Gb with 51.53x coverage, 238.3 Gb with 79.44x coverage and 39.6 Gb with 79.24x coverage for *T. testudinium, P. oceanica* and *C. nodosa*, respectively.

For *P. acutifolius*, all libraries (PacBio, RNA and Tell-seq) and PacBio HiFi sequencing were performed at the Max Planck-Genome-Centre Cologne, Germany (https://mpgc.mpipz.mpg.de/home/). Short-read libraries and sequencing (RNA-seq and Tell-seq) were performed at Novogene Ltd (UK), using a NovaSeq 6000 S4 flowcell Illumina system. An Illumina-compatible was prepared with the NEBNext® Ultra™ II RNA Library Prep Kit for Illumina. PacBio-HiFi libraries were prepared according to the manual “Procedure & Checklist - Preparing HiFi SMRTbell® Libraries using SMRTbell Express Template Prep Kit 2.0” with an initial DNA fragmentation by g-Tubes (Covaris) and final library size selection on BluePippin (Sage Science). Size distribution was again controlled by FEMTOpulse (Agilent). Size-selected libraries were sequenced on a Sequel II with Binding Kit 2.0 and Sequel II Sequencing Kit 2.0 for 30 h (Pacific Biosciences). The same genomic DNA was used for TELL-seq but without fragmentation. Library preparation was done as outlined in the manual “TELL-Seq™ WGS Library Prep User Guide” (ver. November 2020). Illumina “sequencing-by-synthesis” was performed on a HiSeq 2500, 2 x 250 bp with additional index sequencing cycles to read out the unique fragment barcodes. Sequences were analyzed as recommended by Universal Sequencing Technology (UST, Canton, U.S.A). The final combined read set consisted of 54,401,190 Illumina high-quality reads with 13.4 coverage and 1,900,000 PacBio HiFi reads with 43.5 coverage (Supplementary Table 2.1.1)

### Genome assembly

For *T. testudinium, P. oceanica* and *C. nodosa*, the following assembly strategy was used: the PacBio HiFi data was assembled using HiFiAsm and subsequently polished using RACON (https://github.com/lbcb-sci/racon). Due to the high heterozygosity of our sequenced seagrasses, both haplotypes were nearly complete resulting in a genome assembly composed of a highly contiguous primary set of chromosomes and a more fragmented alternative set of chromosomes (Supplementary Figure 2.1.1). For *T. testudinium*, the initial primary assembly consisted of 1,987 contigs with a contig N50 of 483.4 Mb, and a total assembled size of 4,866.1 Mb. For *P. oceanica*, the initial primary assembly consisted of 3,470 contigs, with a contig N50 of 355.8 Mb, and a total assembled size of 3,192.0 Mb (Supplementary Table 2.1.2). For *C. nodosa*, we produced an initial primary assembly of 1,362 contigs, with a contig N50 of 18.5 Mb, and a total assembled size of 466.0 Mb (Supplementary Table 2.1.2). Misjoins in the assemblies were identified using HiC data as part of the JUICER/JuiceBox pipeline^96^ for each of the three seagrass genomes. After resolving the misjoins, the broken contigs were then oriented, ordered, and joined together with HiC data using the JUICER/JuiceBox pipeline. In *T. testudinum*, there were 5 misjoins identified in the polished primary assembly, and a total of 15 joins were applied to the primary assembly to form the final assembly consisting of 9 chromosomes. In both the *P. oceanica* and *C. nodosa* polished primary genomes, there were no misjoins identified. A total of 6 joins were applied to the primary assemblies of *P. oceanica* and *C. nodosa* to form the final assembly consisting of 10 chromosomes and 18 chromosomes, respectively. Each chromosome join is padded with 10,000 Ns. Significant telomeric sequence was identified using the (TTTAGGG)_n_ repeat, and care was taken to make sure that contigs terminating in telomere were properly oriented in the production assembly. The remaining scaffolds were screened against bacterial proteins, organelle sequences, GenBank nr and removed if found to be a contaminant. Heterozygous SNP/indel phasing errors were corrected using the HiFi data (51.53x for *T. testudinum*, 79.44x for *P. oceanica* and 79.24x for *C. nodosa*). Finally, homozygous SNPs and indels were corrected in the releases using Illumina reads (2x150, 400bp insert). A total of 2,613 homozygous SNPs and 82,421 homozygous indels were corrected in *T. testudinum*. A total of 1,643 homozygous SNPs and 100,570 homozygous indels were corrected in *P. oceanica* and total of 1,426 homozygous SNPs and 12,492 homozygous indels were corrected in the *C. nodosa*. Due to the high heterozygosity of the three genomes, both haplotypes of each chromosome were well represented in the assemblies. The primary set of chromosomes were constructed from the primary assembly, while an alternative set of chromosomes were constructed from the alternate assembly. Chromosomes for the alternate haplotype were then oriented, ordered, and joined together using synteny from the primary chromosomes (Supplementary Table 2.1.3).

For *Potamogeton acutifolius*, we used HiFiAsm ^97^ to assemble a draft genome assembly of a total length of 611 Mb with N50 = 3.09 Mb and scaffolded it further with Tell-seq data (linked reads; bioRxiv 2019, 852947) using the ARCS software ^98^ and reaching final N50 = 4.45 Mb (6,705 scaffolds in total, the length of the largest scaffold = 31.2 Mb).

### Genome annotation

#### Structural and functional annotation of genes

Our annotation pipeline integrated three independent approaches, the first one based on transcriptome data, the second one being an *ab initio* prediction and the third based on protein homology. Both RNA-seq and Iso-seq data from different tissues (Supplementary Table 3.2.1 – Supplementary Table 3.2.4) were used to aid the structural annotation and RNA-seq datasets were first mapped using Hisat2 (v2.1.0, arguments dta) ^99^ and subsequently assembled into transcript sequences by Stringtie2 ^100^, whereas Iso-seq sequences were aligned to the seagrass genome using GMAP ^101^. All transcripts from RNA-seq and Iso-seq were combined using Cuffcompare (v2.2.1) and subsequently merged with Stringtie2 (arguments merge -m 150) to remove fragments and redundant structures ^100^. Transdecoder v5.0.2 (github.com/TransDecoder) was then used to predict protein sequences with diamond v2.0.14 results (evalue 1e-5 max-target-seqs 1 -f 6). BARKER v2.1.2 ^102^ was used for *ab initio* gene prediction using model training based on RNA-seq data. Homology-based annotation was based on the protein sequences from related species (*Z. marina* v1.0, *Spirodela polyrhiza, Oryza sativa* and *Arabidopsis thaliana*) as query sequences to search the reference genome using TBLASTN with e-value ≤1e^–5^, then regions mapped by these query sequences were subjected to Exonerate to generate putative transcripts. Additionally, an independent, homology-based gene annotation was performed using GeMoMa ^103^ using the same species with TBLASTN.

All structural gene annotations were joined with EvidenceModeller ^104^ v1.1.1, and BUSCO v4.0.4 (Benchmarking Universal Single-Copy Orthologs) ^105^ was used to assess the quality of the annotation results. Finally, we used GenomeView ^106^ to do the gene curations manually based on the RNA-seq and Iso-seq data. Putative gene functions were identified using InterProScan ^107^ with different databases, including PFAM, Gene3D, PANTHER, CDD, SUPERFAMILY, ProSite and GO. Meanwhile, functional annotation of these predicted genes was obtained by aligning the protein sequences of these genes against the sequences in public protein databases and the UniProt database using BLASTP with the e-value ≤1e − 5.

#### Annotation of non-protein coding RNA families

Finished genome assemblies and annotations (genome.fasta and genome.gff files for *Z. marina, C. nodosa, P. oceanica, T. testudinum* and *P. acutifolius*) were uploaded to, and later downloaded from, JGI Phytozome ^108^. Infernal v1.1.4 (Dec 2020) ^109^ was used to perform sequence similarity searches of each genome sequence versus the RFAM database (RNA families database, Dec2021) ^110^. The output from Infernal was filtered, keeping only the hits with an E-value threshold E<0.01. A second filtering step was performed to remove redundant information, i.e., overlapping matches with similar hits. A third filtering step was performed by retaining all the hits matching with a coverage of at least 95% and removing all partial/fragmented matches with incomplete hits from the reference collection. rRNA, tRNA, snoRNA and miRNA regions were selected and annotated in the annotation.jff files for each species. An updated functional annotation including the identified loci in the genomes was performed by scanning the Uniprot database ^111^ with BLASTp ^3^. Introns and the corresponding sequence regions were extracted by GenomeTools ^112^ and Bedtools ^113^ programs. The functional annotation of the long introns (>= 20kb) was performed by similarity searches in the NCBI nucleotide ^114^ database with the BLASTn tool ^3^.

#### Annotation of repeats and transposable elements (TEs)

Two complementary approaches were used to identify repetitive DNA sequences. First, a *de novo* repeat identification was carried out with RepeatModeler v2.0.1 (https://www.repeatmasker.org/RepeatModeler/) based on the default TE Rfam database, followed by RepeatMasker v4.1 (https://www.repeatmasker.org/) to discover and classify repeats based on the custom repeat libraries from RepeatModeler v2.0.1. Second, LTR_Finder ^115^ (v1.0.7), LTR_harvest ^116^ from genometools (v1.5.9) and LTR_retriever ^117^ (v2.9.0) were used to identify and trace the LTR elements, which were subsequently characterized at clade/lineage level by searching coding domains within the sequences, using the tool Domain based ANnotation of Transposable Elements (DANTE) (https://github.com/kavonrtep/dante). Transposable elements not classified by RepeatModeler were analyzed using DeepTE ^118^. We merged the libraries from RepeatModeler, LTR_retriever and DeepTE using USEARCH ^119^ with 80% identity as the minimum threshold for combining similar sequences into the final non-redundant de novo repeat library. Finally, we used RepeatMasker v4.1.0 (-e rmblast -gff -xsmall -s -norna -no_is - lib) to identify and classify repeats in the genome assemblies of seagrasses and *Potamogeton*.

#### Dating bursts of repeats in seagrass genomes

The identification of high-quality intact LTR-RTs and the calculation of insertion age for intact LTR-RTs were carried out using LTR_retriever (v2.9.0), using the formula T=K/2r. The nucleotide substitution rate “r” was set to 1.3e-8 substitutions per site per year ^120^.

### Identifying Whole Genome Duplications

#### K_S_ age distributions and gene tree-species tree reconciliation

K_s_ age distribution analysis was performed using the wgd package ^121^. Anchor pairs (i.e., paralogous genes lying in collinear or syntenic regions of the genome) were obtained using i-ADHoRe ^122^. Ks distribution analysis was also performed using the KSRATES software ^123^, which locates ancient polyploidization events with respect to speciation events within a phylogeny, comparing paralog and ortholog K_S_ distributions, while correcting for substitution rate differences across the involved lineages (see Supplementary Note 4.2.1).

OrthoFinder ^124^ was used to build orthologous gene families. For each orthogroup, a multiple sequence alignment (MSA) based on amino acid sequences was obtained using PRANK ^125^ and then used as input for Markov Chain Monte Carlo (MCMC) analysis in MrBayes ^126^. A time-calibrated species tree was inferred by MCMCtree from the PAML package ^127^, using reference speciation times of 42–52 million years ago (MYA) for the divergence between *Oryzae sativa* and *Brachypodium distachyon*, 118-129 MYA for that between *Spirodela polyrhiza* and *Z. marina*, and 130-140 for that between *Spirodela* and other terrestrial monocots ^128^. A gene duplication-loss (DL)+WGD model, under critical and relaxed branch-specific rates, was implemented for the inference of the significance and corresponding retention rates of the assumed WGD events under Bayesian inference ^25^. (see Supplementary Note 4.2.2)

#### Absolute dating of WGDs

Absolute dating of WGD events followed an approach previously described for *Zostera marina* ^15^. Paralogous gene pairs located in duplicated segments (so-called anchors) and duplicated pairs lying under the WGD peak (so-called peak-based duplicates) were collected for phylogenetic dating. Anchors, which are assumed to correspond to the most recent WGD, were detected using i-ADHoRe 3.0 ^122^. For each WGD paralogous pair, an orthogroup was created that included the two paralogues plus several orthologues from other plant species, as identified by InParanoid (v. 4.1) ^129^, using a broad taxonomic sampling. Gene duplicates were then dated using the BEAST v. 1.7 package ^130^ under an uncorrelated relaxed clock model with the LG+G (four rate categories) evolutionary model. A starting tree with branch lengths satisfying all fossil-prior-constraints was created according to the consensus APGIII phylogeny. Fossil calibrations were implemented using log-normal calibration priors (see Supplementary Note 4.2.3).

#### Time-calibrated tree construction

Protein sets were collected for 23 species (see Supplementary Note 4.3). These species were selected as representatives for monocots and eudicots, and representing different habitats from terrestrial, freshwater-floating, freshwater-submerged, to marine-submerged. Orthofinder v2.3 ^131^ was used to delineate gene families with mcl inflation factor 3.0. All-versus-all Diamond blast with an E-value cutoff of 1e−05 was performed and orthologous genes were clustered using OrthoFinder. Single-copy orthologous genes were extracted from the clustering results. MAFFT ^132^) with default parameters was used to perform multiple sequence alignment of protein sequences for each set of single-copy orthologous genes, and to transform the protein sequence alignments into codon alignments after removing the poorly aligned or divergent regions using trimAl ^133^. The resulting codon alignments from all single copy orthologs were then concatenated into one supergene for species phylogenetic analysis. A maximum-likelihood phylogenetic tree of single-copy protein alignments and codon alignments was constructed using IQ-TREE ^134^ with the GTR+G model and 1,000 bootstrap replicates. Divergence times between species were estimated using MCMCtree from the PAML package under the GTR+G model (see Supplementary Note 4.3).

#### Gene family comparisons

Gene families analyzed in the paper were searched in the output from Orthofinder and a master table was compiled to show the detailed information for each orthogroup, which is defined as the group of genes from multiple species descended from a single gene in the last common ancestor. For the superfamilies, we used the phylogenetic tree to further classify them into subfamilies. We adopted a custom criterion to assess the expansion and contraction of gene families. If the average gene number in seagrasses increased or reduced by >40% compared to non-seagrass species, we called it expansion or contraction. Syntenic analysis of genes are performed using MCScanX ^135^ and i-ADHoRe ^122^. Lastly, circos plots were drawn using Circos ^136^.

## Extended Data

**Figure:**
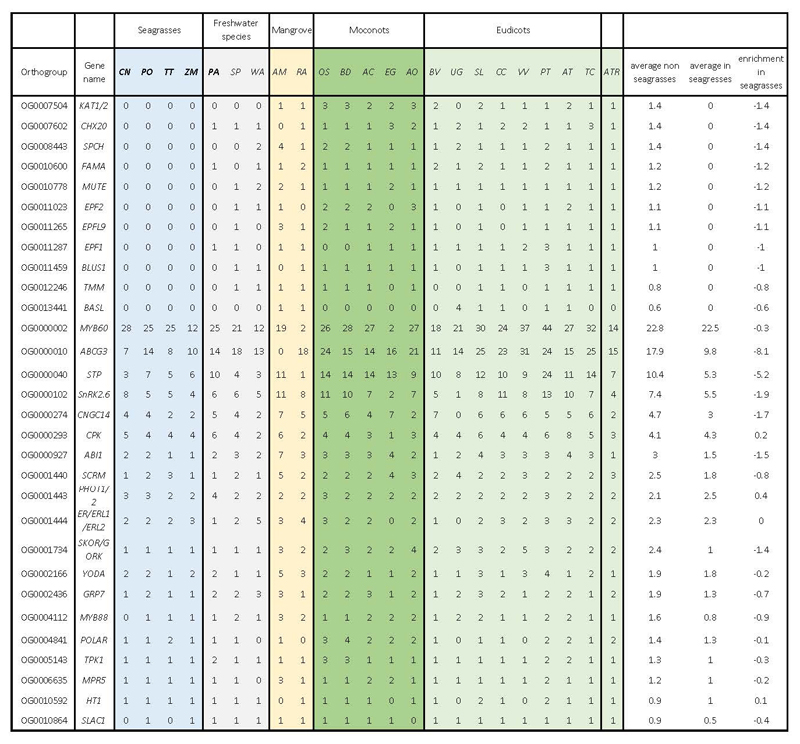


**Figure:**
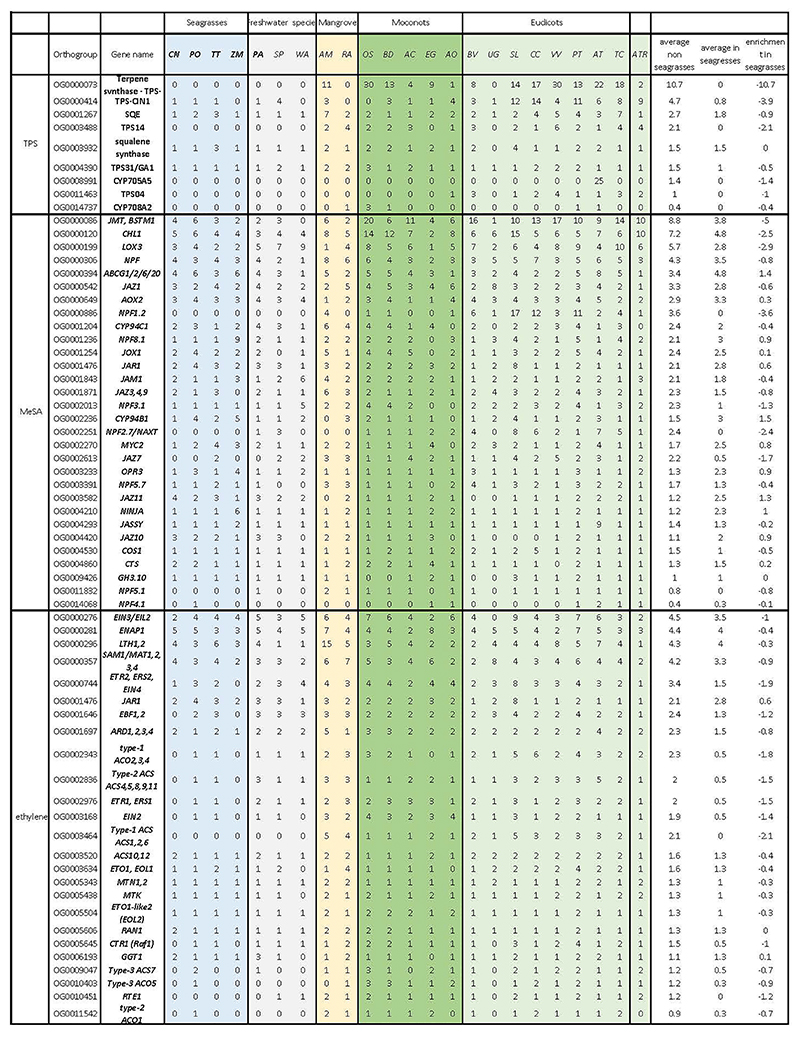


**Figure:**
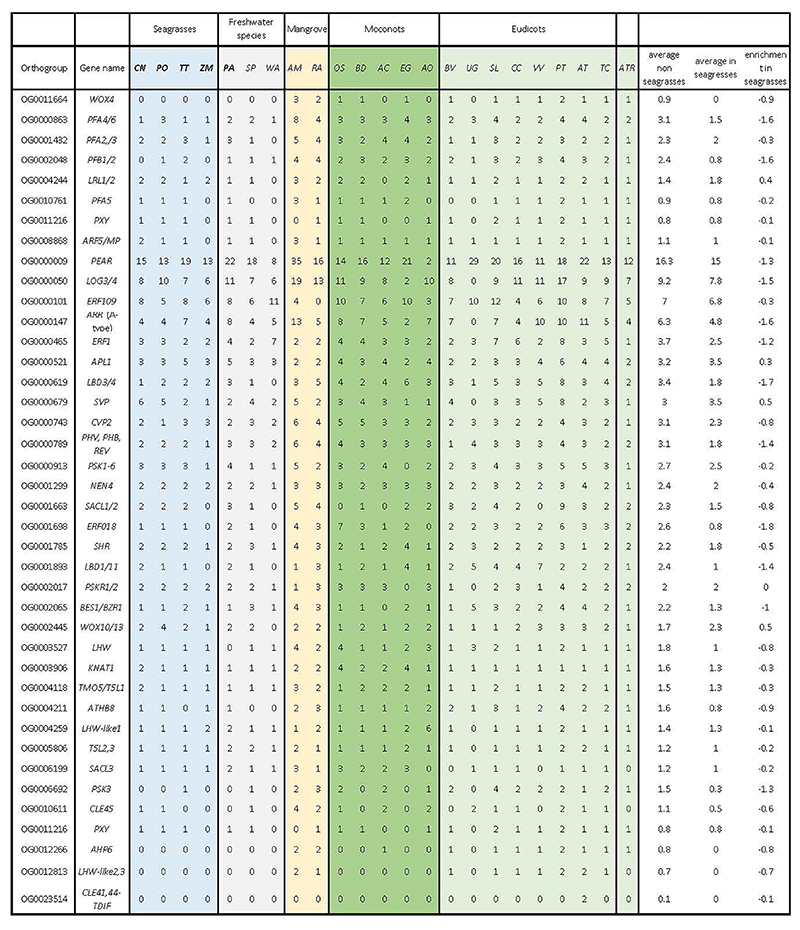


**Figure:**
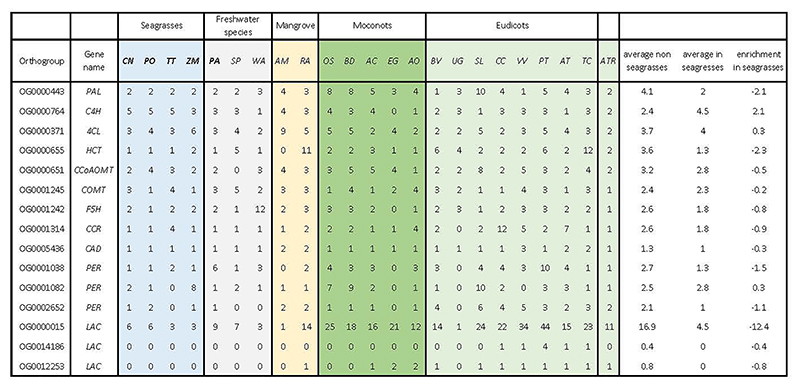


**Figure:**
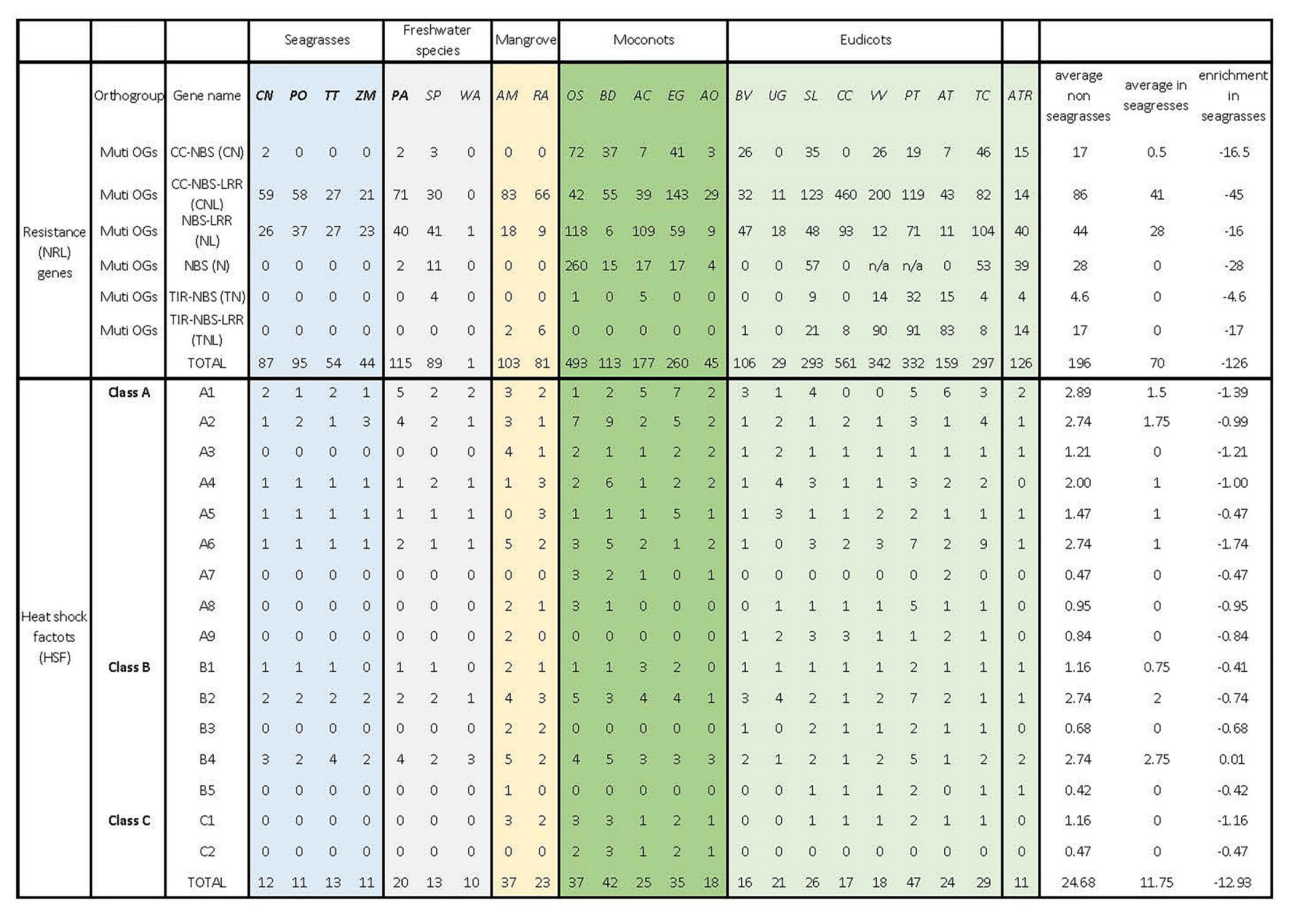


**Figure:**
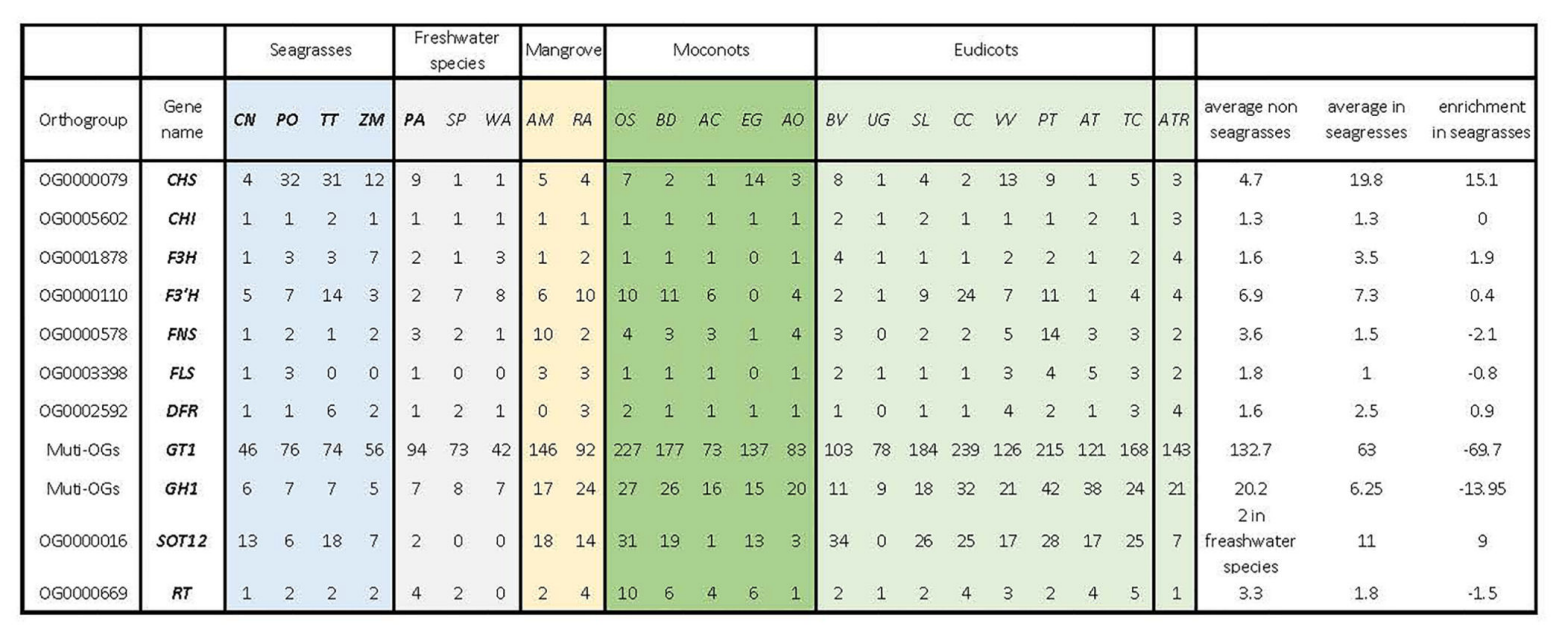


**Figure:**
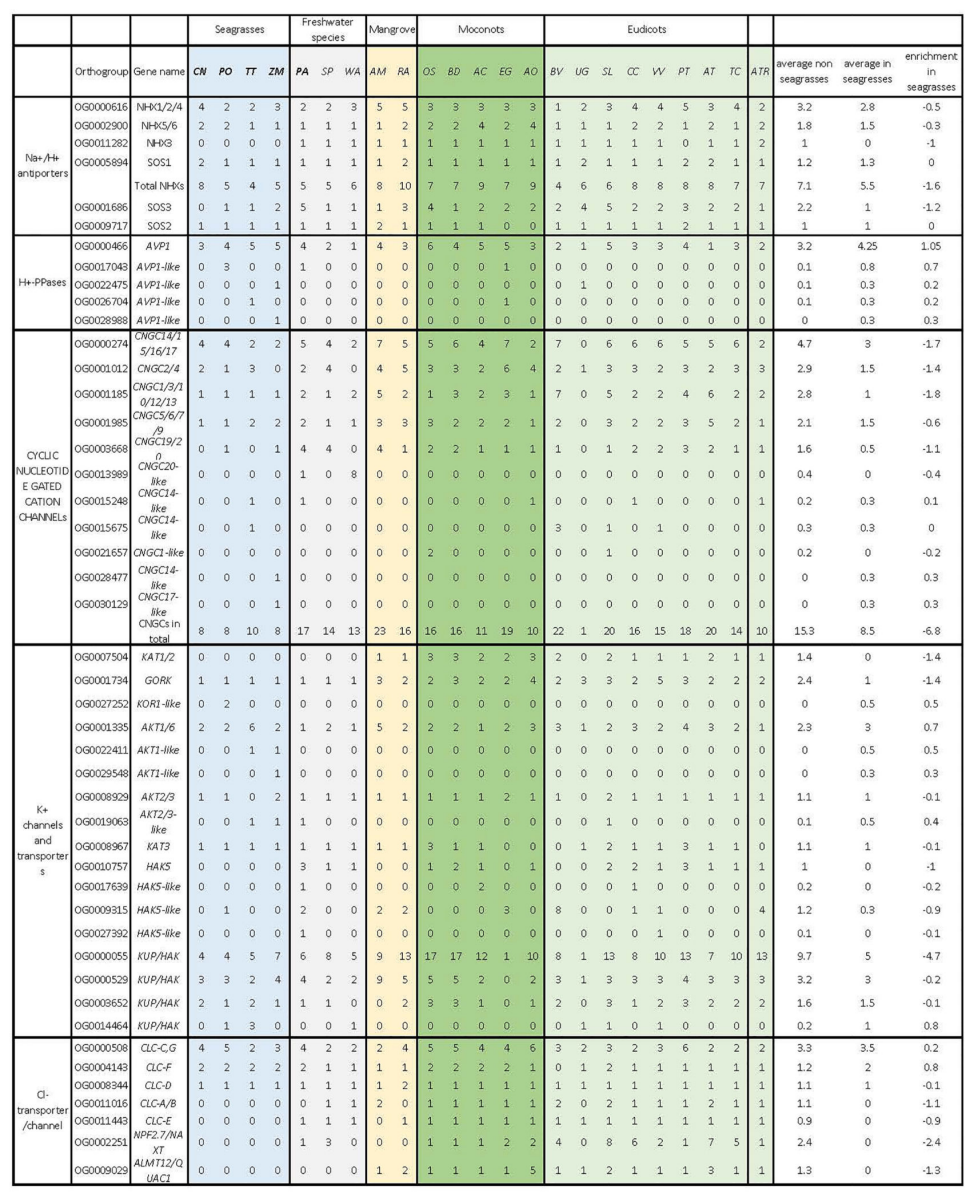


**Figure:**
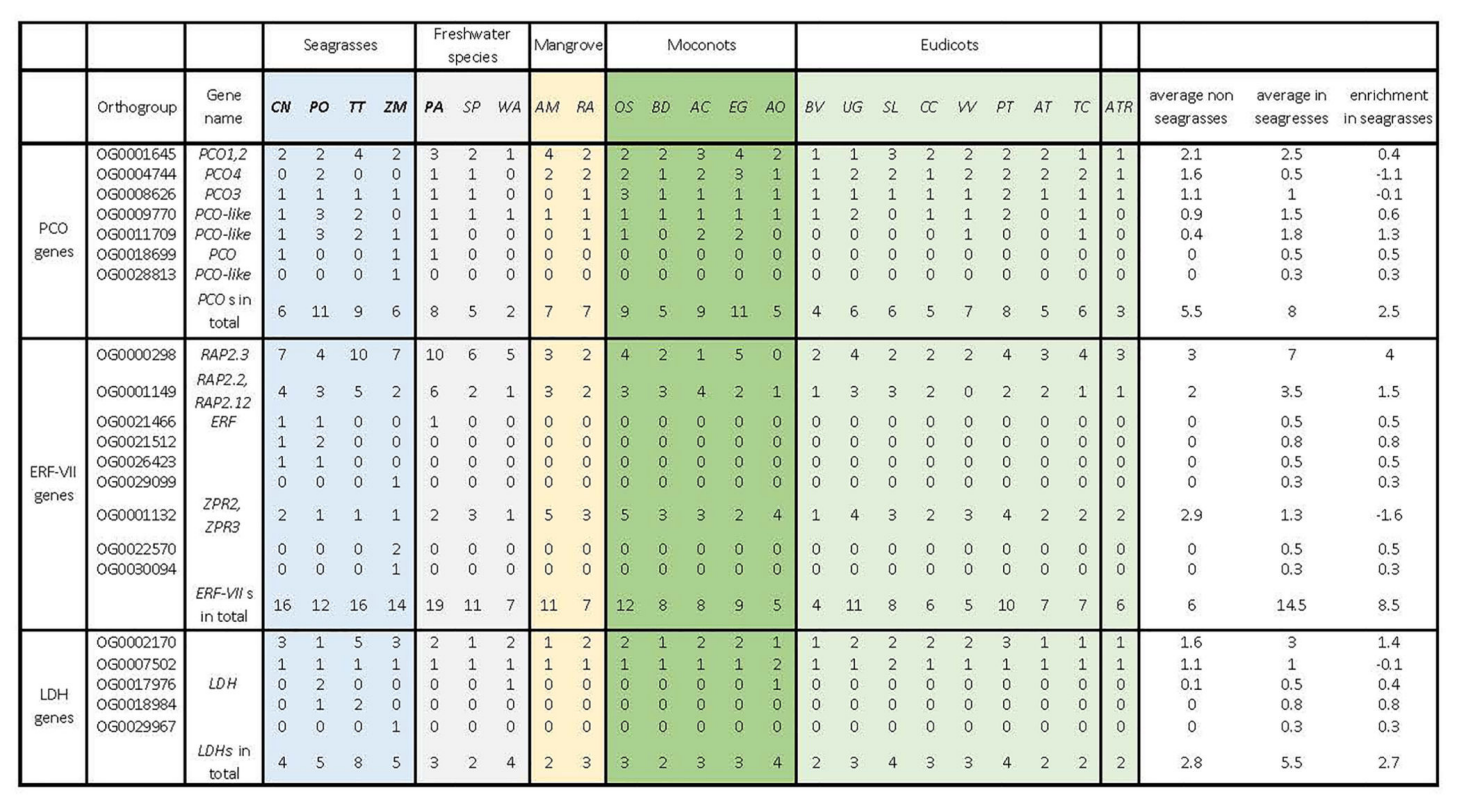


**Figure:**
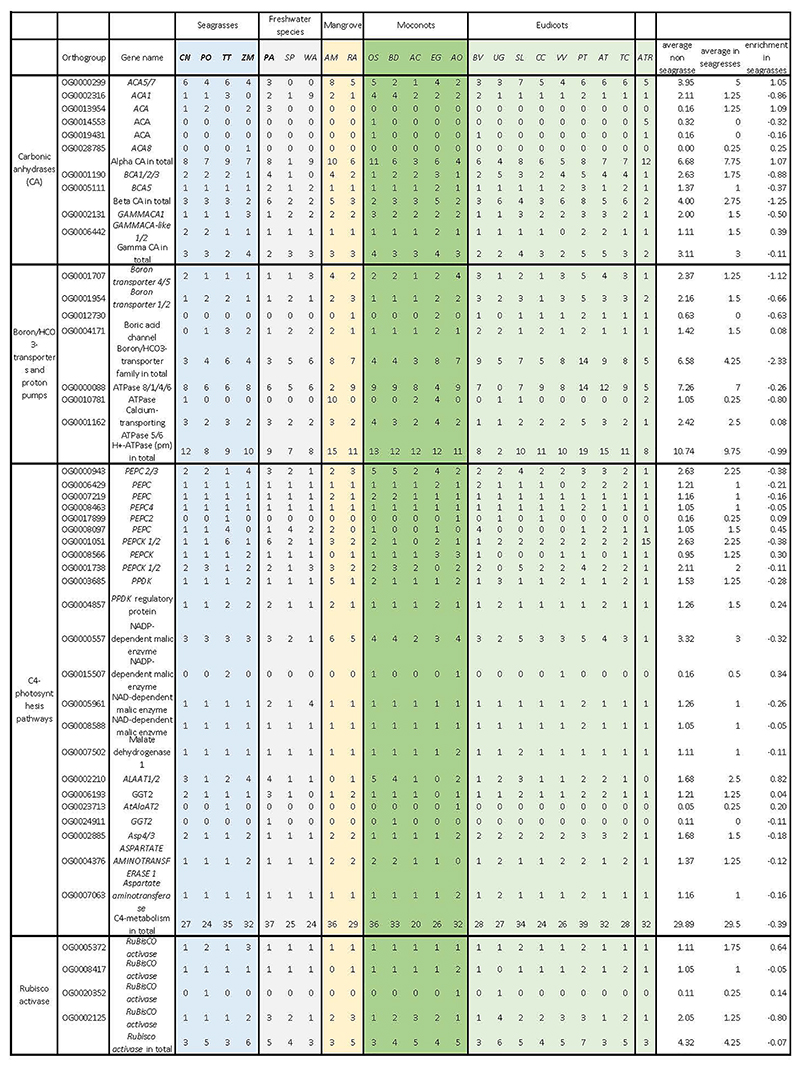


**Figure:**
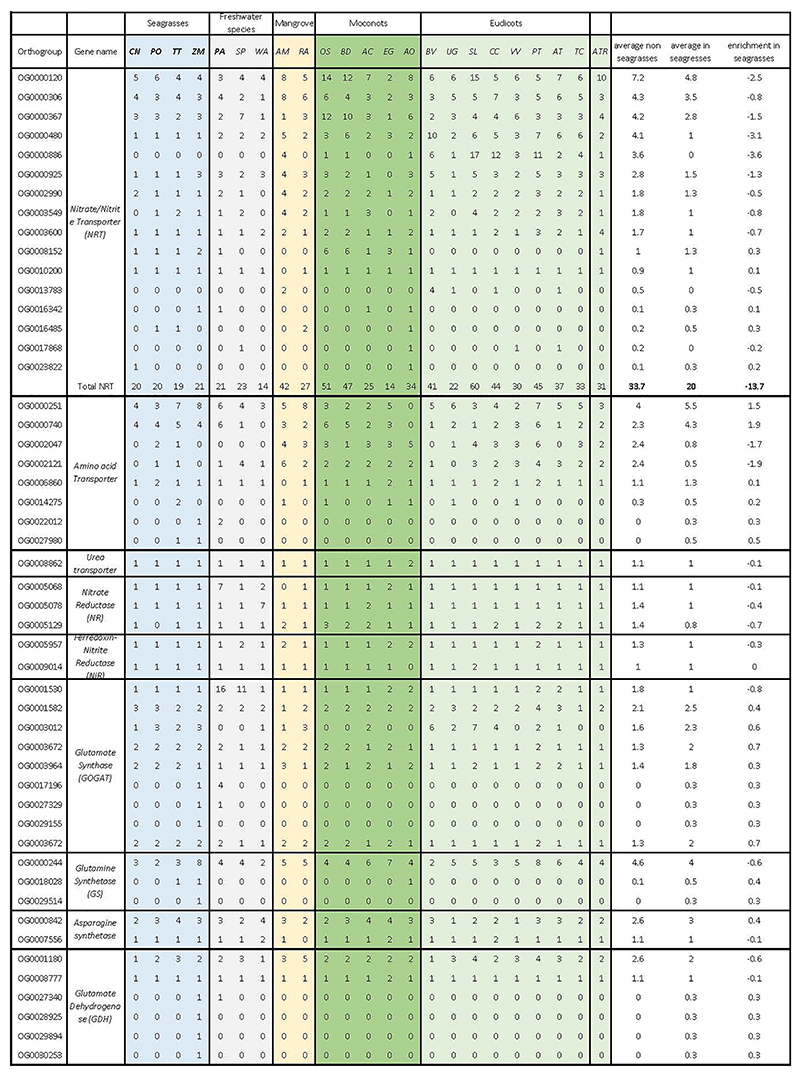


## Supplementary Material

Extended data figure 1

Extended data figure 4

Extended data figure 5

Extended data figure 6

Extended data figure 7

Extended data figure 8

Extended data figure 9

Extended data figure 10

Supplementary information

## Data Availability

The DNA sequencing data for *C. nodosa* genome assembly has been deposited in the NCBI databases under the BioProject PRJNA1041560 via the link: https://www.ncbi.nlm.nih.gov/bioproject/?term=PRJNA1041560 All assemblies and annotations for all seagrass species discussed in the current paper can be found at https://bioinformatics.psb.ugent.be/gdb/seagrasses/. Transcriptome data (including raw data and clean data) and sequencing QC Reports for *C. nodosa* can be found at https://genome.jgi.doe.gov/portal/pages/dynamicOrganismDownload.jsf?organism=Cymnodnscriptome_2; transcriptome data and sequencing QC Reports for *P. oceanica* can be found at https://genome.jgi.doe.gov/portal/pages/dynamicOrganismDownload.jsf?organism=Posocenscriptome_2; transcriptome data and sequencing QC Reports for *T. testudinum* can be found at https://genome.jgi.doe.gov/portal/pages/dynamicOrganismDownload.jsf?organism=Thatesnscriptome_4; transcriptome data for *Z. marina* is from Jeanine et al. (2016). For the public databases, RFAM database v14.7 can be downloaded at https://ftp.ebi.ac.uk/pub/databases/Rfam/14.7/; UniProt database can be accessed from the web at http://www.uniprot.org and downloaded from http://www.uniprot.org/downloads; NCBI nucleotide database can be accessed via https://www.ncbi.nlm.nih.gov/
