# Supplementary figures and images for: Seagrass genomes reveal ancient polyploidy and adaptations to the marine environment
Source: Nat Plants. Author manuscript; Available in PMC 2024 Aug 1. (PMC7615686; doi:10.1038/s41477-023-01608-5)

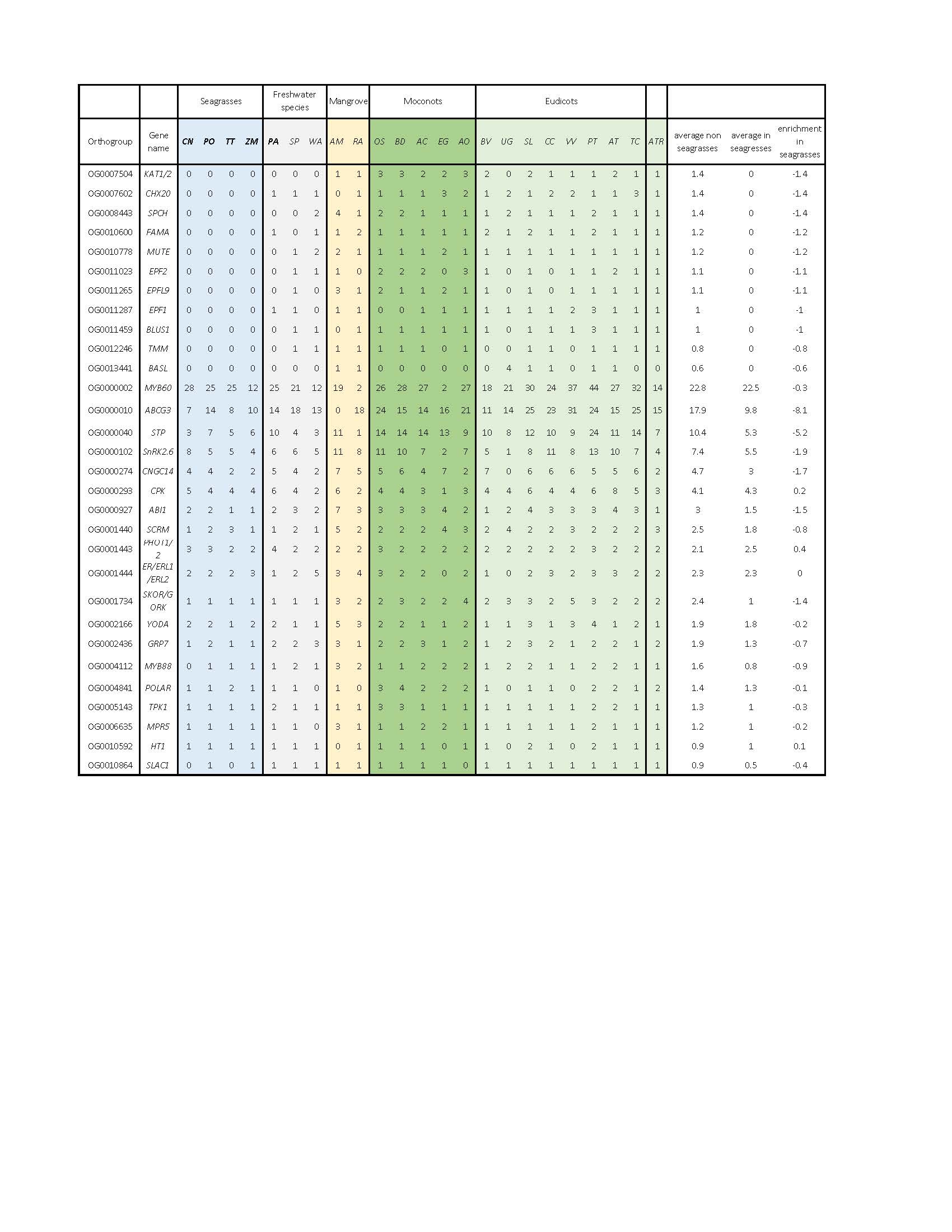

Supplement: Extended data figure 1 [file EMS194050-supplement-Extended_data_figure_1.jpg]

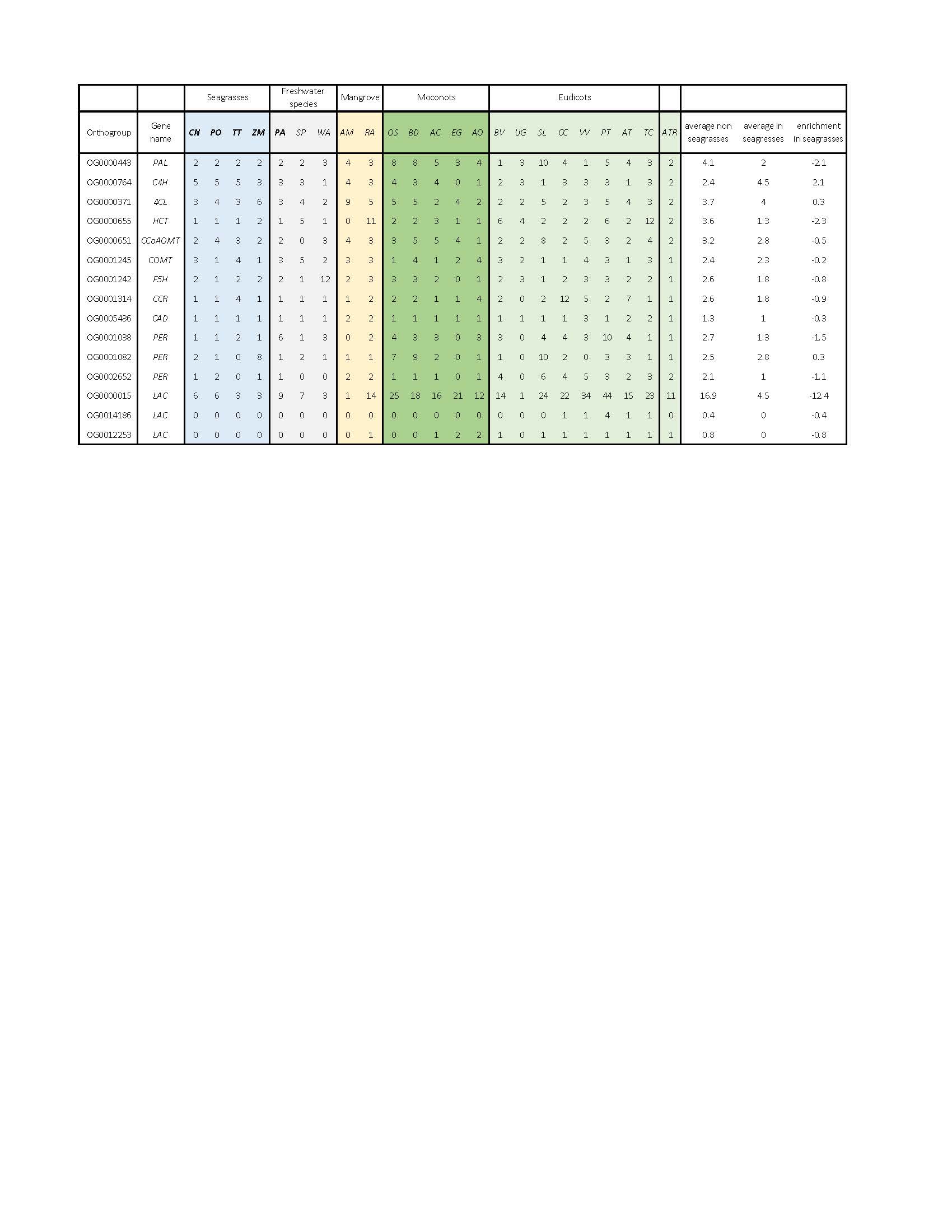

Supplement: Extended data figure 4 [file EMS194050-supplement-Extended_data_figure_4.jpg]

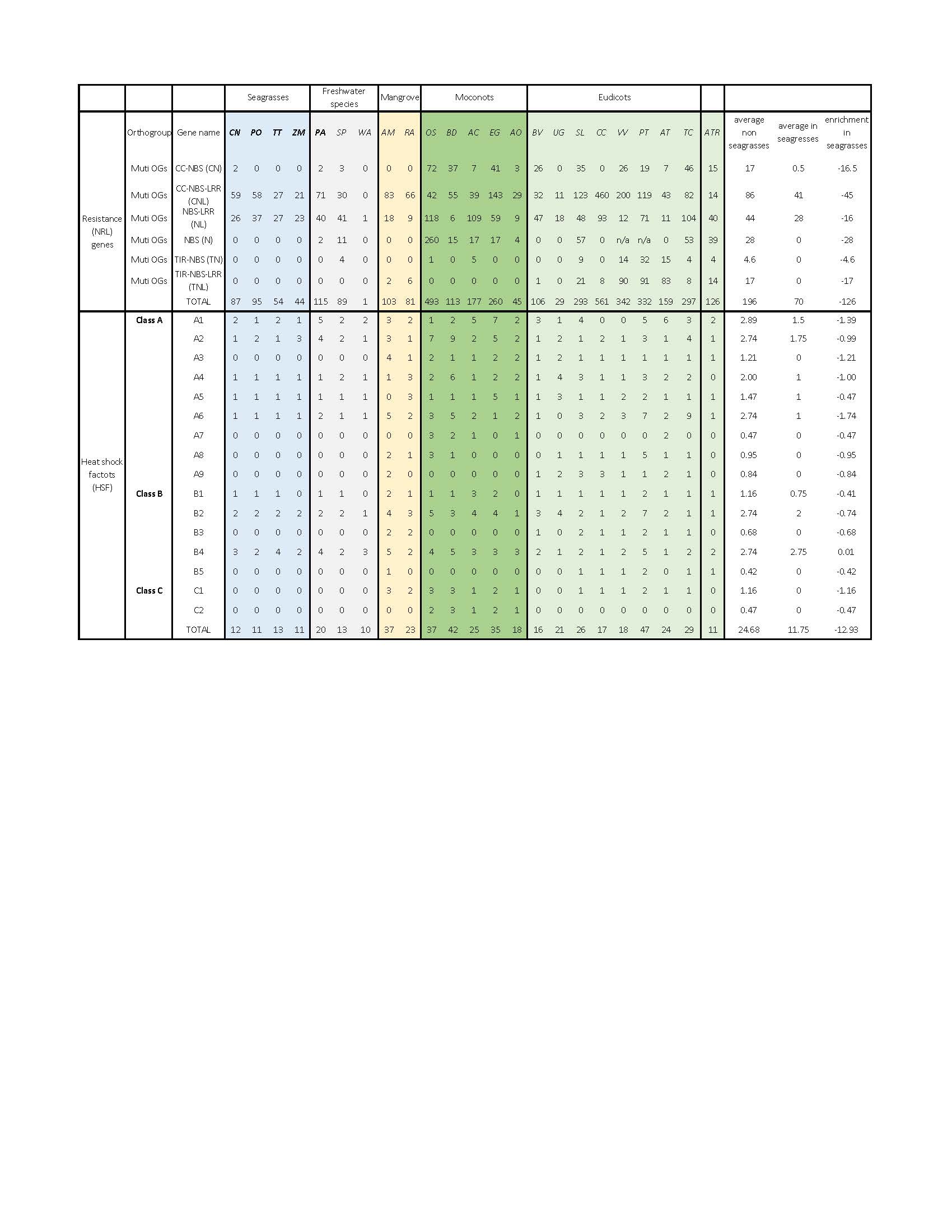

Supplement: Extended data figure 5 [file EMS194050-supplement-Extended_data_figure_5.jpg]

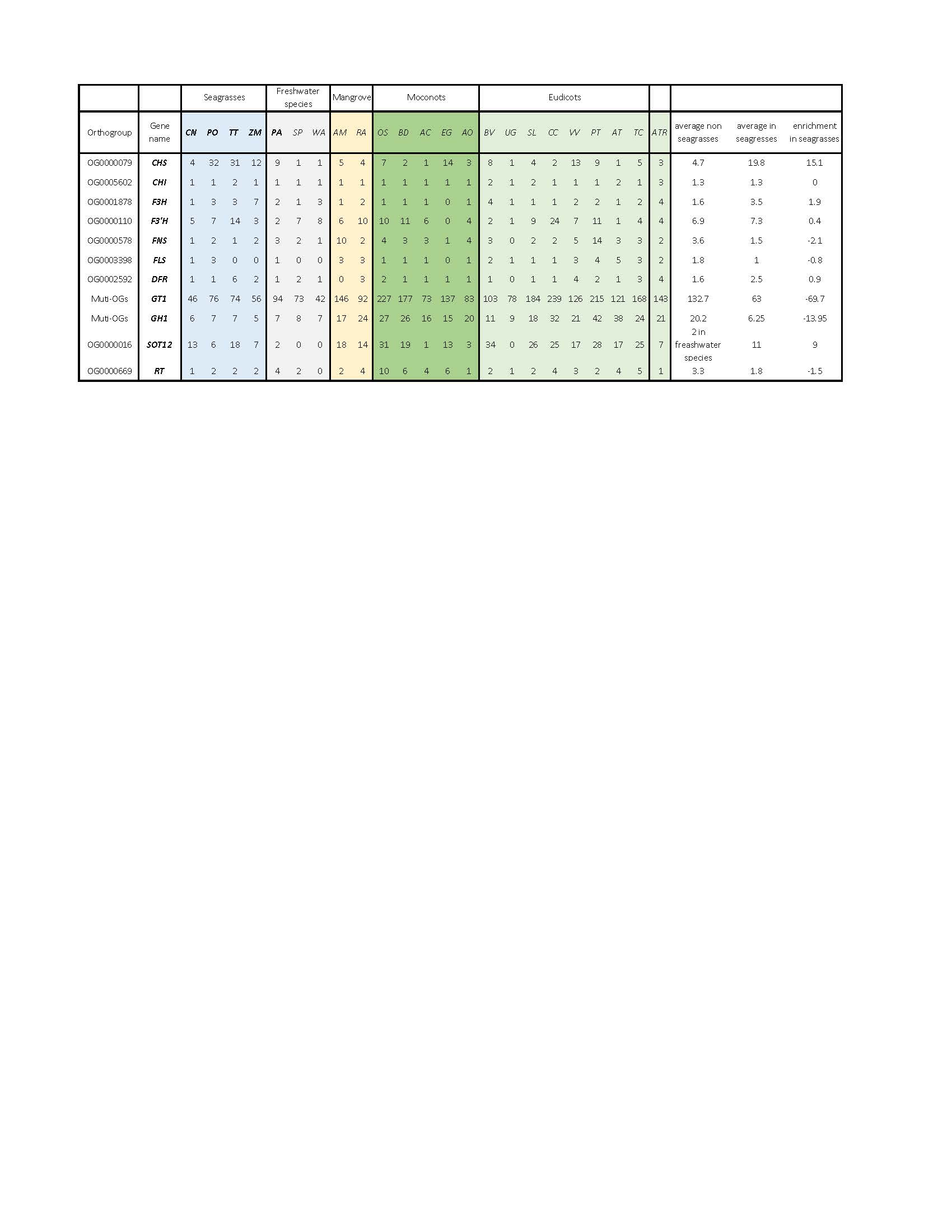

Supplement: Extended data figure 6 [file EMS194050-supplement-Extended_data_figure_6.jpg]

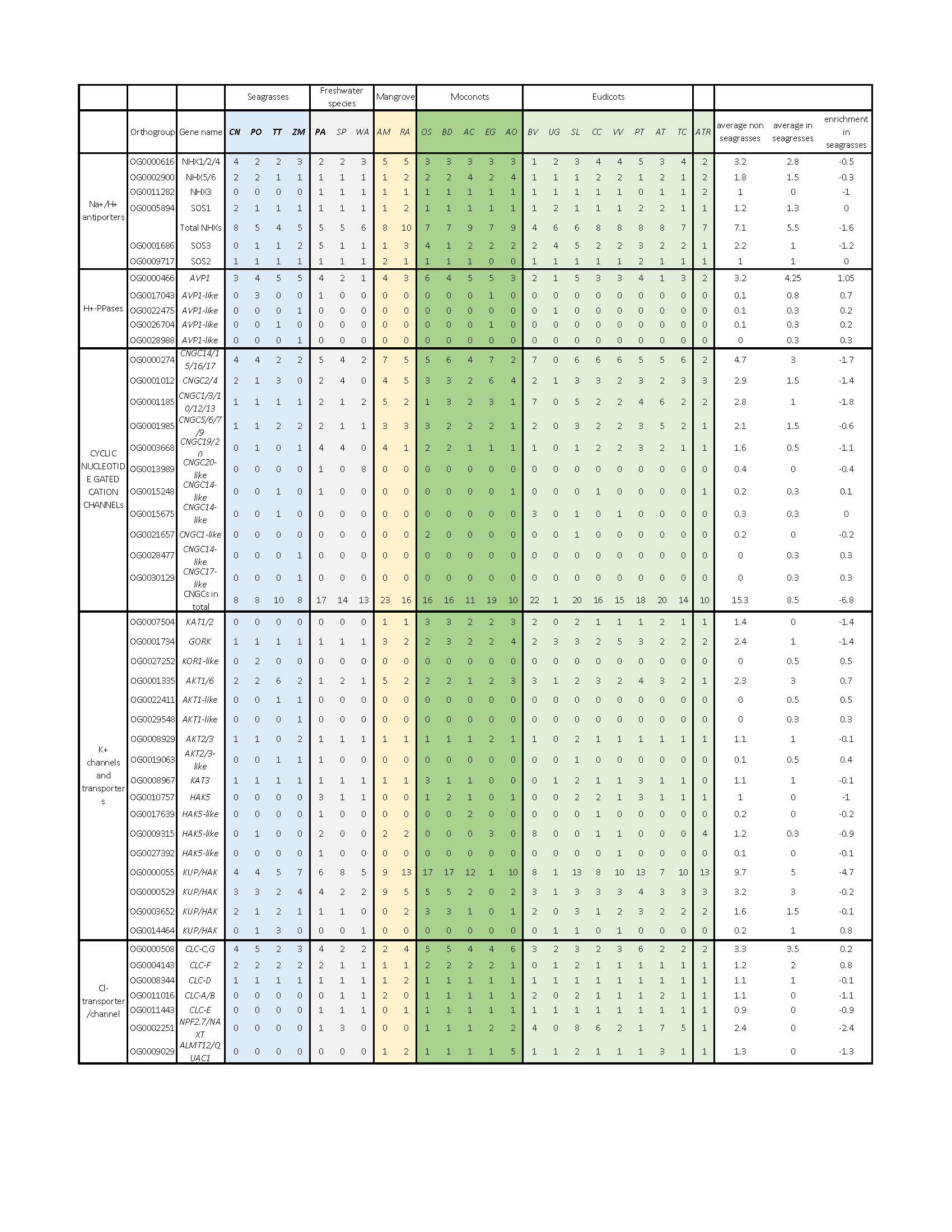

Supplement: Extended data figure 7 [file EMS194050-supplement-Extended_data_figure_7.jpg]

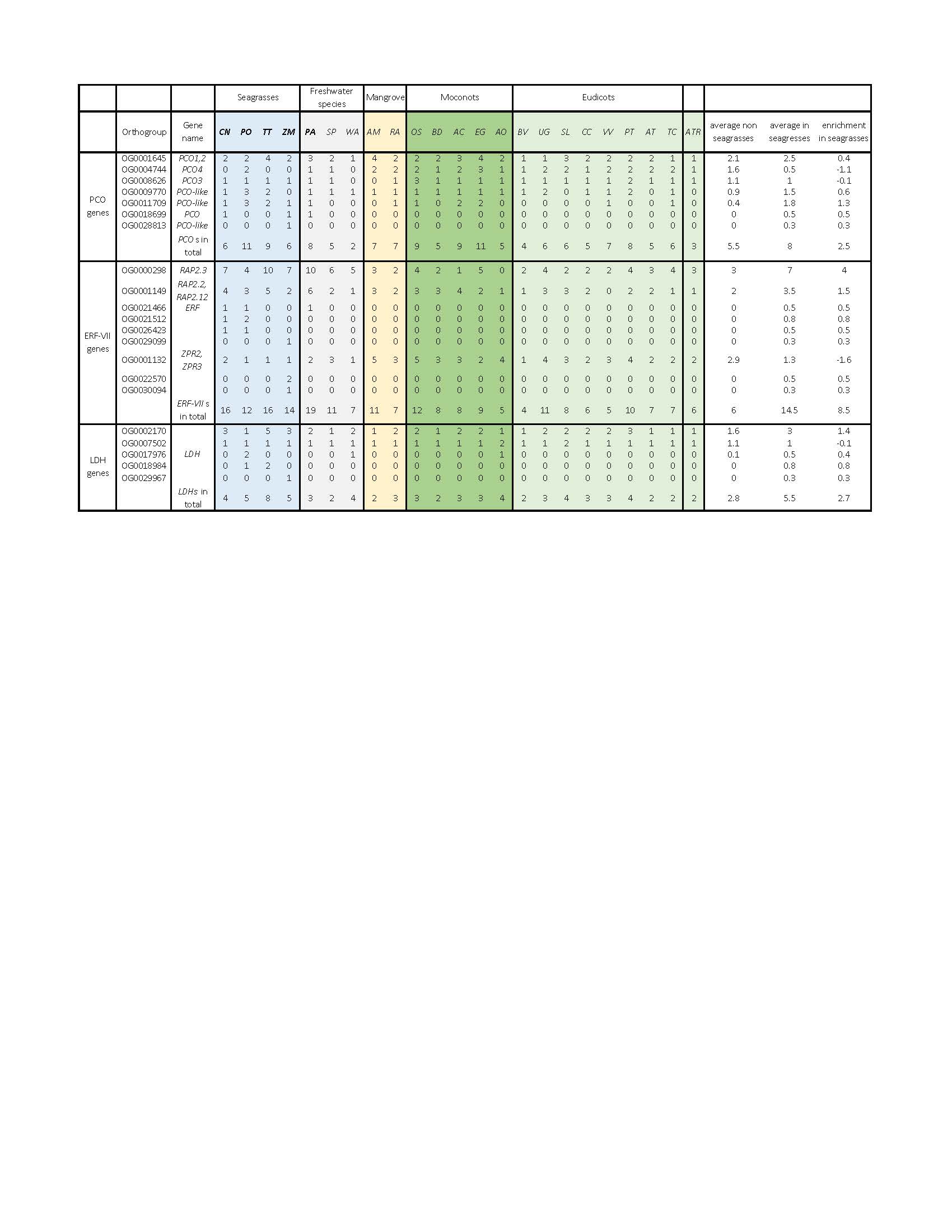

Supplement: Extended data figure 8 [file EMS194050-supplement-Extended_data_figure_8.jpg]

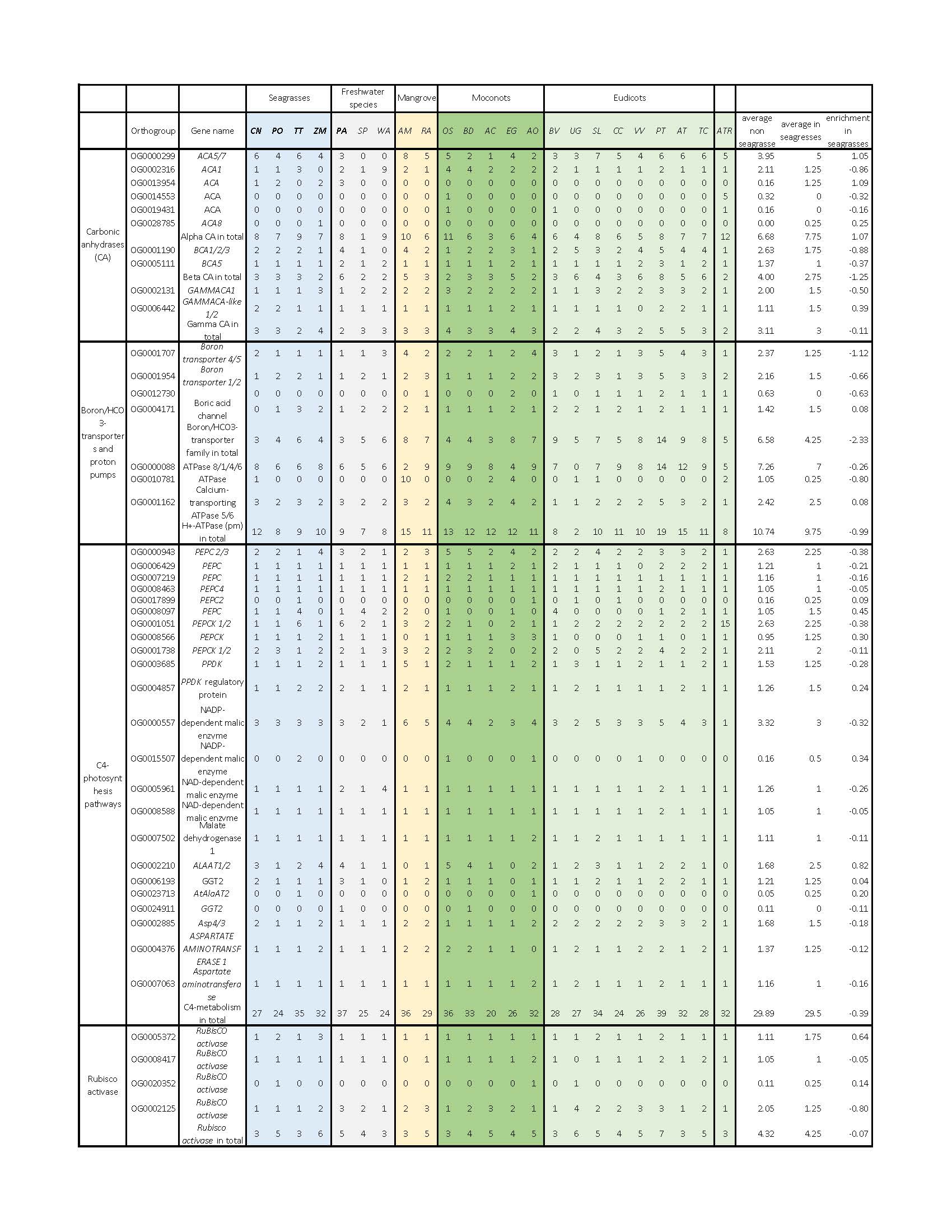

Supplement: Extended data figure 9 [file EMS194050-supplement-Extended_data_figure_9.jpg]

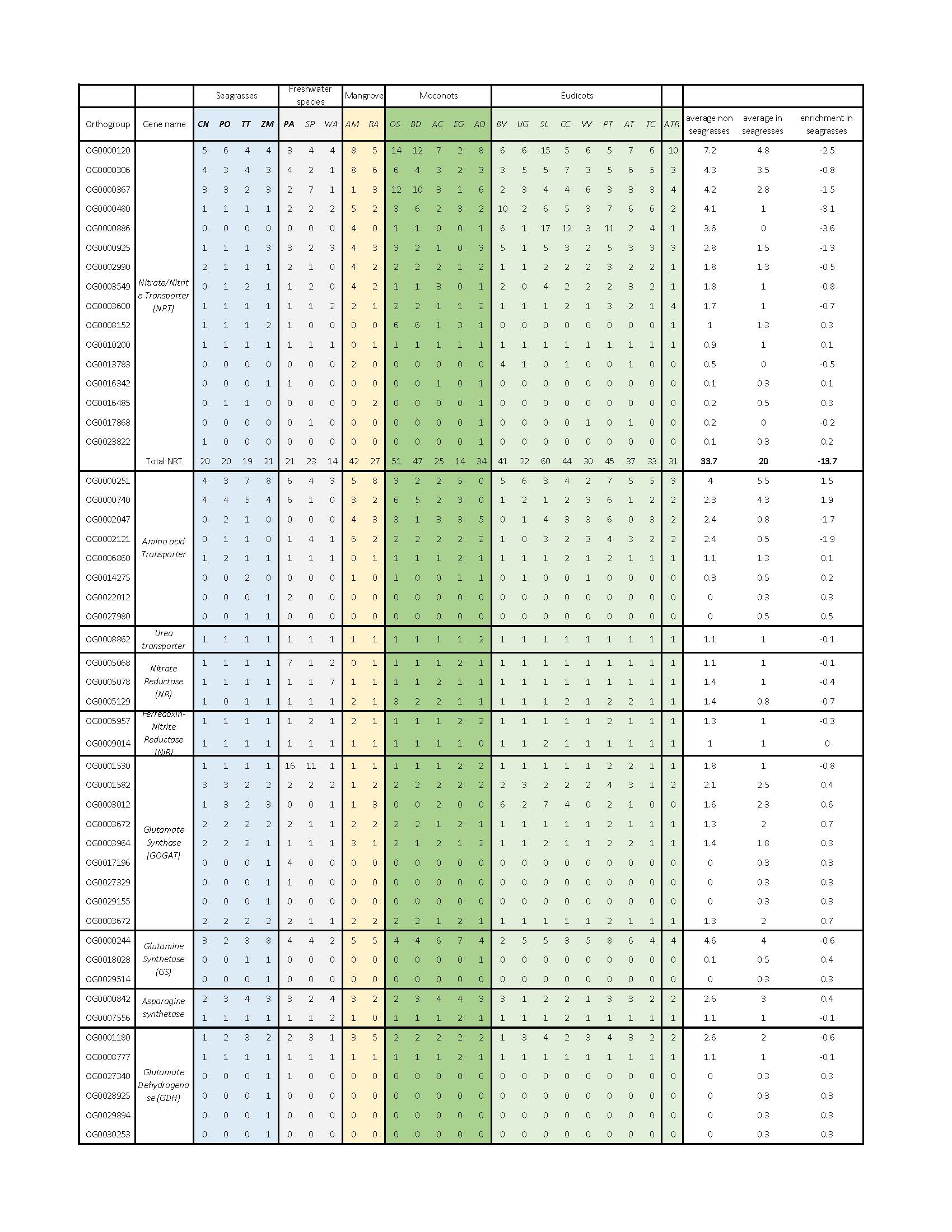

Supplement: Extended data figure 10 [file EMS194050-supplement-Extended_data_figure_10.jpg]
