# Supplementary material for: Seagrass genomes reveal ancient polyploidy and adaptations to the marine environment
Source: Nat Plants. Author manuscript; Available in PMC 2024 Aug 1. (PMC7615686; doi:10.1038/s41477-023-01608-5)
Supplement: Supplementary information [file EMS194050-supplement-Supplementary_information.pdf]

Supplementary Notes, Tables, and Figures

TABLE OF CONTENTS

1. SAMPLING SPECIES, METADATA, AND DNA AND RNA PREPARATION 5

Supplementary Figure 1.1 Phylogenetic tree of Alismatales, including seagrasses. 5

Supplementary Table 1.1 Plant material metadata. 6

2. GENOME SEQUENCING AND ASSEMBLIES 7

2.1 Nuclear genomes 7

Supplementary Table 2.1.1 Genomic libraries included in each seagrass genome assembly and their respective assembled sequence coverage levels in the final release. 7

Supplementary Figure 2.1.1 Genome assembly pipeline used for *T. testudinum*, *P. oceanica* and *C. nodosa* . 7

Supplementary Table 2.1.2 Summary statistics of the initial output of the primary RACON polished HiFiAsm assembly. 8

Supplementary Note 2.1 Final Primary assemblies for main and alternate haplotypes. 9

Supplementary Table 2.1.3 Final summary primary and alternate assembly statistics for each chromosome-scale assembly (see for further details on *Zostera marina* v3.1). 10

Supplementary Figure 2.1.2 Distribution of the genomic features for the 26 largest scaffolds of *P. acutifolius*. 11

Supplementary Table 2.1.4. Primary genome assembly, annotation statistics and BUSCO completeness assessment of protein coding sequences. See Supplementary Table 2.1.3 for additional details for the alternate haplotypes 12

2.2 Chloroplast genomes 13

Supplementary Note 2.2 Chloroplast genome assemblies and annotations 13

Supplementary Figure 2.2.1 The complete C. nodosa chloroplast genome. 13

Supplementary Figure 2.2.2 The complete *Z. marina* chloroplast genome. 14

Supplementary Figure 2.2.3 The complete *T. testudinum* chloroplast genome. 15

Supplementary Figure 2.2.4 The complete *P. oceanica* chloroplast genome. 16

2.3 Mitochondrial genomes 16

Supplementary Note 2.3 Mitochondrial genome assemblies and annotations 16

Supplementary Figure 2.3 *C. nodosa* mitochondrial chromosomes according to genome recombination activity. 17

2.4 Nuclear-mitochondria and nuclear-chloroplast transfer 18

Supplementary Note 2.4 Nuclear-mitochondria (NUMTs) and nuclear-chloroplast (NUPTs) integrants. 18

Supplementary Table 2.4 NUMTs and NUPTs. 18

3. GENOME ANNOTATION 19

3.1 Non-protein coding RNA annotations 19

Supplementary Note 3.1 rRNA, tRNA and snoRNAs 19

Supplementary Table 3.1 Number of loci of major families of non-protein coding RNAs detected in seagrasses. 20

Supplementary Figure 3.1 Organization of 18S, 5.8S and 28S rRNA repeat units and in the clusters on seagrasses chromosomes. 21

3.2 Transcriptome libraries, sequencing, and assembly 22

Supplementary Note 3.2 Transcriptome libraries 22

Supplementary Table 3.2.1 Transcriptome sequencing data for *Thalassia testudinum* 22

Supplementary Table 3.2.2 Transcriptome sequencing data for *Posidonia oceanica* 23

Supplementary Table 3.2.3 Transcriptome sequencing data for *Cymodocea nodosa* 23

Supplementary Table 3.2.4 Transcriptome sequencing data for *Potamogeton acutifolius.* 24

4. GENOME EVOLUTION 25

4.1 Transposable elements 25

Supplementary Table 4.1 Statistics on transposable elements (TE). 25

Supplementary Figure 4.1 Insertion time distributions of LTR/Gypsy and LTR/Copia in *T. testudinum*, *P. oceanica*, *C. nodosa* and *Z. marina*. Note differences in the scale of the y-axis. See main text for details. 25

4.2 Identifying Whole Genome Duplications (WGD) 26

Supplementary Note 4.2.1 K_S age distributions 26

Supplementary Figure 4.2.1 K_S distributions for anchor pair duplicates (duplicates laying in duplicated, colinear blocks) and the whole paranome of four seagrasses, as well as for *P. acutifolius* and *S. polyrhiza*, generated by the wgd software (see Methods). 27

Supplementary Figure 4.2.2 Comparison of *Z. marina*, *P. oceanica*, and *T. testudinum* with a reconstructed ancestral monocot karyotype (AMK) (Murat et al. 2017). 28

Supplementary Figure 4.2.3 Comparison of the ancestral monocot karyotype (AMK) (Murat et al. 2017) with *C. nodosa* and comparison of *P. oceanica* and *C. nodosa*. 29

Supplementary Figure 4.2.4 Comparison of *P. acutifolius* with the ancestral monocot karyotype (AMK) (Murat et al. 2017), *Z. marina, P. oceanica,* and *C. nodosa*, respectively. 30

Supplementary Figure 4.2.5 K_S Distributions for paralogs and the whole paranome of four seagrasses and *P. acutifolius* generated by KSRATES software. 31

Supplementary Note 4.2.2 Gene tree-species tree reconciliation 32

Supplementary Figure 4.2.6 Bayesian inference of retention rates (q) of 11 hypothetical WGD models in WHALE (Zwaenepoel and Van de Peer 2019a). 33

Supplementary Note 4.2.3 Absolute dating of WGDs 34

Supplementary Figure 4.2.7 Estimation of the 'absolute age' of the WGT/WGD events in seagrasses and *P. acutifolius* by phylogenomic dating. 35

Supplementary Table 4.2 The absolute of WGD events taken from literature 36

Supplementary Figure 4.2.8 Estimation of the 'absolute age' of seven independent WGD events experienced by *E. guineensis*, *A. officinalis*, *R. apiculata*, *A. marina* and *U. gibba* respectively by phylogenomic dating of corresponding paralogues. 37

4.3 Phylogenetic tree construction and estimation of divergence time 38

Supplementary Note 4.3 Species selection and construction of time-calibrated phylogeny. 38

5. ADAPTATION TO THE MARINE ENVIRONMENT 39

5.1. Use it or lose it 39

Supplementary Figure 5.1 Normalized gene counts for each species 39

5.2 Pathogen resistance (R-) genes 40

Supplementary Note 5.2 Pathogen resistance gene 40

Supplementary Table 5.2 *NLR* gene counts by domain architecture and completeness in seagrasses and *P. acutifolius.* 41

Supplementary Figure 5.2.1 Phylogenetic tree of seagrass *NLR* genes based on NBS domain. 42

Supplementary Figure 5.2.2 Distribution of seagrass *NLR* genes across chromosomes**.** 43

5.3 Heat Shock factor (*HSF*) gene family evolution 43

Supplementary Note 5.3 *HSF* gene family 43

Supplementary Table 5.3 Average (± SD) number of total *HSF*s and number of *HSF*s from the three main classes (*HSFA*, *HSFB* and *HSFC*) in the analyzed plant genomes. 45

5.4 Cellular salt tolerance 46

Supplementary Figure 5.4.1 Sequence alignment showing amino acid substitutions in regulatory domains of *SOS1* orthologs of seagrasses, indicating a diverged but convergent regulation of *SOS1*/*NHX7* in these species. 46

Supplementary Figure 5.4.2 Sequence alignment of *AKT5/6/1* showing the loss of Shaker-type K^+ channels with a TTGYGD-selectivity filter in all seagrasses. 47

5.5 Hypoxia 48

Supplementary Figure 5.5.1 Differential expression of *ERF-VII*s in the four seagrass species. 48

Supplementary Figure 5.5.2 Syntenic relationship of genes mentioned in the main text for *P. oceanica*, *T. testudinum*, *Z. marina*, *C. nodosa*, and *P. acutifolius*. 49

5.6 Light perception, circadian clock, and photosynthetic carbon acquisition 50

Supplementary Note 5.6.1 CO_2-concentrating mechanisms (CCMs) and photosynthetic carbon acquisition 50

Supplementary Table 5.6 Prediction of sub-cellular localizations of α-Carbonic Anhydrases (*α-CA*) in the studied seagrass species and *P. acutifolius*. 51

52

Supplementary Figure 5.6.1 Differential expression of *α-CA*, *β-CA* and *λ-CA* in root, rhizome, flower, and leaf tissues of the studied seagrass species. 52

Supplementary Note 5.6.2 Photosynthesis 53

Supplementary Figure 5.6.2 Gene families containing photosystems I and II components and Light-harvesting chlorophyll protein complex. 54

Supplementary Figure 5.6.3 Differential expression of Cyt b6/f complex, *LHCB* and electron transport genes in *Z. marina* and *C. nodosa*. 55

Supplementary Note 5.6.3 Light Signaling & Circadian Clock 55

Supplementary Figure 5.6.4 Gene families containing photoreceptors and the main integration of light signalling toolkit genes. 57

Supplementary Figure 5.6.5 The N terminus alignment of UVB-Resistance 8. 58

Supplementary Figure 5.6.6 Phylogenetic tree of phytochromes obtained from the 84 proteins sequences included in the orthogroups OG0007303, OG0003273, OG0011336 and OG0026441. 59

5.7 NAC transcriptional factors 60

Supplementary Note 5.7 NAC transcriptional factors 60

Supplementary Figure 5.7 Evolutionary analysis by Maximum Likelihood method of *JUB1* in seagrasses. 60

5.8 Nitrogen metabolism 61

Supplementary Note 5.8 Nitrogen metabolism 61

5.9 Flower and pollen development 61

Supplementary Note 5.9 Flower and pollen development 61

Supplementary Table 5.9. The MADS-box genes in seagrasses and *P. acutifolius* 62

Supplementary Figure 5.9 Normalized gene copy numbers for flower and pollen development genes and gene families for 96 species, including 6 genomic data and 90 transcriptomic data 63

1. Sampling species, metadata, and DNA and RNA preparation

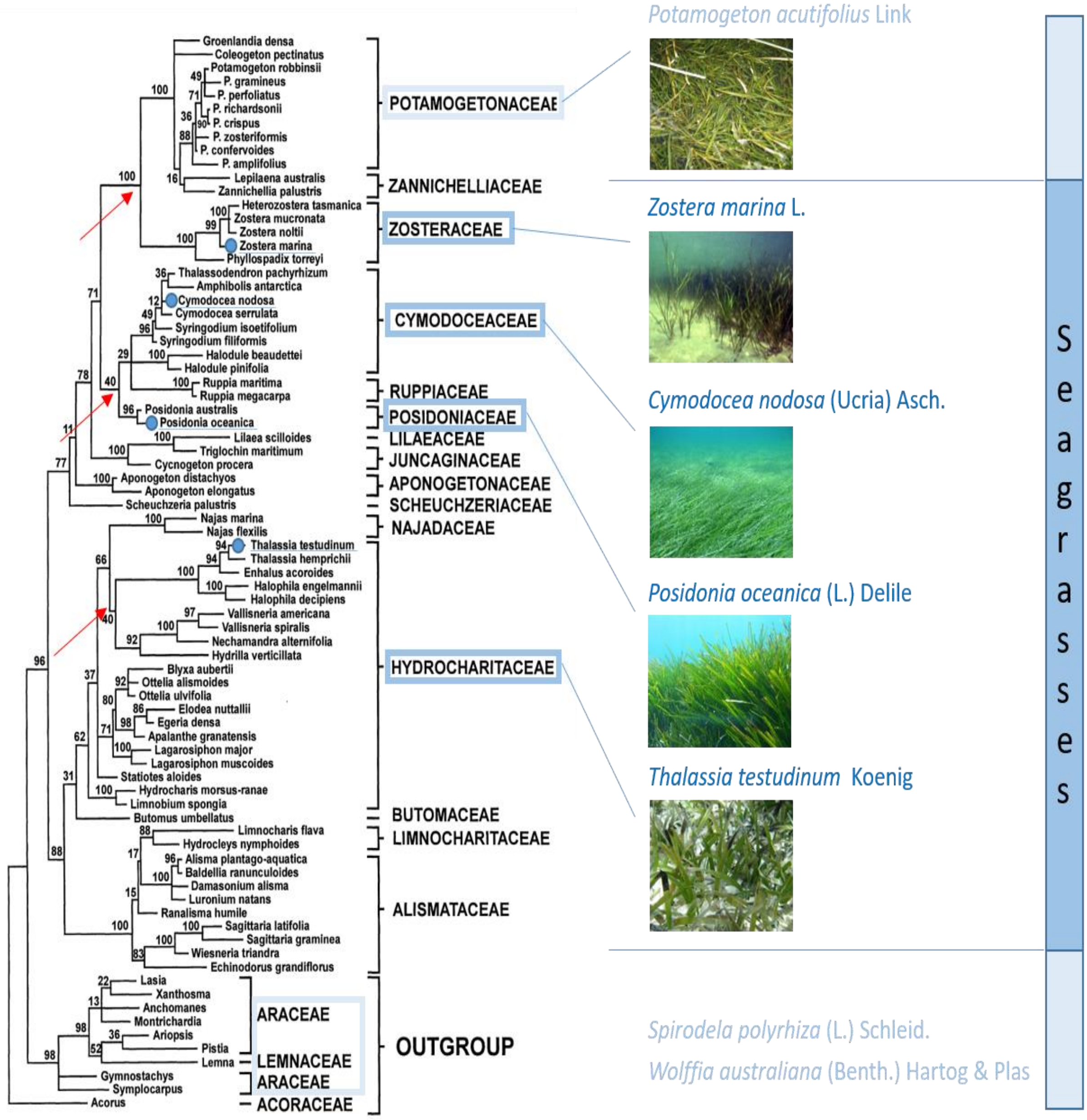

Supplementary Figure 1.1 Phylogenetic tree of Alismatales, including seagrasses.

The tree is based on a Maximum parsimony analysis of *rbcL* genes, adapted from (Les et al. 1997). The four seagrass species discussed in the present study are marked by blue dots. The one freshwater species sequenced in the present study, *Potamogeton acutifolius*, is missing from the tree, but close relatives are included (sister group of Zosteraceae). Solid red arrows indicate nodes denoting the aquatic-marine splits.

Supplementary Table 1.1 Plant material metadata.

See Materials and Methods for details on DNA and RNA preparation. Additional information for *Zostera marina* can be found in (Olsen et al. 2016) and (Ma et al. 2021a).

**Species**	**Code**	**Location**	**Date**	**Collectors**	**Depth**	**Lat**	**Long**	**Tissue**	**HMW DNA extraction method /RNA extraction method**
Thalassia testunidum<br><br>Diploid 2N=18	Tt-101	Coconut Grove Dog Park, Crocodile Point, Miami, FL USA	12 June 2019	JL Olsen & JE Campbell	-1 m	40.78735	14.08974	Leaf<br><br>Leaf Rhizome	CTAB via Arizona Genomics Institute.<br><br>/NucleoSpin RNA Plant and Fungi Kit (Machenery Nagel, USA)
Posidonia oceanica<br><br>Diploid 2N=20	Po-2	Gulf of Pozzuoli, Napoli, Italy	15 May 2019	G Procaccini	-7 m	40.78735	14.08974	Leaf<br><br>Leaf Rhizome Root	As above<br><br>As above
Cymodocea nodosa<br><br>Diploid 2N=18 Known polyploidy in some populations	Cn-1	Miseno Cape, Napoli, Italy	15 May 2019	G Procaccini & L Marin Guirao	-10 m	40.78396	14.07458	Leaf<br><br>Leaf Rhizome Root Flowers	As above<br><br>As above
Potamogeton acutifolius<br><br>Diploid 2N=26 Known polyploidy in other species<br><br>Freshwater	Pa	Baláta-lake, Hungary, Somogy county, near Kaszó	18 Sept 2020	A. Mesterházy	-1.2 m	14.31388	17.205	Leaf<br><br>Leaf Turion Root	Max Planck-Genome with a NucleoBond HMW DNA kit (Macherey Nagel).<br><br>RNAeasy Plant Kit (Qiagen)

2. Genome sequencing and assemblies

2.1 Nuclear genomes

Supplementary Table 2.1.1 Genomic libraries included in each seagrass genome assembly and their respective assembled sequence coverage levels in the final release.

	Library	Sequencing Platform	Average Read/Insert Size	Read Number	Assembled Sequence Coverage (x)
Thalassia testudinium	JDRM	Illumina	400	2,454,318,746	92.04
	IYTB	Illumina/HiC	N/A	1,829,959,374	68.62
	Total Illumina			4,284,278,120	161
		PacBio HiFi	18,712*	11,884,855	51.53
Posidonia oceanica	JDRD	Illumina	400	2,700,014,314	135
	JDMC	Illumina/HiC	N/A	3,843,643,266	192.1
	Total Illumina			6,543,657,580	327
		PacBio HiFi	19,006*	12,018,008	79.44
Cymodocea nodosa	JLSQ	Illumina	400	397,732,894	119.3
	JHNL	Illumina/HiC	N/A	296,170,716	88.9
	Total Illumina			693,903,610	208
		PacBio HiFi	23,472*	1,698,568	79.24
Potamogeton acutifolius	N/A	Illumina/Tell-Seq	N/A	54,401,190	13.4
	N/A	PacBio HiFi	14,000*	1,900,000	43.5

*Average read length of PacBio reads

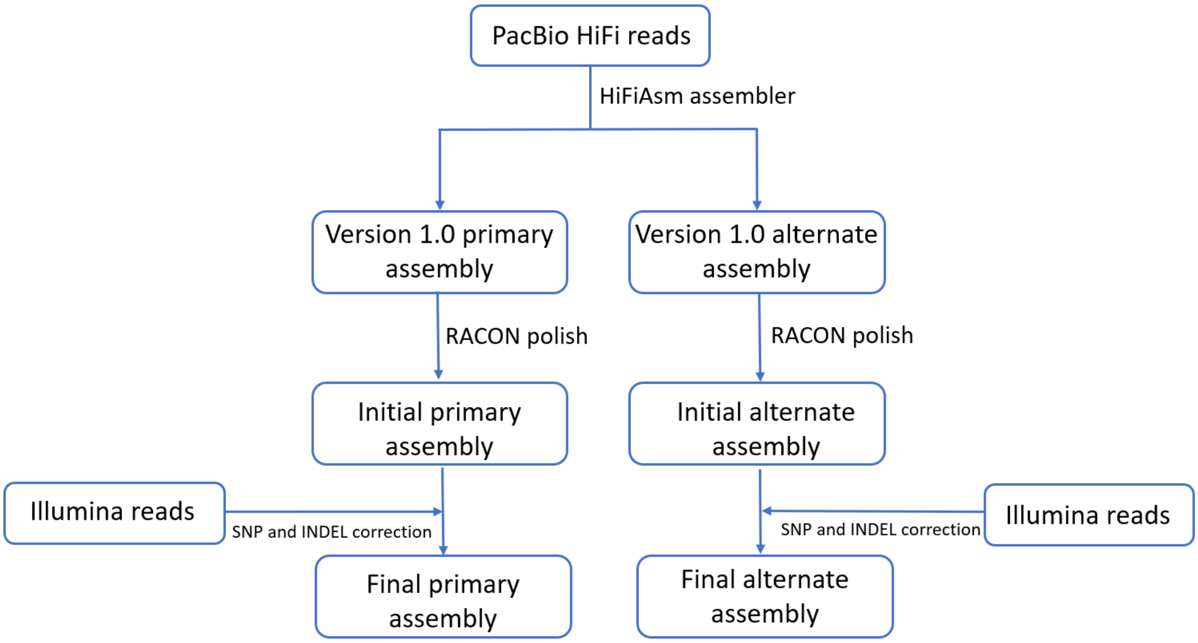

Supplementary Figure 2.1.1 Genome assembly pipeline used for *T. testudinum*, *P. oceanica* and *C. nodosa*

Supplementary Table 2.1.2 Summary statistics of the initial output of the primary RACON polished HiFiAsm assembly.

The table shows number of contigs and total numbers of assembled base pairs for each set of scaffolds greater than the size listed in the left-hand column.

	Minimum Scaffold Length	Number of Scaffolds	Number of Contigs	Scaffold Size	Basepairs	% Non-gap Basepairs
Thalassia testudinium	5 Mb	14	14	4,757,383,018	4,757,383,018	100.00%
	2.5 Mb	14	14	4,757,383,018	4,757,383,018	100.00%
	1 Mb	17	17	4,761,201,586	4,761,201,586	100.00%
	500 Kb	21	21	4,764,120,924	4,764,120,924	100.00%
	250 Kb	29	29	4,766,780,278	4,766,780,278	100.00%
	100 Kb	152	152	4,784,190,954	4,784,190,954	100.00%
	50 Kb	609	609	4,813,980,144	4,813,980,144	100.00%
	25 Kb	1,965	1,965	4,865,616,055	4,865,616,055	100.00%
	10 Kb	1,987	1,987	4,866,121,113	4,866,121,113	100.00%
	5 Kb	1,987	1,987	4,866,121,113	4,866,121,113	100.00%
	2.5 Kb	1,987	1,987	4,866,121,113	4,866,121,113	100.00%
	1 Kb	1,987	1,987	4,866,121,113	4,866,121,113	100.00%
	0 bp	1,987	1,987	4,866,121,113	4,866,121,113	100.00%
Posidonia oceanica	5 Mb	15	15	3,003,306,251	3,003,306,251	100.00%
	2.5 Mb	15	15	3,003,306,251	3,003,306,251	100.00%
	1 Mb	21	21	3,012,259,886	3,012,259,886	100.00%
	500 Kb	29	29	3,017,253,515	3,017,253,515	100.00%
	250 Kb	52	52	3,024,690,979	3,024,690,979	100.00%
	100 Kb	201	201	3,045,962,177	3,045,962,177	100.00%
	50 Kb	1,061	1,061	3,100,982,328	3,100,982,328	100.00%
	25 Kb	3,383	3,383	3,190,011,804	3,190,011,804	100.00%
	10 Kb	3,468	3,468	3,191,950,648	3,191,950,648	100.00%
	5 Kb	3,468	3,468	3,191,950,648	3,191,950,648	100.00%
	2.5 Kb	3,468	3,468	3,191,950,648	3,191,950,648	100.00%
	1 Kb	3,470	3,470	3,191,952,950	3,191,952,950	100.00%
	0 bp	3,470	3,470	3,191,952,950	3,191,952,950	100.00%
Cymodocea nodosa	5 Mb	21	21	370,838,187	370,838,187	100.00%
	2.5 Mb	22	22	375,803,086	375,803,086	100.00%
	1 Mb	26	26	381,781,051	381,781,051	100.00%
	500 Kb	27	27	382,524,700	382,524,700	100.00%
	250 Kb	46	46	388,996,552	388,996,552	100.00%
	100 Kb	148	148	403,137,124	403,137,124	100.00%
	50 Kb	678	678	437,596,903	437,596,903	100.00%
	25 Kb	1,362	1,362	465,959,990	465,959,990	100.00%
	10 Kb	1,362	1,362	465,959,990	465,959,990	100.00%
	5 Kb	1,362	1,362	465,959,990	465,959,990	100.00%
	2.5 Kb	1,362	1,362	465,959,990	465,959,990	100.00%
	1 Kb	1,362	1,362	465,959,990	465,959,990	100.00%
	0 bp	1,362	1,362	465,959,990	465,959,990	100.00%

Supplementary Note 2.1 Final Primary assemblies for main and alternate haplotypes.

The final primary assembly (see Methods) of *T. testudinum* contains 4,261.9 Mb of sequence, consisting of 184 contigs with a contig N50 of 371.6 Mb and a total of 98.92% of assembled bases in 9 chromosomes. The final primary assembly of *P. oceanica* contains 2,963.0 Mb of sequence, consisting of 19 contigs with a contig N50 of 355.8 Mb and a total of 99.98% of assembled bases in 10 chromosomes. The final primary assembly of *C. nodosa* contains 379.5 Mb of sequence, consisting of 22 contigs with a contig N50 of 21.9 Mb and a total of 100% of assembled bases in 18 chromosomes (Supplementary Table 2.1.3).

Correspondingly, the final alternative release of *T. testudinum* contains 4,177.2 Mb of sequence, consisting of 3,364 contigs with a contig N50 of 6.1 Mb and a total of 85.18% of assembled bases in 9 chromosomes. The final alternative release of *P. oceanica* contains 2,496.3 Mb of sequence, consisting of 826 contigs with a contig N50 of 8.7 Mb and a total of 99.67% of assembled bases in 10 chromosomes. The final alternative assembly for *C. nodosa* contains 374.7 Mb of sequence, consisting of 28 contigs with a contig N50 of 20.8 Mb and a total of 99.91% of assembled bases in 18 chromosomes (Supplementary Table 2.1.3). The final assembly of *P. acutifolius* is in the Table 1.

Supplementary Table 2.1.3 Final summary primary and alternate assembly statistics for each chromosome-scale assembly (see for further details on *Zostera marina* v3.1).

Final Primary Assembly Release Haplotype 1	genome release stats:	*Thalassia testudinium*	*Posidonia oceanica*	*Cymodocea nodosa*	*Zostera marina v3.1*
	Number of chromosomes (1N)	9	10	18	6
	Number of main genome scaffold total:	169	13	18	310
	Number of main genome contig total:	184	19	22	432
	Length of main genome scaffold sequence total: (MB)	4262.1	2963.1	379.6	260.5
	Length of main genome contig sequence total:	4261.9	2963	379.5	259.3
	Main genome scaffold L/N50: (MB)	4/523.9	4/355.8	8/22.6	4/34.6
	Main genome contig L/N50: (MB)	5/371.6	4/355.8	8/21.9	12/7.0
	Number of scaffolds > 50 KB:	49	13	18	217
	% main genome in scaffolds > 50 KB:	99.9	100	100	98.9
Final Alternative Assembly Release Haplotype 2	Number of chromosomes (1N)	9	10	18	N/A
	Number of alternate genome scaffold total:	2264	29	22	N/A
	Number of alternate genome contig total:	3364	826	28	N/A
	Length of alternate genome scaffold sequence total: (MB)	4188.2	2504.3	374.8	N/A
	Length of alternate genome contig sequence total: (MB)	4177.2	2496.3	374.7	N/A
	Genome scaffold L/N50: (MB)	5 / 458.8 Mb	3/352.0	8/22.1	N/A
	Genome contig L/N50: (MB)	162 / 6.1 Mb	85/8.7	8/20.8	N/A
	Number of scaffolds > 50 KB	805	29	22	N/A
	% main genome in scaffolds > 50 KB	98.7	100	100	N/A

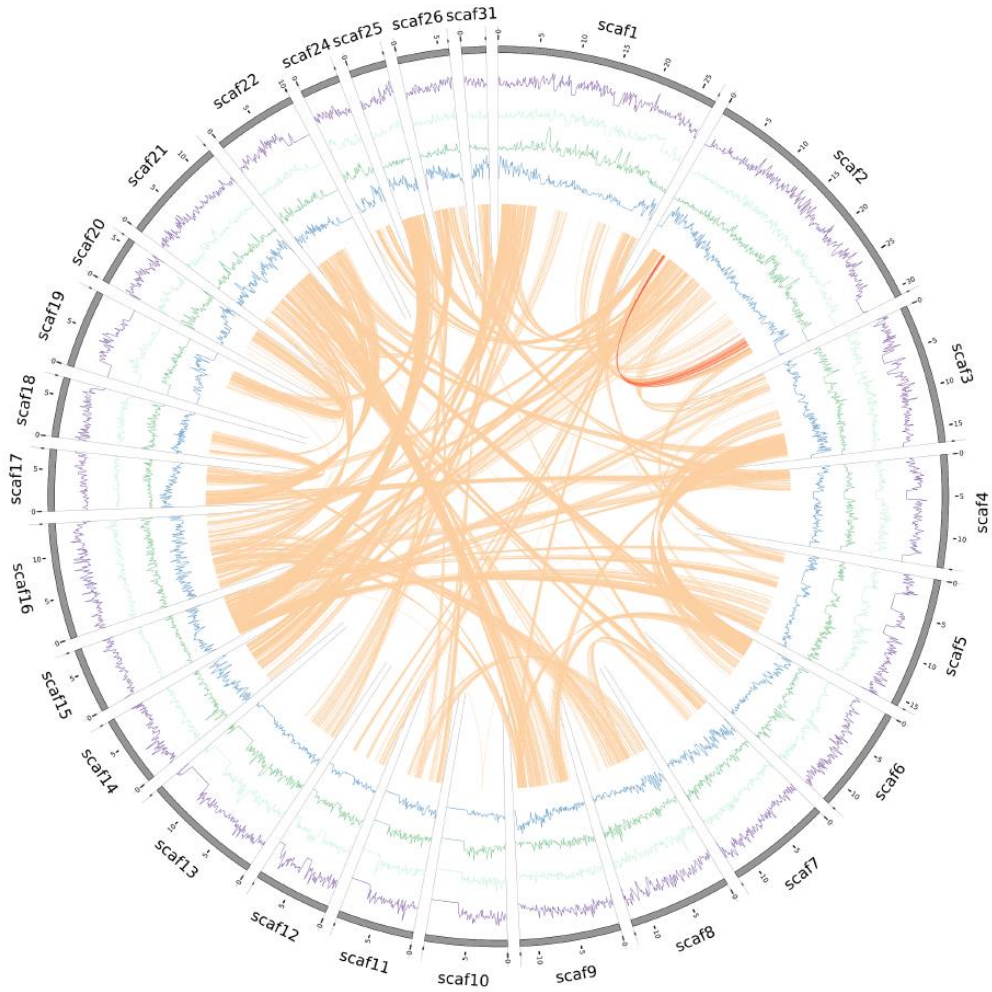

Supplementary Figure 2.1.2 Distribution of the genomic features for the 26 largest scaffolds of *P. acutifolius*.

Tracks from the inner to outer side correspond to gene density (blue); LTR/Gypsy density (green); LTR/Copia (orange); DNA transposable elements (pink) and chromosomes (with length in Mb). Curved lines through the center denote synteny between different scaffolds.

Supplementary Table 2.1.4. Primary genome assembly, annotation statistics and BUSCO completeness assessment of protein coding sequences. See Supplementary Table 2.1.3 for additional details for the alternate haplotypes.

Statistics	*T. testudinum*	*P. oceanica*	*C. nodosa*	*Z. marina v 3.1 (Ma et al. 2021b)*	*P. acutifolius*
Assembly					
Haploid-chromosome number	9	10	18	6	13
Genome size, Mb	4261.9	2963.0	379.5	260.5	612
Contig N50, (Mb)	371.6	355.8	21.9	7.0	3.09
Scaffold N50 (Mb)	523.9	355.8	22.6	34.6	4.45
Genome assembly BUSCO					
Complete %	73.3	93.6	92.2	93.4	96.9
single-copy %	72.1	92.2	90.8	90.9	93.7
duplicated %	1.2	1.4	1.4	2.5	3.2
Fragmented %	6.9	2.7	2.7	1.1	1.1
Missing %	19.8	3.7	5.1	5.5	2.0
Annotation					
Protein coding genes	25,665	23,306	20,563	22,256	21,277
Mean gene length, bp	19,151	7,017	5,866	3,237	3,448
Mean CDS length, bp	1,077	1,210	1,207	1,241	1,290
Mean exon length, bp	218	222	214	248	243
Mean exon per gene	4.95	5.46	5.65	5.01	5.3
Mean intron length, bp	4,576	1,303	1,003	499	503
Number of introns > 1 kb %	36.2	24.2	22.5	9.5	9.7
Number of introns > 10 kb %	13.5	2.6	1.4	0.6	0.3
Number of introns > 20 kb %	7.1	0.7	0.2	0.06	0.01
Longest intron, bp	283,604	224,280	89,280	46,497	34,817
Transcriptome support					
TPM > 0 %	87.4	84.7	97.1	91.5	82.7
TPM > 1 %	72.9	70.1	86.6	80.2	75.6
BUSCO					
Complete %	94.2	97.4	95.8	95.7	97.5
single-copy %	92.1	95.2	94.4	93.2	94.6
duplicated %	2.1	2.2	1.4	2.5	2.9
Fragmented %	1.1	0.2	0.3	80.5	0.2
Missing %	4.7	2.4	3.9	3.8	2.3
Functional annotation					
	92.3%	96.8%	91.8%	96.2%	98.9%

2.2 Chloroplast genomes

Supplementary Note 2.2 Chloroplast genome assemblies and annotations

Complete chloroplast genomes were assembled *de novo* from Illumina short read data with NOVOPlasty using the *rbcL* gene as a seed (Dierckxsens et al. 2017). *Thalassia testudinum* cpDNA was additionally, manually curated according to the chloroplast derived PacBio contigs from the main genome assembly. The *Z. marina* genome was obtained from (Ma et al. 2021a). All chloroplast genomes were polished with pilon (Walker et al. 2014) in addition to manual curation. The gene content of the chloroplast genomes was annotated using the software GeSeq (Tillich et al. 2017) and with Chloë (Zhong 2020). As the preferred annotator for CDS and rRNA, ARAGORN (Laslett and Canback 2004) was used for tRNA annotation. Genes that were much shorter than expected are marked as fragmented. Figures were drawn with Chloroplot (Zheng et al. 2020).

We identified 79, 78, 75, and 74 protein-coding genes (only counting genes in the inverted repeats once) in the *C. nodosa*, *Z. marina*, *T. testudinum*, and *P. oceanica* chloroplast genomes, respectively, of which *Z. marina* lost one gene (*rps19*), *T. testudinum* lost four genes (*accD, infA, ndhB,* and *ndhF*) and *P. oceanica* lost five genes (*ndhG, ndhH, ndhI, ndhJ,* and *ndhK*) (Supplementary Figure 2.2.1 – Supplementary Figure 2.2.4). We note that the chloroplast NADH dehydrogenase complex, encoded by *ndh* genes, has been lost in *P. oceanica* and *T. testudinum*. The loss has been proposed to have happened independently in various Alismatales lineages, correlating with the submerged fresh or marine habitat (Ross et al. 2016). Notably, the complex is lost in the two species that demonstrate the highest levels of genome perturbation.

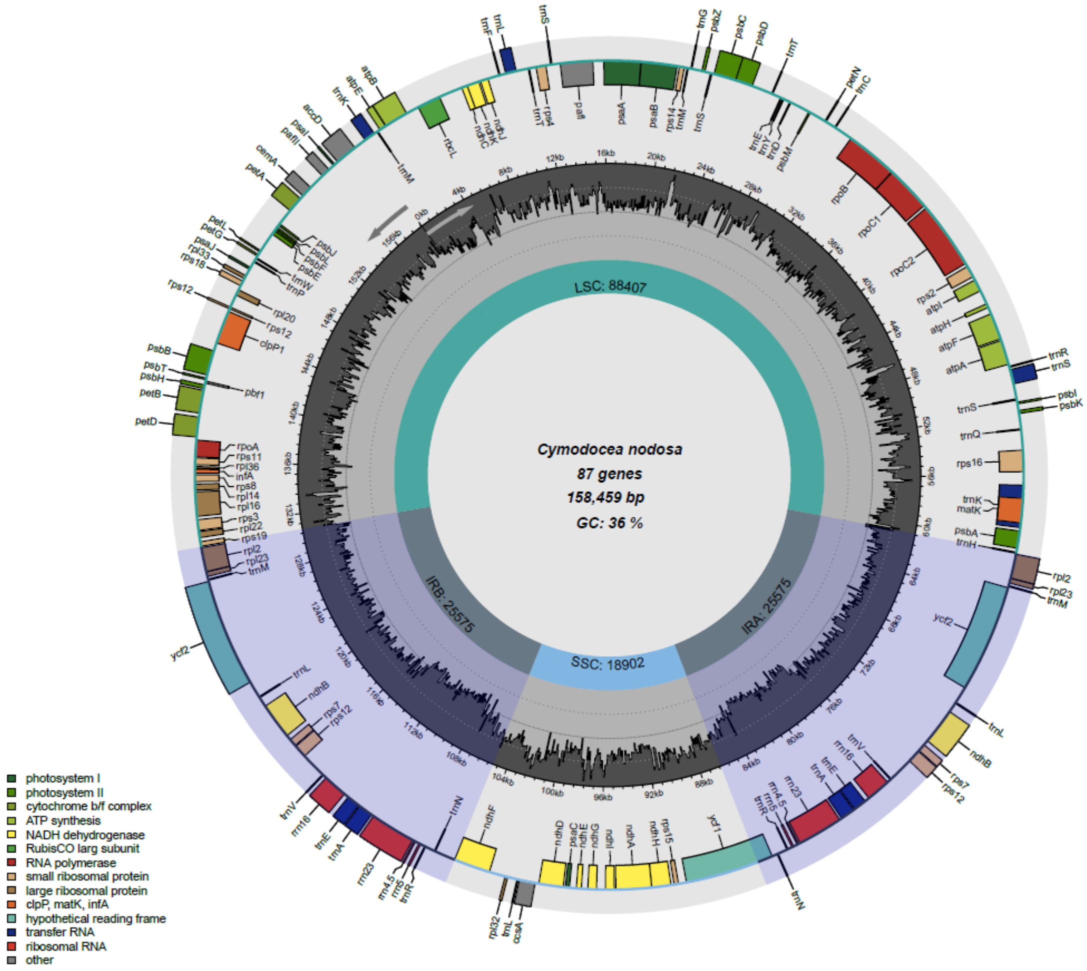

Supplementary Figure 2.2.1 The complete C. nodosa chloroplast genome.

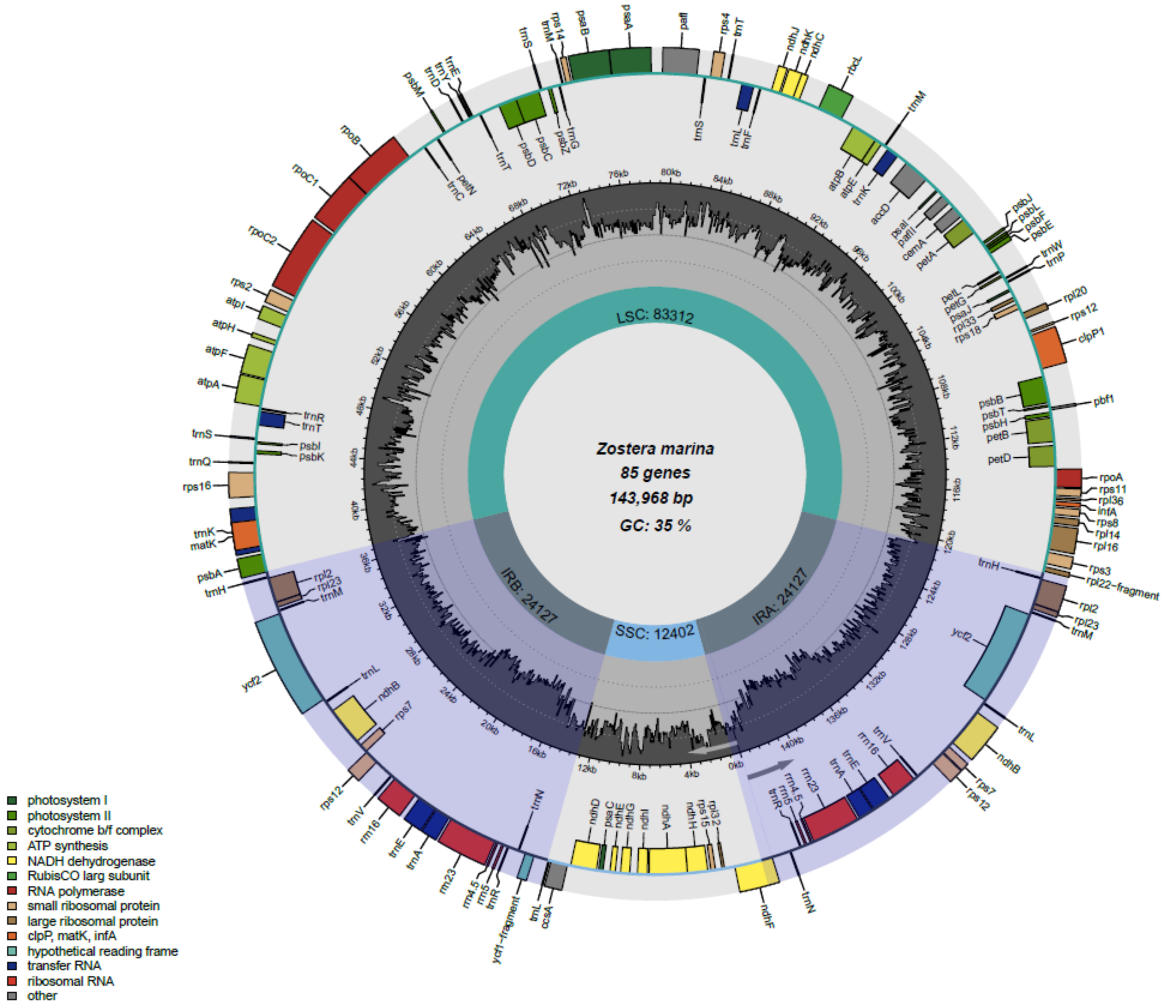

Supplementary Figure 2.2.2 The complete *Z. marina* chloroplast genome.

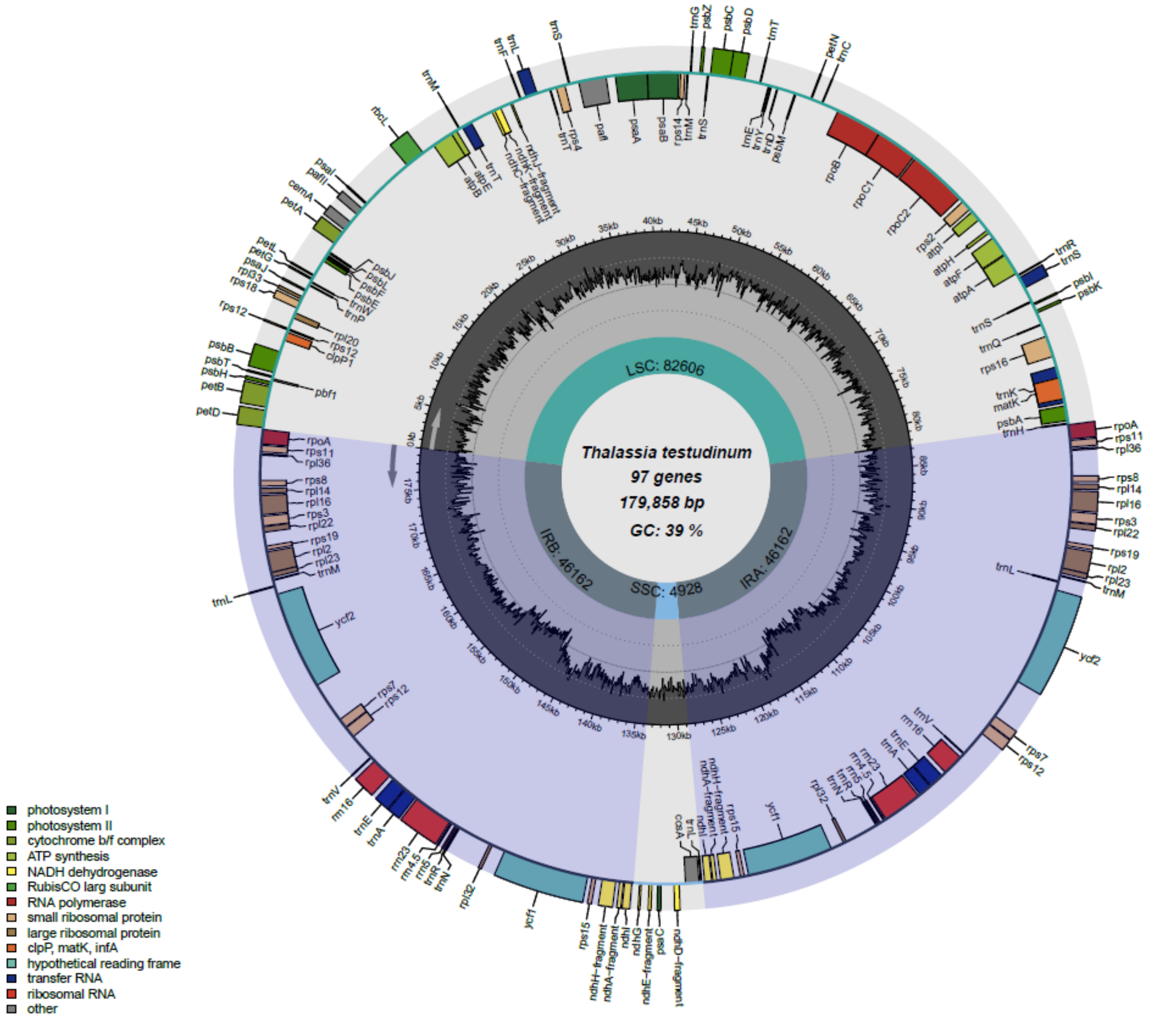

Supplementary Figure 2.2.3 The complete *T. testudinum* chloroplast genome.

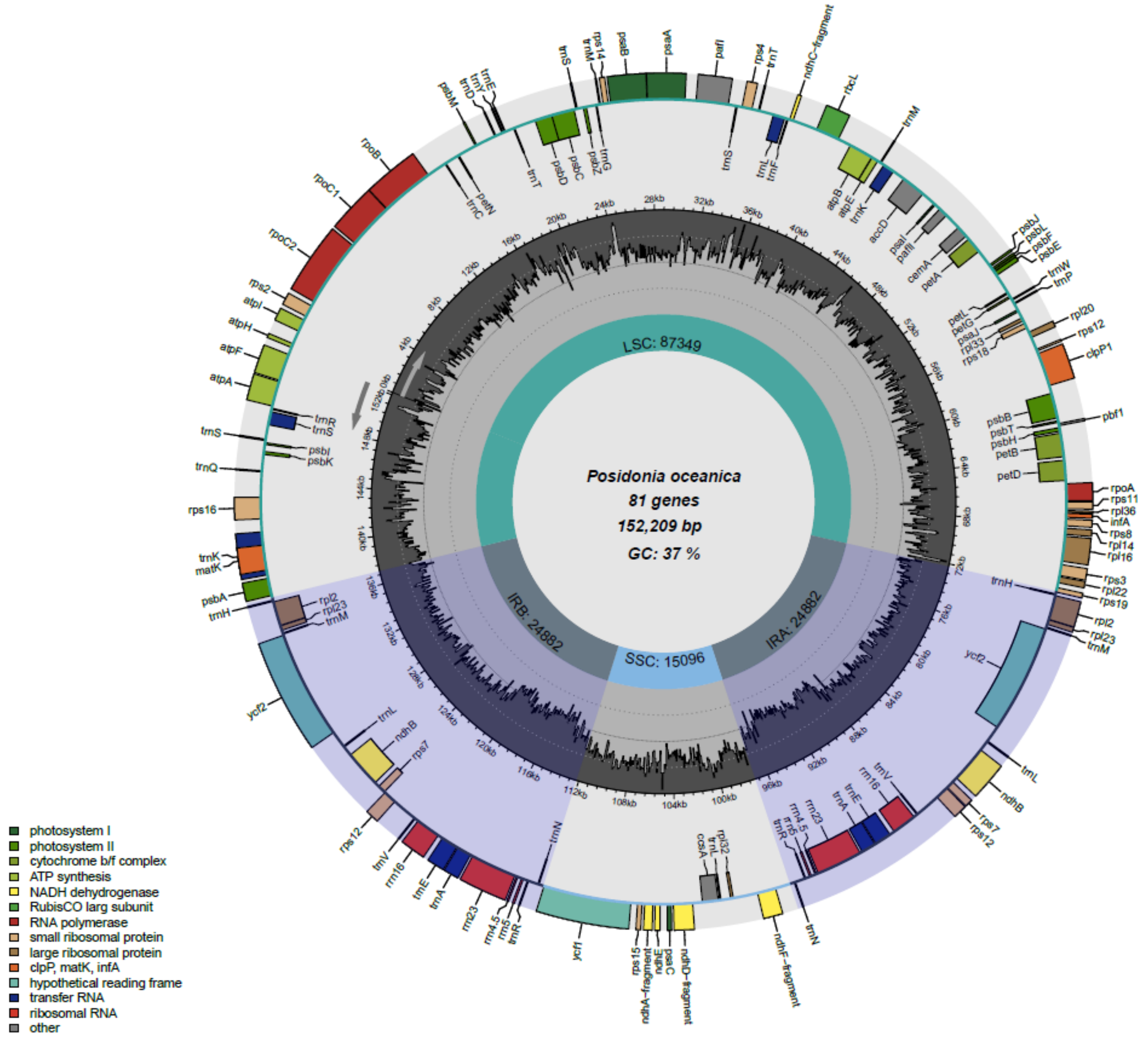

Supplementary Figure 2.2.4 The complete *P. oceanica* chloroplast genome.

2.3 Mitochondrial genomes

Supplementary Note 2.3 Mitochondrial genome assemblies and annotations

Mitochondrial genomes were manually assembled de novo from PacBio contigs containing at least five mitochondrial genes. All mitochondrial genomes were polished with pilon. Genes were identified by BLAST and TBLASTN (Altschul et al. 1990) using the mitochondrial gene collection in (Petersen et al. 2017).

Unlike animal mtDNA, plant mtDNA genomes vary enormously in size and do not exist as a single stable circle but rather as a dynamic combination of linear, branched and small circular loops called isoforms (Morley and Nielsen 2017). The additional DNA is dominated by repeats and noncoding regions. The various isoforms are due to recombination (Kozik et al. 2019). Therefore, the classic circular representation is inappropriate. Accordingly, we found varying degrees of genome size, completeness, and fragmentation in the seagrass mitochondrial genomes.

The *Z. marina* mitochondrial genome is complete (Marina et al. 2023) as is *C. nodosa*. *T. testudinum* is incomplete and only a single mt-contig was recovered for *P. oceanica*.

The complete assembly of the *C. nodosa* mitochondrial genome resulted in one circular chromosome of 293,083 bp with two recombinationally active direct repeats of 2,683 bp and 2,476 bp. Given the possible recombination events, the genome has six chromosomes (Supplementary Figure 2.3) All PacBio assembly contigs containing fragments of five or more mitochondrial genes agree with at least one of the six chromosomes. In total, 36 of 44 mitochondrial protein and rRNA coding genes (or their fragments), were detected by BLAST in the assembled genome, thus providing additional evidence of completeness.

The incomplete assembly of the *T. testudinum* mitochondrial genome (not shown) comprises two chromosomes with no shared DNA between them: one circular of 192,371 bp and the other one of 136,585 bp has an open loop shape. The open loop shape of the second chromosome is formed by an inverted repeat of 10,932 bp. At least one additional scaffold of 90,412 bp is likely to have originated from one of the chromosomes although the exact configuration is unclear; the scaffold has an open loop shape and overlaps with the 192,371 bp circle. The two chromosomes were further used in the analysis. In total 30 of 44 mitochondrial protein and rRNA coding genes (or their fragments) are encoded in the two chromosomes. However, a few additional genes detected in other small scaffolds and in nuclear chromosomes strongly suggests that the assembly status of *T. testudinum* mitochondrial genome remains incomplete.

Only a single mitochondrial contig (132,748 bp) was found for *P. oceanica* and was used for further analysis (not shown). This contig contains 20 of 44 mitochondrial protein and rRNA coding genes (or their fragments) confirming the fragmented status of the assembly. Additional mitochondrial genes were detected on nuclear chromosomes.

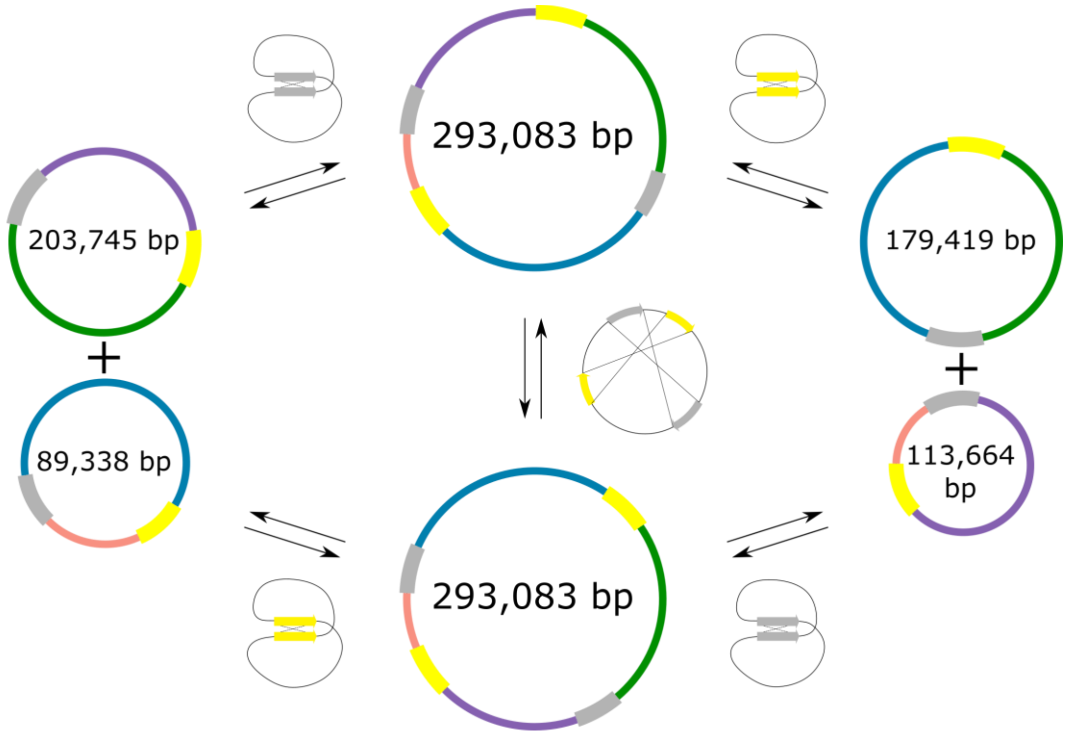

Supplementary Figure 2.3 *C. nodosa* mitochondrial chromosomes according to genome recombination activity.

See text for explanation and discussion. The two yellow and two grey segments represent two pairs of 2,683 bp and 2,476 bp respectively. Other colored segments represent parts of the mitogenome that remain stable during recombination. The two smaller circles (a.k.a subgenomes) are formed when one repeat pair recombines, while the mirrored conformation is the result of recombination via both repeat pairs simultaneously.

2.4 Nuclear-mitochondria and nuclear-chloroplast transfer

Supplementary Note 2.4 Nuclear-mitochondria (NUMTs) and nuclear-chloroplast (NUPTs) integrants.
Nuclear-mitochondria (NUMTs) and nuclear-chloroplast (NUPTs) integrants were identified by BLAST using the parameters suggested in (Smith et al. 2011). Shared-overlapped regions in nuclear chromosomes were joined into "joined NUMTs" and "joined NUPTs". Because of the repeats in organellar genomes and non-linear organellar genome assemblies, the same chromosome region might appear multiple time in the BLAST output. Therefore, we joined overlapping blast hits into one and called them "joined NUMTs" and "joined NUPTs". This way we ensure that we do not overestimate the number (and total length) of the NUMTs and NUPTs in the nuclear chromosomes.

The integrated organellar DNAs within the nuclear genome have been shown to play important roles. Several processes can enhance DNA transfer including biotic and abiotic stress, increased organelle copy number or large gene-free regions composed of multiple repeats (Zhao et al. 2019; Ma et al. 2020; Zhang et al. 2020).

We assessed the intensity of intracellular DNA transfer of shared DNA segments between the nucleus and mitochondria (NUMTs), and the nucleus and chloroplasts (NUPTs). *Thalassia testudinum* revealed large uninterrupted NUMTs that point towards relatively recent mitochondrial-DNA transfer, whereas *Z. marina* has very few (Supplementary Table 2.4). The recent LTR/Gypsy burst in *T. testudinum* (Supplementary Figure 4.1) is probably the main cause for *T. testudinum*' s extreme genome size and intron expansion. It is further correlated with increased intracellular DNA transfer from organellar to nuclear genome. Transposable elements have been proposed to contribute to the post-insertion dynamics of the transferred DNA rather than the insertion rate (Michalovova et al. 2013). Thus, multiple insertions of organellar DNA may be another consequence of a general genome instability caused by TE expansion.

Supplementary Table 2.4 NUMTs and NUPTs.
Intracellular DNA transfer and the relative age of shared DNA segments between the nucleus and mitochondria (NUMTs), and the nucleus and chloroplasts (NUPTs). Recent insertions are longer (e.g., *T. testudinum*). Over time, segments become shorter (e.g., *Z. marina*).

**Species**	Mitogenome status	Total length	Max joined NUMT length	Mean joined NUMT length	Joined NUMT >10000bp	Percent shared positions with nucleus
Cymodocea nodosa	complete	292,353	71,177	796	3	59
Posidonia oceanica	fragment	132,748	49,857	880	12	99
Thalassia testudinum	incomplete	328,953	95,806	1,275	108	100
Zostera marina v3.1	complete	187,048	9,623	391	0	26
**Species**	Chloroplast genome status	Total length	Max joined NUPT length	Mean joined NUPT length	Joined NUPT >3000bp	Percent shared positions with nucleus
Cymodocea nodosa	complete	158,459	11,621	387	18	94
Posidonia oceanica	complete	152,209	30,959	289	29	99
Thalassia testudinum	complete	180,136	129,692	527	181	100
Zostera marina v3.1	complete	143,968	6,039	396	1	44

3. Genome annotation

3.1 Non-protein coding RNA annotations

Supplementary Note 3.1 rRNA, tRNA and snoRNAs

The prediction of non-protein coding RNA families (i.e., rRNAs, tRNA and snoRNAs) in *Z. marina*, *C. nodosa*, *P. oceanica*, *T. testudinum* and *P. acutifolius* highlighted an overall expansion in the number of loci in *T. testudinum* when compared to other species (Supplementary Table 3.1). The 5S rRNA (119 nucleotides in length), the minor component of the large subunit of the ribosome, was detected in 10,923 distinct loci in the *T. testudinum* genome, and in 3,566 loci in *P. acutifolius*, 2,605 in *C. nodosa*, 730 in *P. oceanica*, and 364 in *Z. marina*. Clusters of the repetitive units of the large and small ribosomal subunits, organized by the 18S followed by the 5.8S and the 25S rRNAs, of 154, 3,401 and 1,851 nucleotides in length, respectively, were found on a single chromosome in *C. nodosa* (chr 18) and *P. oceanica* (chr 6), on three different chromosomes in *T. testudinum* (chr4, chr5 and chr8) and in different scaffolds in *Z. marina* and *P. acutifolius* (Supplementary Table 3.1 and Supplementary Figure 3.1). All the repetitive units show an almost conserved organization and the length of the two internal transcribed sequences (ITSs) within a species is conserved too, even in the repeated loci along the three different chromosomes in *T. testudinum*.

Of note, *C. nodosa* does not show duplications of the represented RNA families, while an evident expansion, also accompanied by the snoRNAs class, is revealed for *T. testudinum* (Supplementary Table 3.1). This expansion appears also evident in *P. acutifolius* scaffolds, although the scaffold nature of this genome did not allow to confirm the real trend. Also transfer RNA (tRNA) sequences, with a length of 71 nt, were more abundant in *T. testudinum* (1,200 hits) and *P. acutifolius* (4,954), compared to *C. nodosa* (228), *P. oceanica* (367), and *P. oceanica* (478) species. snoRNAs, the RNA class that guides chemical modifications of other rRNAs, show a strong overrepresentation in *T. testudinum* (12,846 hits), compared to the other species (224 in *P. acutifolius*, 99 in *C. nodosa*, 238 in *P. oceanica*, 155 in *Z. marina*), although the number of loci detected in the *P. acutifolius* are lower than *P. oceanica* (Supplementary Table 3.1). This will require deeper investigations when the *P. acutifolius* chromosomes will be defined.

Supplementary Table 3.1 Number of loci of major families of non-protein coding RNAs detected in seagrasses.

Species	Genomic element	5S	5.8S	LSU_EUK(28S)	SSU_EUK(18S)	tRNA	snoRNA
Posidonia oceanica	Chr1	3	-	-	-	68	57
	Chr2	3	-	-	-	51	33
	Chr3	2	-	-	-	27	24
	Chr4	-	-	-	-	32	16
	Chr5	18	-	-	1	53	21
	Chr6	-	160	155	153	43	19
	Chr7	2	1	1	-	35	7
	Chr8	1	-	-	-	12	15
	Chr9	2	-	-	-	19	19
	Chr10	699	1	1	-	20	25
	Scaffolds	-	-	-	-	7	2
	TOTAL	730	162	157	154	367	238
Cymodocea nodosa	Chr1	2,195	-	-	1	8	10
	Chr2	17	-	-	-	20	6
	Chr3	21	-	-	-	19	10
	Chr4	20	-	-	-	23	4
	Chr5	15	-	-	-	22	11
	Chr6	18	-	-	-	8	9
	Chr7	17	-	-	-	14	6
	Chr8	19	1	-	-	21	3
	Chr9	17	-	-	-	6	3
	Chr10	13	-	-	-	13	1
	Chr11	16	-	-	-	16	4
	Chr12	17	-	-	-	1	4
	Chr13	146	-	-	-	3	6
	Chr14	23	-	-	-	13	3
	Chr15	16	-	-	-	7	3
	Chr16	15	-	-	-	6	7
	Chr17	13	-	-	-	15	6
	Chr18	7	51	49	49	13	3
	TOTAL	2,605	52	49	50	228	99
Zostera marina	Chr1	6	-	-	1	55	14
	Chr2	5	-	-	1	79	7
	Chr3	2	-	-	-	59	33
	Chr4	5	1	1	-	48	39
	Chr5	4	1	-	-	47	30
	Chr6	1	-	-	-	55	20
	Scaffolds	341	120	90	124	135	12
	TOTAL	364	122	91	126	478	155
Thalassia testudinum	Chr1	27	-	-	1	120	1,619
	Chr2	41	-	-	-	137	1,712
	Chr3	28	-	-	-	94	1,729
	Chr4	33	74	73	73	126	1,553
	Chr5	16	67	61	64	125	1,387
	Chr6	3,435	-	-	3	113	1,459
	Chr7	41	8	5	6	84	1,306
	Chr8	7,294	111	106	107	75	1,189
	Chr9	8	-	-	-	40	885
	Scaffolds	-	-	-	-	286	7
	TOTAL	10,923	260	245	254	1,200	12,846
Potamogeton acutifoliis	Scaffolds	3,566	1,548	1,665	1,693	4,954	224

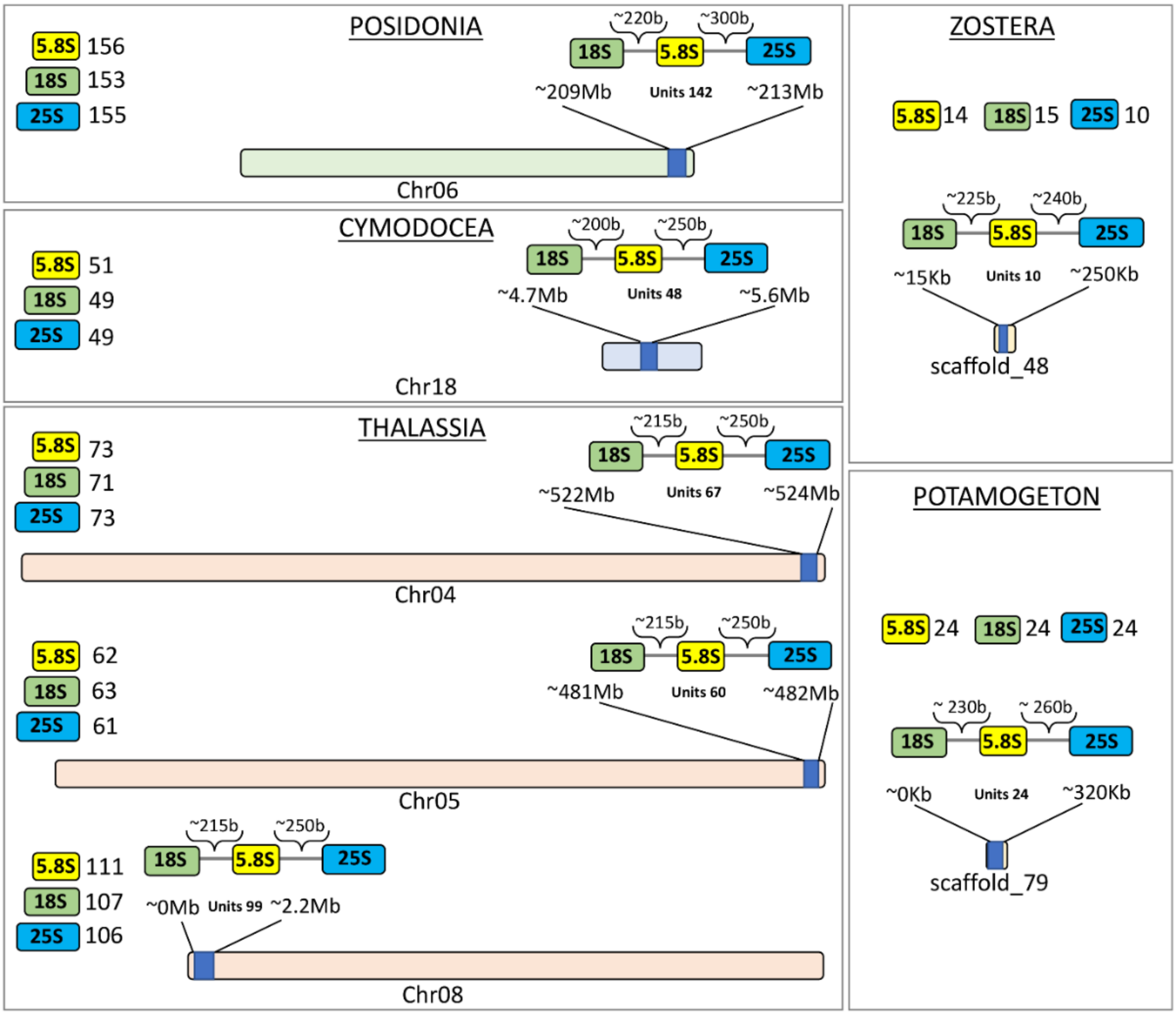

Supplementary Figure 3.1 Organization of 18S, 5.8S and 28S rRNA repeat units and in the clusters on seagrasses chromosomes.

Considering *Zostera marina* and *Potamogeton acutifolius*, the organization of the longest clusters and the scaffold in which they have been found. The length of the ITSs and the number of repeats in a cluster (units number) are reported.

3.2 Transcriptome libraries, sequencing, and assembly

See Methods and Supplementary Table 1.1 for RNA preparation

Supplementary Note 3.2 Transcriptome libraries

Strand-specific RNASeq library(s) were created and quantified by qPCR. RNA sequencing was performed using an Illumina instrument (Supplementary Table 3.2.1 – Supplementary Table 3.2.4). Raw fastq file reads were filtered and trimmed using the JGI QC pipeline resulting in the filtered fastq file. Using BBDuk (https://sourceforge.net/projects/bbmap/), raw reads were evaluated for artifact sequence by kmer matching (kmer=25), allowing 1 mismatch and detected artifact was trimmed from the 3' end of the reads. RNA spike-in reads, PhiX reads and reads containing any Ns were removed. Quality trimming was performed using the phred trimming method set at Q6. Finally, following trimming, reads under the length threshold were removed (minimum length 25 bases or 1/3 of the original read length - whichever is longer).

Supplementary Table 3.2.1 Transcriptome sequencing data for *Thalassia testudinum*

	**Sequencing**	**Tissue**	**library ID**	**Replicate**	**Number of raw Reads**	**Number of clean Reads**	**Number of mapped reads**	**% of aligned reads**
Summary of transcriptome sequencing in *Thalassia*	RNA-seq (NovaSeq S4)	Rhizome	GZGXW	1	121,598,766	118,237,066	114,398,701	96.75
			GZGXX	2	73,636,900	70,598,156	67,892,538	96.17
			GZGXY	3	132,873,546	129,273,100	124,854,697	96.58
		Leaf	GZGXT	1	93,743,754	90,902,870	87,180,663	95.91
			GZGXU	2	62,515,064	61,189,816	58,999,410	96.42
	**Sequencing**	**Tissue**	**library ID**	**Replicate**	**Number of full-length ccs reads**	**Number of high-quality isoforms**	**Number of mapping reads**	**% of aligned reads**
	Iso-seq (SEQUELII)	Leaf	GYNYG	1	3,645,896	217,994	347,411	98.4
			GYNYH	2	3,267,856	135,187		
		Rhizome	GYNYO	1	3,002,957	177,124	351,956	99.1
			GYNYN	2	2,801,968	178,044		

Supplementary Table 3.2.2 Transcriptome sequencing data for *Posidonia oceanica*

	Sequencing	Tissue	library ID	Replicate	Number of raw Reads	Number of clean Reads	Number of mapping reads	% of aligned reads
Summary of transcriptome sequencing in *Posidonia*	RNA-seq (NovaSeq S4)	Leaf	GZGXH	1	118,219,220	110,207,116	106,925,423	97.02
			GYOZB	2	103,334,962	89,188,206	85,643,795	96.03
			GZGXN	3	112,093,828	106,876,636	103,508,597	96.85
		Rhizome	GZGXO	1	90,749,690	87,505,084	84,244,810	96.27
			GZGXP	2	97,902,404	95,308,522	92,137,451	96.67
	**Sequencing**	**Tissue**	**library ID**	**Replicate**	**Number of full-length ccs reads**	**Number of high-quality isoforms**	**Number of mapping reads**	**% of aligned reads**
	Iso-seq (SEQUELII)	Leaf	GZHTC	1	1,852,186	93,731	265,793	99.7
			GZHTB	2	2,542,984	172,871		
		Rhizome	GZHTH	1	2,525,643	183,914	335,745	99.6
			GZHTG	2	1,941,033	152,859		

Supplementary Table 3.2.3 Transcriptome sequencing data for *Cymodocea nodosa*

	Sequencing	Tissue	library ID	Replicate	Number of raw Reads	Number of clean Reads	Number of mapping reads	% of aligned reads
Summary of transcriptome sequencing in *Cymodocea*	RNA-seq (NovaSeq S4)	Leaf	GZGWX	1	229,014,654	221,616,172	215,735,219	97.35
			GZGWY	2	85,687,350	81,851,552	78,999,864	96.52
		Rhizome	GZGWZ	1	152,179,412	142,836,610	138,185,544	96.74
		Root	GZGXA	1	77,449,636	75,252,242	72,717,889	96.63
			GZGXB	2	103,516,046	102,007,494	99,073,508	97.12
		Flower	GZGXC	1	167,193,082	157,226,850	153,290,632	97.50
			GZGXG	2	170,015,134	160,442,432	156,397,982	97.48
	**Sequencing**	**Tissue**	**library ID**	**Replicate**	**Number of full-length ccs reads**	**Number of high-quality isoforms**	**Number of mapping reads**	**% of aligned reads**
	Iso-seq (SEQUELII)	Mixed pool of Rhizome, root and flower	GWWWG	1	1,103,846	130,315	568,012	98.5
			GWWWC	2	2,318,964	171,811		
		Leaf	GZHTN	1	2,102,451	157,082		
			GZHTO	2	1,982,756	117,547		

Supplementary Table 3.2.4 Transcriptome sequencing data for *Potamogeton acutifolius.*

	Sequencing	Tissue	library ID	Replicate	Number of raw reads	Number of clean reads	Number of mapping reads	% of aligned reads
Summary of transcriptome sequencing in *Potamogeton*	RNA-seq (NovaSeq 6000 S4)	Leaf	PALF	1	85,063,302	85,063,302	35,512,097	41.7
		Root	PARO	1	68,366,930	68,366,930	35,807,278	52.4
		Turion	PATU	1	64,107,268	64,107,268	56,978,015	88.9

4. Genome Evolution

4.1 Transposable elements

Supplementary Table 4.1 Statistics on transposable elements (TE).

**TE Statistics**	***T. testudinum***	***P. oceanica***	***C. nodosa***	***Z. marina***	***P. acutifolius***
Overall TE content (%)	87.36	85.18	64.65	65.57	40.76
Overall LTR (%)	72.27	65.89	45.72	41.72	12.41
LTR/Gypsy (%)	63.18	57.8	17.43	32.11	3.39
LTR/Copia (%)	8.28	4.09	28.29	9.49	6.11
LINE (%)	5.06	2.63	2.38	4.05	0.59
SINE (%)	0.03	0.23	0.00	0.09	0.23
DNA transposon (%)	7.78	8.39	2.93	5.71	7.14
Unclassified (%)	2.21	2.76	13.61	14.00	18.95

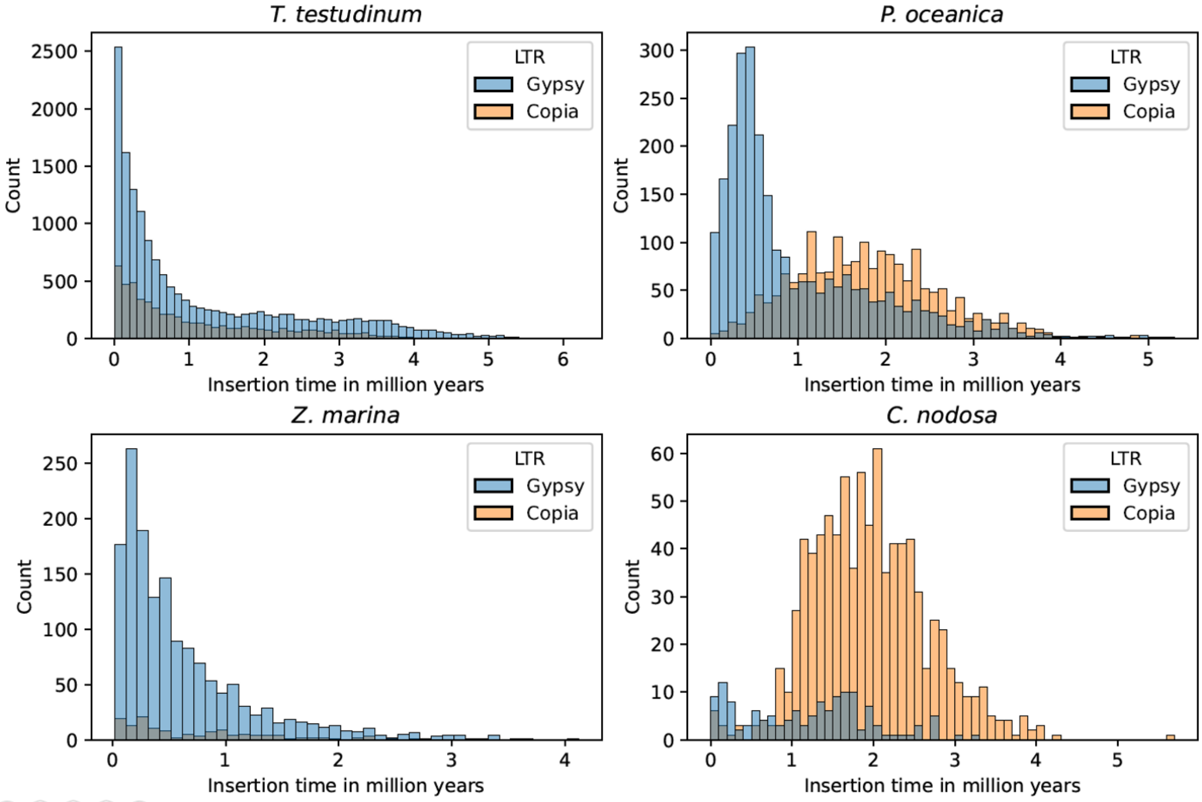

Supplementary Figure 4.1 Insertion time distributions of LTR/Gypsy and LTR/Copia in *T. testudinum*, *P. oceanica*, *C. nodosa* and *Z. marina*. Note differences in the scale of the y-axis. See main text for details.

4.2 Identifying Whole Genome Duplications (WGD)

Supplementary Note 4.2.1 K_S age distributions

K_S age distribution analysis was performed using the wgd package (Zwaenepoel and Van de Peer 2019b). The paranome (the entire collection of duplicated genes) was obtained with 'wgd mcl' using all-against-all BlastP and MCL clustering. Anchor pairs (i.e., paralogous genes lying in collinear or syntenic regions of the genome) were obtained using i-ADHoRe (Simillion et al. 2008), employing the default settings in 'wgd syn'. K_S distribution analysis was also performed using the KSRATES software (Sensalari et al. 2021), which locates ancient polyploidization events with respect to speciation events within a phylogeny. It compares paralog and ortholog K_S distributions, while correcting for substitution rate differences across the involved lineages. First, an all-versus-all amino acid level similarity search for the set of protein-coding sequences was conducted using BLAST (v2.6.0+) with an E-value cut-off of 1e−10. The resulting sequence similarity graph was clustered by the Markov clustering algorithm mcl (v10-201) (Dongen 2008) with default inflation factor to identify the paralogous gene families. Second, a codon-level, multiple sequence alignment (MSA) was obtained by inferring an amino acid MSA using the MUSCLE (v3.8.31) (Edgar 2004) under default parameters, which was then back-translated to a codon-level nucleotide MSA. The gene families which had >200 members or an MSA length less <100 amino acids were filtered out. A maximum likelihood estimate of each gene pair in the family (for the pairwise synonymous distance (K_S)) was obtained using the CODEML program or the PAML (Yang 2007b) package (v4.9j). Third, an estimate of the phylogenetic tree topology of each paralogous gene family was obtained using Fasttree (v2.1.7) (Price et al. 2010) and rooted using midpoint rooting.

The most recent common ancestor (MRCA) node depth, of each gene pair, was associated with the pairwise K_S estimate as a weight, in which for each duplication node in the phylogenetic tree, all n pairwise K_S estimates of the descendant clades were added to the K_S distribution with a weight of 1/n to reduce redundancy. The K_S ortholog age distributions were based on the one-to-one orthologs (reciprocal best hits) between two species using BLAST (v2.6.0+) with an E-value cut-off of 1e−10 following the same K_S estimation process as for the paranome age distributions. The substitution rate correction across different species was achieved as follows: 1) The original divergence times between the focal species and the other species (represented as K_S distance) were obtained by the mode of the ortholog K_S distributions using bootstrapped kernel density estimation; 2) A substitution-rate-adjusted K_S estimate was calculated by transforming the original K_S distance into branch-specific K_S distance using the reference of outgroup species and then rescaling the K_S distance with the diverged species into the K_S timescale of the focal species for each divergence event. The corrected divergence times in K_S timescale were then compared with the paranome K_S distribution of focal species after above processes. The maximum number of outgroup species/trios selected to correct each divergent species pair was set as 6 and the consensus peak for multiple outgroups was set as best outgroup in KSRATES. Other parameters were set as default for rate correction using KSRATES. The species tree adopted consists of *Thalassia testudinum*, *Cymodocea nodosa*, *Posidonia oceanica*, *Potamogeton acutifolius*, *Zostera marina*, *Spirodela polyrhiza*, *Wolffia Australiana*, with *Brachypodium distachyon* and *Oryza sativa* as outgroup species, covering all the four families of seagrasses (Posidoniaceae, Zosteraceae, Hydrocharitaceae and Cymodoceaceae).

The syntenic analysis was performed by MCscan (Python version) with default parameters (Tang et al. 2008). Collinear blocks containing fewer than five orthologous gene pairs were filtered out. The collinearity information of the gene set and other genomic features in the seagrass's genomes were visualized by Tbtools and Circos(Krzywinski et al. 2009; Chen et al. 2020).

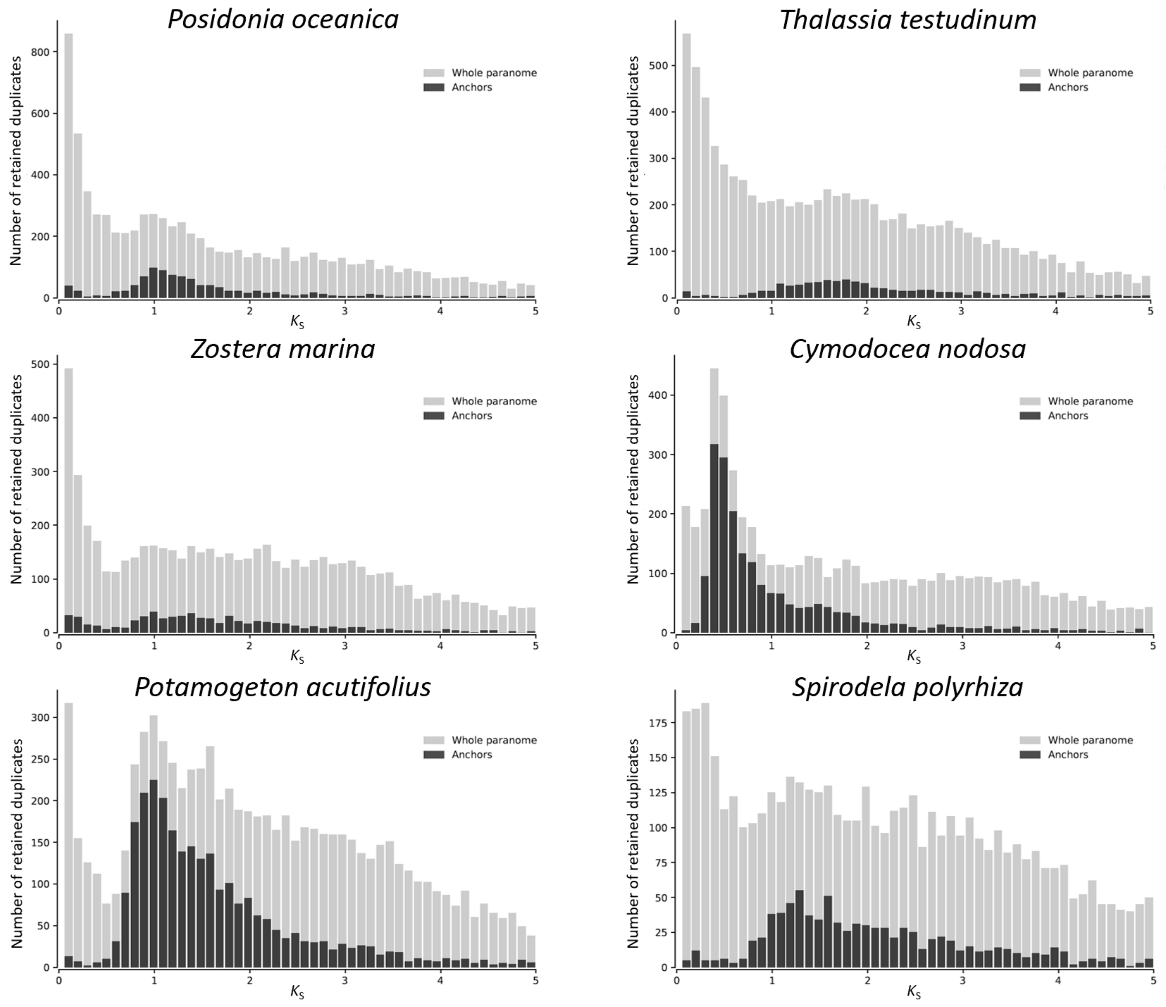

Supplementary Figure 4.2.1 K_S distributions for anchor pair duplicates (duplicates laying in duplicated, colinear blocks) and the whole paranome of four seagrasses, as well as for *P. acutifolius* and *S. polyrhiza*, generated by the wgd software (see Methods).

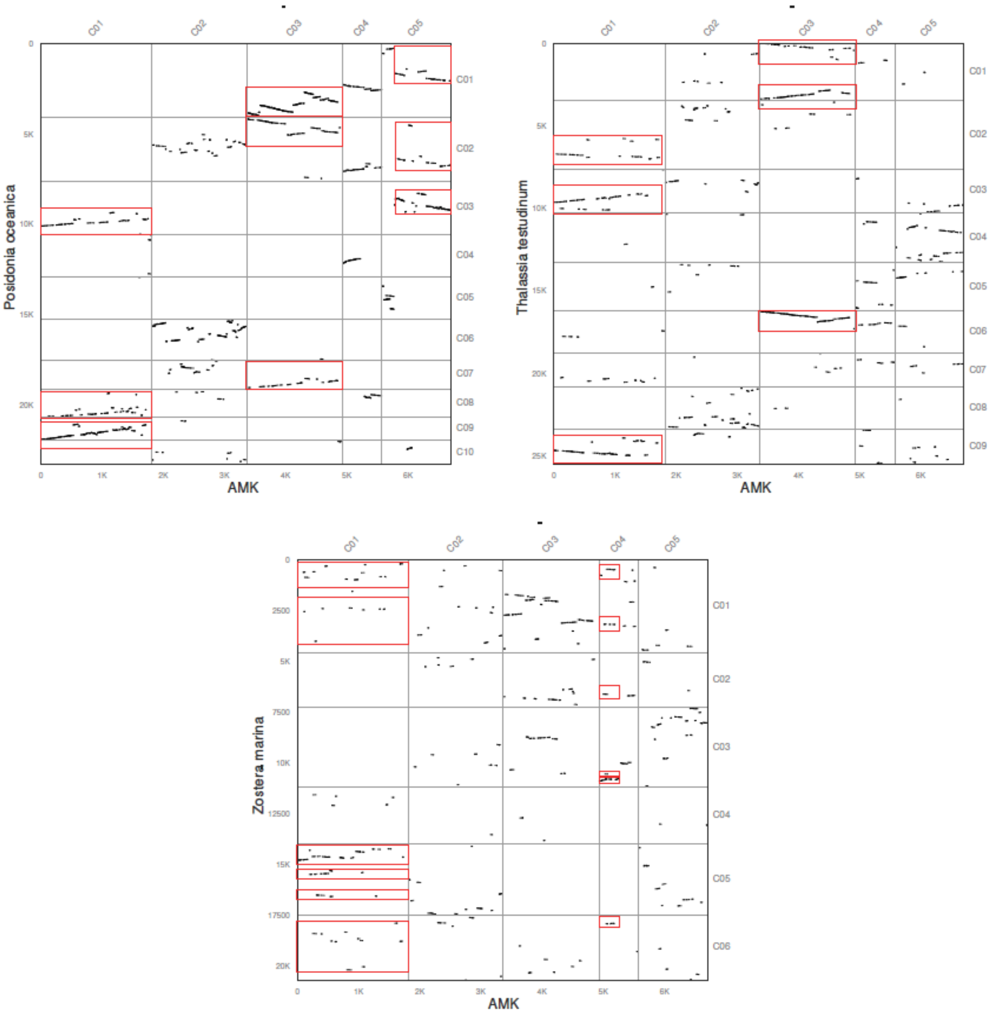

Supplementary Figure 4.2.2 Comparison of *Z. marina*, *P. oceanica*, and *T. testudinum* with a reconstructed ancestral monocot karyotype (AMK) (Murat et al. 2017).

Comparison between the AMK and *P. oceanica*, *T. testudinum* shows a clear 1:3 relationship, while the dot plot between the AMK and *Z. marina* shows a probable 1:6 relationship.

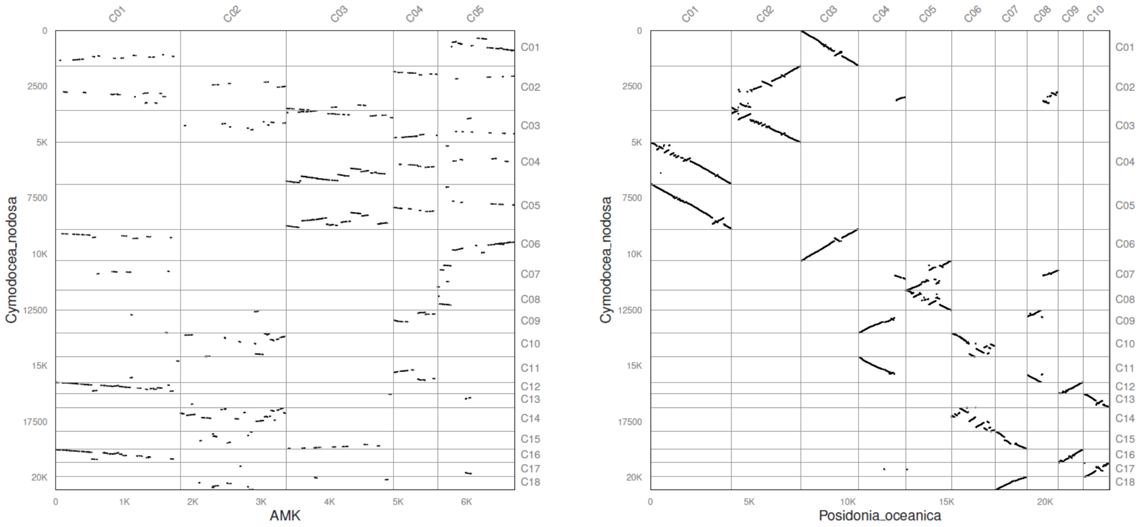

Supplementary Figure 4.2.3 Comparison of the ancestral monocot karyotype (AMK) (Murat et al. 2017) with *C. nodosa* and comparison of *P. oceanica* and *C. nodosa*.

Comparison between the AMK and *C. nodosa* shows a 1:6 synteny relationship. Comparison of *P. oceanica* and *C. nodosa* shows a 1:2 synteny relationship. This supports an extra WGD in *C. nodosa*, after its divergence with *P. oceanica*.

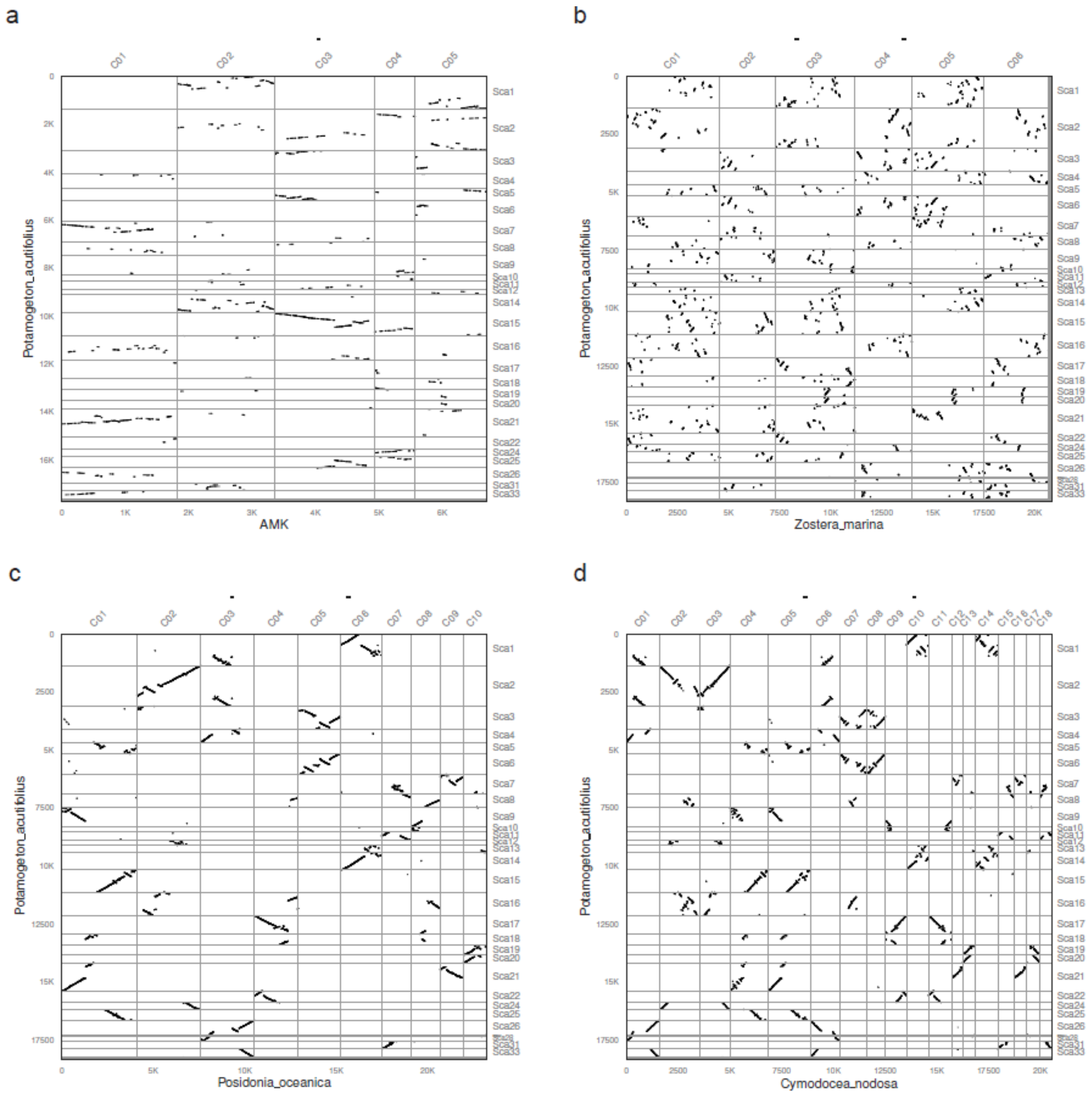

Supplementary Figure 4.2.4 Comparison of *P. acutifolius* with the ancestral monocot karyotype (AMK) (Murat et al. 2017), *Z. marina, P. oceanica,* and *C. nodosa*, respectively.

a) Comparison between *P. acutifolius* and the AMK shows a 1:6 synteny relationship. B) Comparison of *P. acutifolius* and *Z. marina* and shows a non-obvious synteny relationship c) *P. acutifolius* shows a 2:1 relationship with *P. oceanica*. D) *P. acutifolius* shows a 2:2 relationship with *C. nodosa*. See text for details.

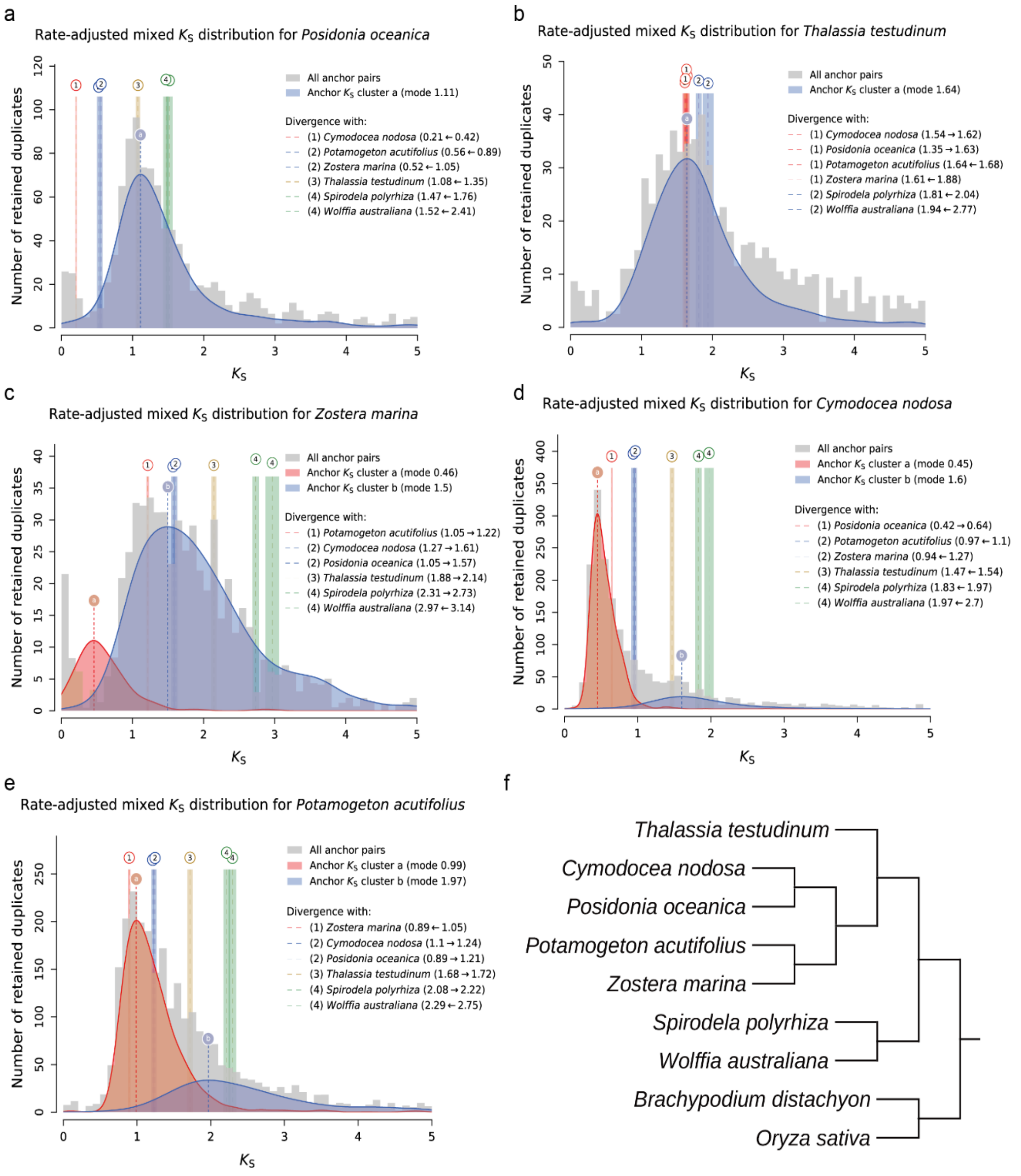

Supplementary Figure 4.2.5 K_S **Distributions for paralogs and the whole paranome of four seagrasses and *P. acutifolius* generated by KSRATES software.**

a – e, K_S distributions in *P. oceanica*, *T. testudinum*, *Z. marina*, *C. nodosa* and *P. acutifolius*. F, topology used in KSRATES analysis.

Supplementary Note 4.2.2 Gene tree-species tree reconciliation

OrthoFinder (Emms and Kelly 2019) (v2.3.3) was used to build orthologous gene families with the inflation factor set to 3.0 and the remaining parameters set as default. Gene families that did not have at least one gene from both clades at the root or have a family size exceeding 2 times the median of the square root of the family size, based on a Poisson outlier criterion, were filtered out (Zwaenepoel and Van de Peer 2019a). An amino acid, multiply sequence alignment (MSA) was obtained using PRANK (Loytynoja and Goldman 2005) for each gene family and the resulting MSA was then used as input for the Markov Chain Monte Carlo (MCMC) analysis in mrbayes (Huelsenbeck and Ronquist 2001) (v3.2.6) to sample from the posterior probability distribution. The rate matrix for amino acid data (Aamodelpr) was set to a fixed (LG) and the rate was set as gamma-distributed approximating four rate categories. The sampling frequency was set to 10 and the number of generations was set to 110,000 to reach a total of 11,000 posterior samples. The ALEobserve (Szöllõsi et al. 2013) was then used to construct the conditional clade distribution (CCD) containing marginal clade frequencies with a 'burn-in' of 1000, based on the 11,000 posterior samples for each gene family. The topology of the species tree was set as shown in Figure 3). The time-calibrated species tree was inferred by MCMCtree from the PAML package (Yang 2007a), using reference divergence times of 42-52 million years ago (MYA) for the most common ancestor of *Oryzae sativa* and *Brachypodium distachyon*, 118-129 MYA for that between *Spirodela polyrhiza* and *Zostera marina* and 130-140 for that between *Spirodela* and other terrestrial monocots (An et al. 2019).

The duplication-loss (DL)+WGD model, under critical and relaxed branch-specific rates, was implemented for the inference of the significance and corresponding retention rates of the assumed WGD events under Bayesian inference (BI) (Zwaenepoel and Van de Peer 2019a). In the critical-branch-specific DL+WGD model, the prior η, which denotes the parameter of the geometric prior distribution on the number of genes at the root, was set to follow a truncated-univariate-Beta distribution with shape parameters as (3,1) in the interval [0.01, 0.99]; the prior r, which denotes the mean of the branch rates distribution, was set to follow a flat distribution; the prior σ, which denotes the deviation of the branch rates distribution, was set to follow an exponential distribution with scale 0.1, λ (which denotes the duplication rate of each branch) was set to follow a multivariate normal distribution. For each branch, the loss rate μ was set to be equal to λ, while in the relaxed branch specific model, λ and μ were independent with the rates variation parameter τ set to follow an exponential distribution with scale 1 (Zwaenepoel and Van de Peer 2019a). To estimate duplication and loss rate λ and μ per branch incorporating both small-scale gene duplications and WGDs, another model without WGD nodes where all branch lengths were set as 1, the prior η was set to follow a Beta distribution with shape as (3,1) and the duplication and loss rate were set respectively to follow a normal distribution with mean as 0 and standard deviation as 5 were implemented. The Bayes Factor was calculated using the "bfact.jl" script within the public github repository of WHALE to measure the strength of evidence in favor of the assumed WGD models using the Savage-Dickey density ratio.

In total, 9 WGD(T) models were set on the branches leading to the MRCA of Potamogetonaceae, Zosteraceae, Posidoniaceae, Cymodoceaceae and Hydrocharitaceae (labelled as WGD1 or WGT1), the MRCA of Potamogetonaceae, Zosteraceae, Posidoniaceae and Cymodoceaceae (WGD2 or WGT2), the *C. nodosa* lineage (WGD3), the *P. acutifolius* lineage (WGD4), the *T. testudinum* lineage (WGD5 or WGT5), the MRCA of *S. polyrhiza* and *W. australiana* (WGD6), the *S. polyrhiza* lineage (WGD7), the *W. australiana* lineage (WGD8), the MRCA of *B. distachyon* and *O. sativa* (WGD9), respectively (Supplementary Figure 4.2.6). Posterior mean of duplication (left) and loss (right) rates estimated under DL+WGD modelling colored on the time-calibrated species tree. In the left panel, green squares indicate the significantly supported WGDs under the relaxed branch-specific model while empty squares indicate the WGDs that are not significantly supported under the relaxed branch-specific model. In the right panel, light green squares indicate the significantly supported WGDs under the critical branch-specific model while empty squares indicate the WGDs that are not significantly supported under the critical branch-specific model.

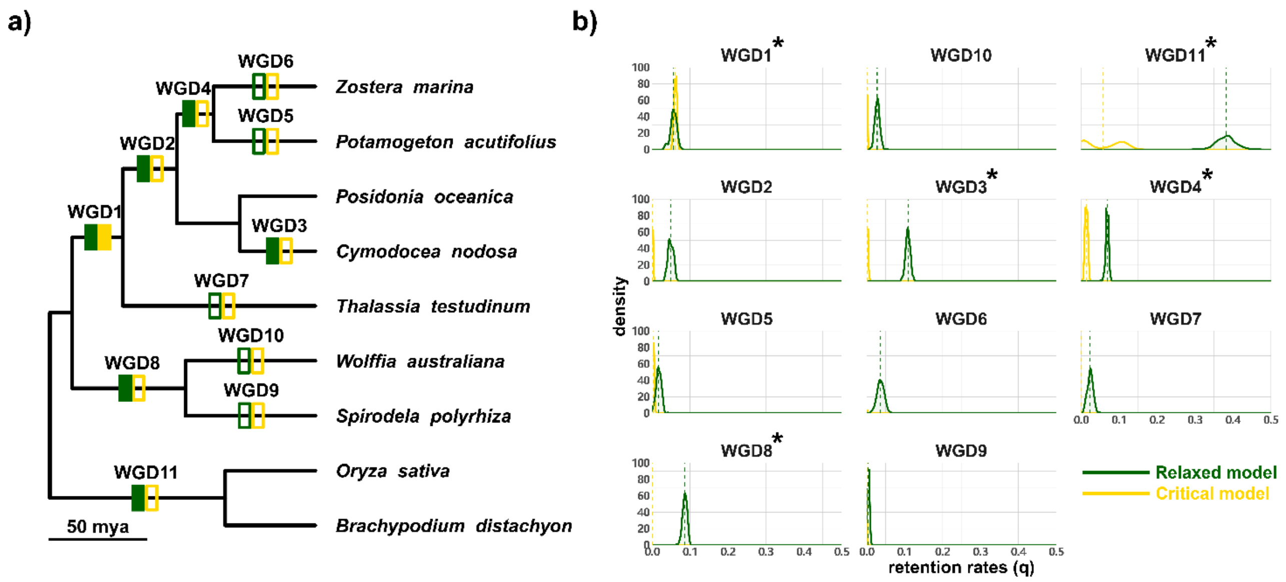

Supplementary Figure 4.2.6 Bayesian inference of retention rates (q) of 11 hypothetical WGD models in WHALE (Zwaenepoel and Van de Peer 2019a).

a) Summary representation of the support from 'relaxed' and 'critical' models on a 'divergence time tree', in which filled rectangles denote support in terms of Bayes Factor, while outlined rectangles denote lack of support. The green rectangles show the results from the relaxed model, while the yellow rectangles show the results using the critical model. B) Posterior distribution of retention rates of the 11 hypothetical WGD models. Model categories follow the same color code as in a). Hypothetical WGD models which gained significant support and 'accepted' in this study are marked with asterisks.

Supplementary Note 4.2.3 Absolute dating of WGDs

Absolute dating of WGD events was done as described previously described for *Zostera marina* (Olsen et al. 2016). Paralogous gene pairs located in duplicated segments (so called anchors) and duplicated pairs lying under the WGD peak (so-called peak-based duplicates) were collected for phylogenetic dating. Anchors, which are assumed to correspond to the most recent WGD, were detected using i-ADHoRe 3.0 (Simillion et al. 2008). For each WGD paralogous pair, an orthogroup was created that included the two paralogues plus several orthologues from other plant species, as identified by InParanoid (v4.1), using a broad taxonomic sampling, i.e., one representative from the order Cucurbitales, two from the Rosales, two from the Fabales, two from the Malpighiales, two from the Brassicales, one from the Malvales, one from the Solanales, two from the Poales, one orthologue from *Musa acuminata* (Zingiberales), and one orthologue from *Spirodela polyrhiza* (Alismatales). WGT/WGD paralogues were then dated using the BEAST v1.7 package under an uncorrelated relaxed clock model with the LG+G (four rate categories) evolutionary model. A starting tree with branch lengths satisfying all fossil-prior-constraints was created according to the consensus APGIII phylogeny. Fossil calibrations were implemented using log-normal calibration priors on the following nodes: the node uniting the Malvidae based on the fossil Dressiantha bicarpellate (Gandolfo et al. 1998) with prior offset = 82.8, mean = 3.8528, and s.d. = 0.5), the node uniting the Fabidae based on the fossil *Paleoclusia chevalieri* (Crepet and Nixon 1998) with prior offset = 82.8, mean = 3.9314, and s.d. = 0.5, the node uniting the Alismatales (including *Zostera marina* and *Spirodela polyrhiza*) with the other monocots based on the oldest fossil monocot pollen, Liliacidites (Doyle et al. 2008; Iles et al. 2015) from the Trent's Reach locality, with prior offset = 125, mean = 2.0418, and s.d. = 0.5 (Janssen and Bremer 2004; Nauheimer et al. 2012) and the root with prior offset = 124, mean = 4.0786, and s.d. = 0.5 (Smith et al. 2010). A run without data was performed to ensure proper placement of the marginal calibration prior distributions. The Markov chain Monte Carlo (MCMC) for each orthogroup was run for 10^7 generations, sampling every 1,000 generations, resulting in a sample size of 10^4. The resulting trace files of all orthogroups were evaluated manually using Tracer v1.570 with a burn-in of 1,000 samples to ensure proper convergence (minimum ESS for all statistics at least 200). To resolve the absolute dates of other involved WGDs in our analysis (Figure 2), we also redated the WGDs of *Elaeis guineensis*, *Asparagus officinalis*, *Rhizophora apiculata*, *Avicennia marina* and *Utricularia gibba* using the same pipeline as above. Moreover, the fossil of *Sabalites carolinensis* (Berry 1914) were also chosen as calibrations as previous study (Vanneste et al. 2014). The final WGD dates were shown in Supplementary Table 4.2 and Supplementary Figure 4.2.8.

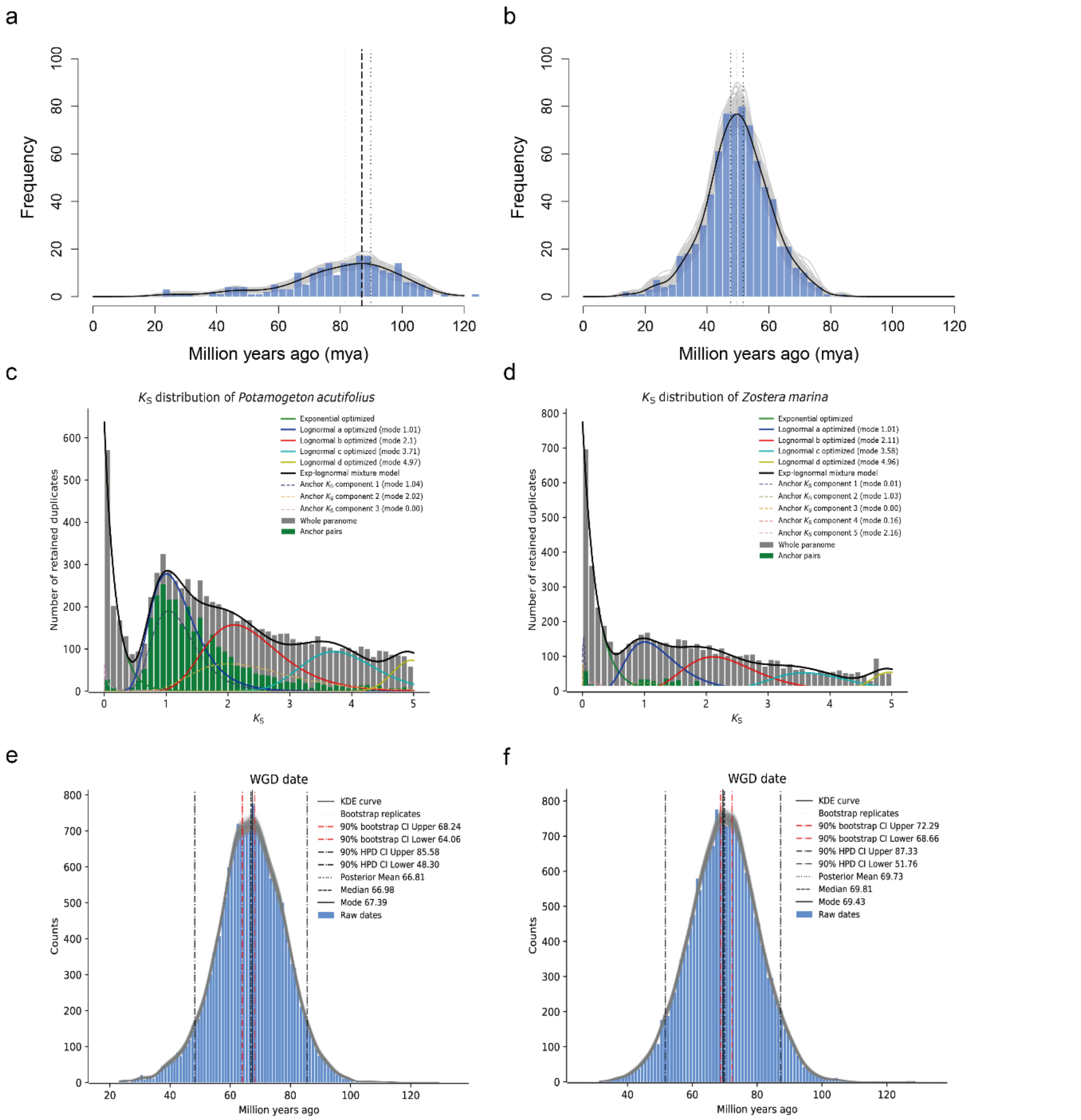

Supplementary Figure 4.2.7 Estimation of the 'absolute age' of the WGT/WGD events in seagrasses and *P. acutifolius* by phylogenomic dating.

a) Estimation of the 'absolute age' of the *P. oceanica* WGT event by phylogenomic dating. The solid black line represents the KDE (Kernel Density Estimation) of the dated paralogues, while the vertical dashed black line represents its peak at 86.96 Mya, which was used as the consensus WGT age estimate. The grey lines represent density estimates from 2,500 bootstrap replicates (after we obtained the age estimates for each accepted orthogroup that satisfied the condition that the minimum ESS for every parameter is larger than 200, we calculated the bootstrap 90% confidence interval for the mode of fitted kernel density estimation (KDE) using the "boot" library in R upon these age estimates), while the vertical black dotted lines represent the corresponding 90% confidence interval for the WGD age estimate, 89.89 - 79.81 Mya. The histogram shows the raw distribution of dated paralogues. b) Estimation of the 'absolute age' of the recent *C. nodosa* WGD event. Interpretation is as in a). c) The mixture modeling results of whole paranome and anchor K_S distribution of *P. acutifolius*. d) The mixture modeling results of whole paranome and anchor K_S distribution of *Z. marin.* e) Estimation of the 'absolute age' of the recent *P. acutifolius* WGD event. Interpretation is as in a). f) Estimation of the 'absolute age' of the recent *Z. marina* WGD event. Interpretation is as in a).

Supplementary Table 4.2 The absolute of WGD events taken from literature

Species name	Date	Family	Order	Clade	Phylogenetic position of WGD	Data Source
Spirodela polyrhiza	83-94, 95-100	Araceae	Alismatales	Monocots	Araceae	(Wang et al. 2014; An et al. 2019)
Oryza sativa	63.08-69.89,100-120,110-135	Poaceae	Poales	Commelinids	Poaceae, Poales, non-Alismatales monocots	(Tang et al. 2010; Vanneste et al. 2014; Ming et al. 2015)
Elaeis guineensis	48.25-55.97, 96.71-110.48	Arecaceae	Arecales	Commelinids	Partial Arecaceae, Arecales	**Our dating, see Supplementary Figure 4.2.8**
Asparagus officinalis	59.24-72.43	Asparagaceae	Asparagales	Monocots	Asparagus	**Our dating, see Supplementary Figure 4.2.8**
Arabidopsis thaliana	49.27-50.99	Brassicaceae	Brassicales	Rosids	Partial Brassicaceae	(Guo et al. 2018)
Populus trichocarpa	60-65, 125-140	Salicaceae	Malpighiales	Rosids	Specific to Populus and Salix, Core eudicots	(Tuskan et al. 2006)
Rhizophora apiculata	50.36-56.95	Rhizophoraceae	Malpighiales	Rosids	Rhizophoraceae	**Our dating, see Supplementary Figure 4.2.8**
Vitis vinifera	125-140	Vitaceae	Vitales	Rosids	Core eudicots	(International Peach Genome et al. 2013)
Avicennia marina	60.46-78.78	Acanthaceae	Lamiales	Asterids	*Avicennia marina*	**Our dating, see Supplementary Figure 4.2.8**
Utricularia gibba	43.37-53.99	Lentibulariacea e	Lamiales	Asterids	Utricularia	**Our dating, see Supplementary Figure 4.2.8**
Solanum lycopersicum	62.64-64.84	Solanaceae	Solanales	Asterids	Solanaceae	(Vanneste et al. 2014)

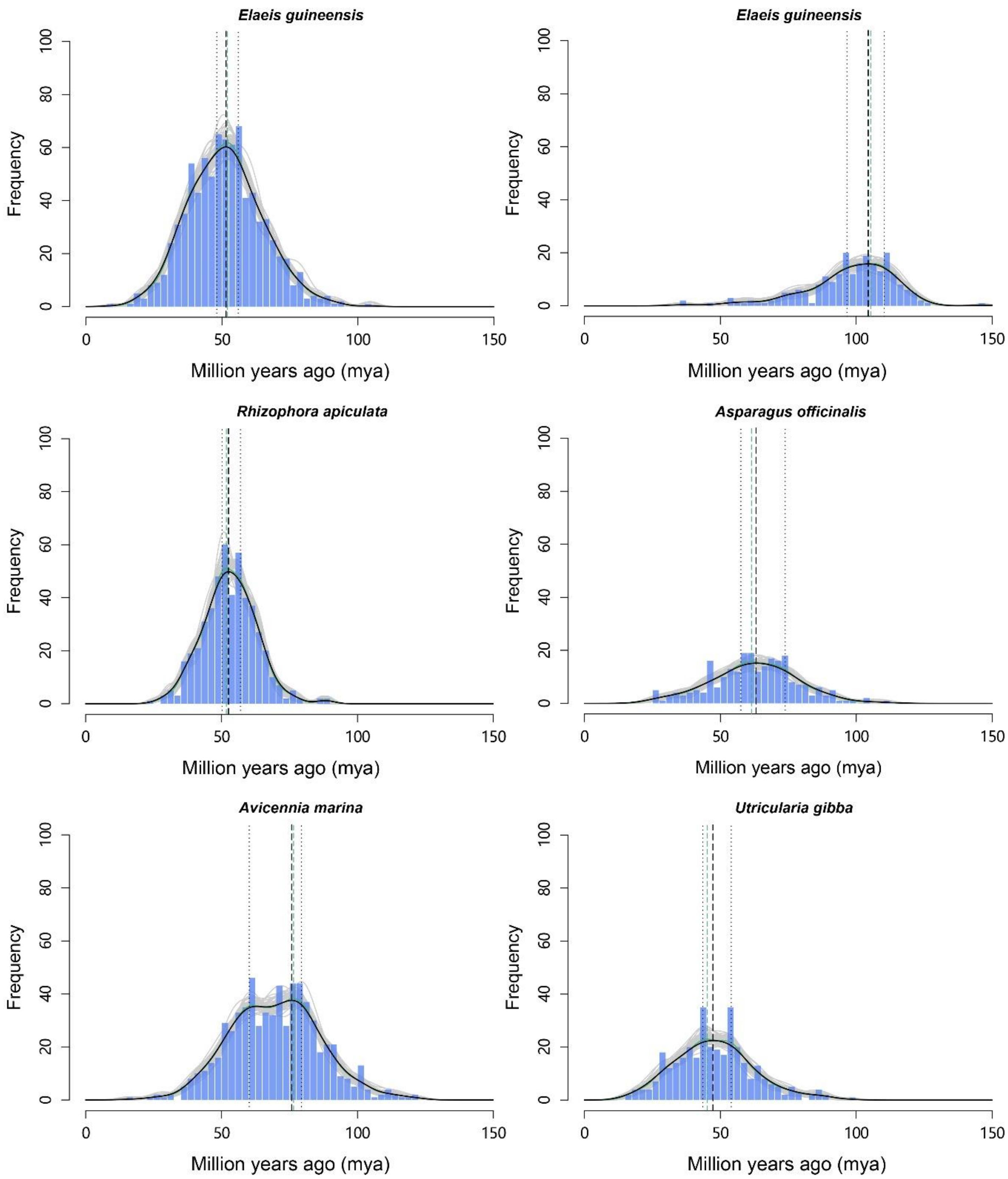

Supplementary Figure 4.2.8 Estimation of the 'absolute age' of seven independent WGD events experienced by *E. guineensis*, *A. officinalis*, *R. apiculata*, *A. marina* and *U. gibba* respectively by phylogenomic dating of corresponding paralogues.

The solid black line represents the KDE (Kernel Density Estimation) of the dated paralogues, and the vertical dashed black line represents the peak which was used as the consensus WGD age estimate. The grey lines represent density estimates from 2,500 bootstrap replicates and the vertical black dotted lines represent the corresponding 90% confidence interval for the WGD age estimate. The histogram shows the raw distribution of dated paralogues.

4.3 Phylogenetic tree construction and estimation of divergence time

Supplementary Note 4.3 Species selection and construction of time-calibrated phylogeny.

Protein sets were collected for 23 species, including *Oryza sativa* (PLAZA 5.0), *Brachypodium distachyon* (PLAZA 5.0), *Ananas comosus* (PLAZA 5.0), *Elaeis guineensis* (PLAZA 5.0), *Asparagus officinalis* (PLAZA 5.0), *Beta vulgaris* (PLAZA 5.0), *Utricularia gibba* (PLAZA 5.0), *Solanum lycopersicum* (PLAZA 5.0), *Coffea canephora* (PLAZA 5.0), *Vitis vinifera* (PLAZA 5.0), *Populus trichocarpa* (PLAZA 5.0), *Arabidopsis thaliana* (PLAZA 5.0), *Theobroma cacao* (PLAZA 5.0), *Avicennia marina* (PLAZA 5.0), *Spirodela polyrhiza* (PLAZA 5.0), *Amborella trichopoda* (PLAZA 5.0), *Wolffia australiana* (https://duckweeds.plantprofile.net/), *Rhizophora apiculata* (from the author), our four seagrasses and *Potamogeton acutifolius* (from ORCAE, https://bioinformatics.psb.ugent.be/orcae/). These species were selected as representatives for monocots and eudicots, and representing different habitats from terrestrial, freshwater-floating, freshwater-submerged, to marine-submerged. Orthofinder v2.3 (Emms and Kelly 2015) was used to delineate gene families with mcl inflation factor 3.0. All-versus-all Diamond blast with an E-value cutoff of 1e−05 was performed and orthologous genes were clustered using OrthoFinder. Single-copy orthologous genes were extracted from the clustering results. MAFFT (Rozewicki et al. 2019) with default parameters was used to perform multiple sequence alignment of protein sequences for each set of single-copy orthologous genes, and to transform the protein sequence alignments into codon alignments after removing the poorly aligned or divergent regions using trimAl (Capella-Gutiérrez et al. 2009). The resulting codon alignments from all single-copy orthologs were then concatenated into one supergene for species phylogenetic analysis. A maximum-likelihood phylogenetic tree of single-copy protein alignments and codon alignments was constructed using IQ-TREE (Minh et al. 2020) with the GTR+G model and 1,000 bootstrap replicates. Divergence times between the 23 plant species were estimated using MCMCtree from the PAML package under the GTR+G with reference divergence times of 124-170 MYA for the common ancestor of monocots and eudicots, 118-129 MYA for the divergence between *Spirodela* and *Zostera* and 130-140 MYA between *Spirodela* and other terrestrial monocots (An et al. 2019). We used MCMCTree to obtain 10,000 trees from the posterior sampling every 150 iterations after a burn-in of 500,000 iterations. We compared two independent runs with each other to verify convergence and with a run of the MCMC algorithm under the prior alone to compare the posterior distribution for the node ages to the effective prior implied by the fossil calibrations.

5. Adaptation to the marine environment

5.1. Use it or lose it

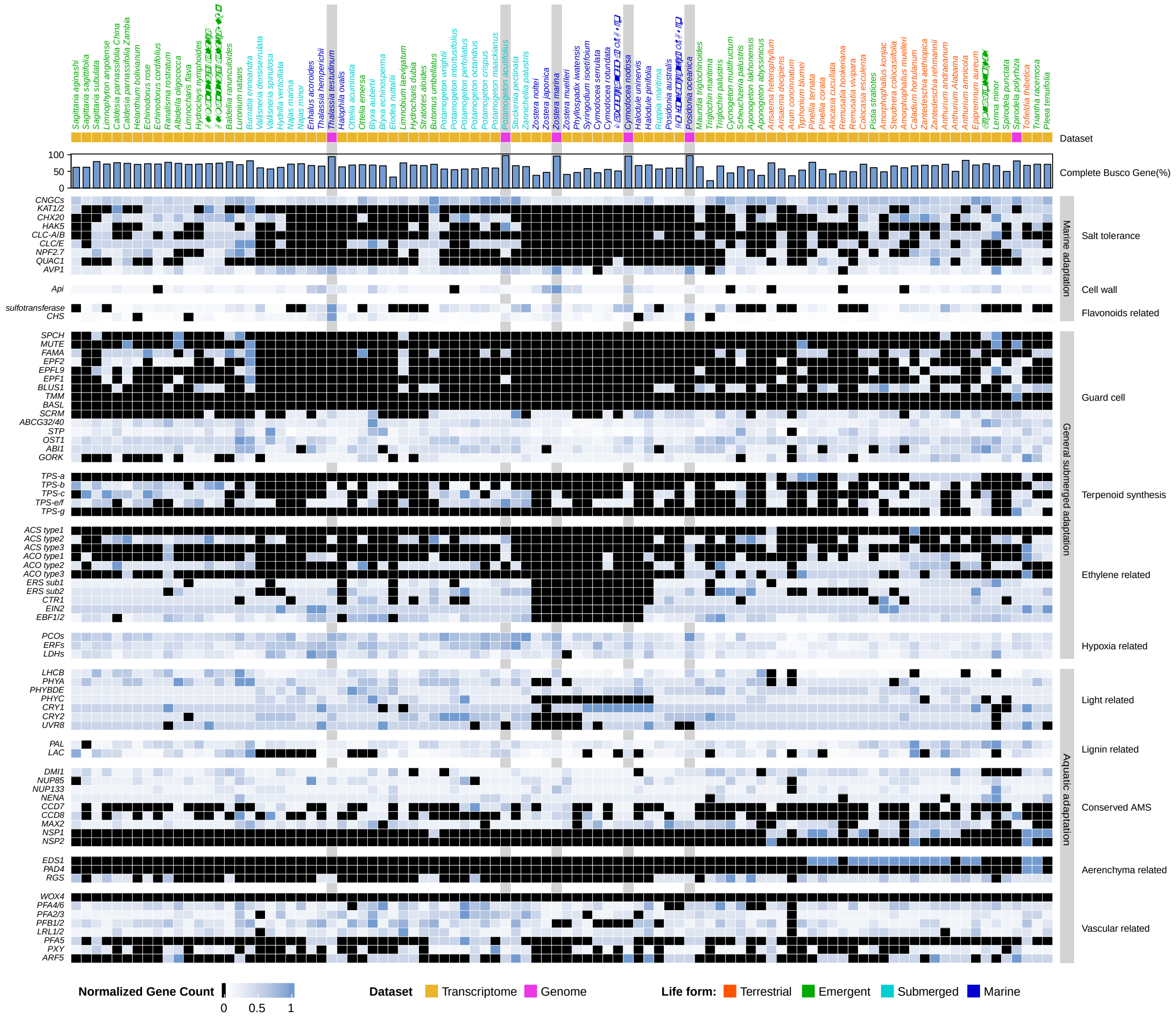

Supplementary Figure 5.1 Normalized gene counts for each species. Species with light grey backgrounds denote seagrass species and one freshwater relative, *Potamogeton acutifolius*, discussed in the present study. Other species are discussed in (Chen et al. 2022). Taxon order is phylogenetic. Normalization for each gene family was obtained by dividing the number of genes in that gene family for a particular species by the largest gene copy number within that family (considering all species). Genes in black are absent.

5.2 Pathogen resistance (R-) genes

Supplementary Note 5.2 Pathogen resistance gene

Effector-triggered immunity is one of the two main arms of the plant immune system and allows angiosperms to specifically detect pathogen effectors or their impact on host proteins. The detection is guided by nucleotide-binding leucine rich repeat receptors (*NLR*s), which is one of the largest gene families in plants, and under diversifying selection (Jacob et al. 2013). Of the two main domains of *NLR* resistance genes, the nucleotide binding site (NBS) domain is responsible for downstream signaling, and the leucine rich repeat (LRR) domain binds the target. *NLR* genes are often difficult to identify in genomes. Therefore, we used two software packages, NLR-Annotator (Steuernagel et al. 2020) and NLGenomeSweeper (Toda et al. 2020), followed by manual curation in a genome browser.

The number of *NLR* genes is strongly reduced (N=44) in *Z. marina* (Olsen et al. 2016) and similar reductions have since been found in many freshwater species (N= 100 range) (Liu et al. 2021), which is far less than in terrestrial species (N=100-300-500 [2300 in wheat]). Thus, we expected to see a similar extreme reduction in our new seagrass species, but this was not the case. While reduced in comparison to terrestrial species the number of NLR genes was markedly higher than in *Z. marina*; *C. nodosa* (N=87), *P. oceanica* (N=95) and *T. testudinum* (N=54).

Confirming our general hypothesis of convergent evolution at the genomic level, these seagrass species have low counts of *NLR* gene copies (Supplementary Table 5.2). Further, NLRs with a TIR domain are completely absent, which is typical of many monocots, and a few genes are missing the LRR domain. We also found that 30-40% of gene copies are non-functional in *C. nodosa*, *P. oceanica*, *T. testudinum* and *P. acutifolius*, either because of stop mutations or partial copies. By contrast, only 8% are non-functional in *Z. marina* (Supplementary Table 5.2).

The *NLR* gene copies occur in clusters towards the terminal ends of the chromosomes consistent with findings in other plants (Jacob et al. 2013). These clusters are made up of tandem copies as evidenced by their relationship shown in the NBS-domain-based phylogenetic tree and chromosomal location (Supplementary Figure 5.2.1 and Supplementary Figure 5.2.2). Here, they cluster into several clades, each including all of the species, thus indicating that the ancestor also contained these gene lineages. Similarly, when incorporating other more distantly related species in the NBS tree (not shown), the seagrass-genes-branches are distributed throughout, so old *NLR* gene lineages are still maintained, despite the reduction in total number compared to other plants. Single lineages are expanded into clusters of dozens of copies at the species level, especially in *C. nodosa* and *P. acutifolius* (Supplementary Figure 5.2.1). From an evolutionary perspective, clustering is considered as a reservoir of genetic variation (Jacob et al. 2013).

Supplementary Table 5.2 *NLR* gene counts by domain architecture and completeness in seagrasses and *P. acutifolius.*

Completeness was determined by the NLR-Annotator software. Complete/partial refers to the set of motifs needed for a functional gene; pseudogene refers to loci that do not have complete open reading frames. Gene class abbreviations in parentheses are a more general designation used in some papers.

Note: Due to their partly fragmented nature not all *NLR* genes are present in the genome annotation with gene IDs. The genomic coordinates of all identified NLR genes can however be found in Extended Data Table 5.

***NLR* gene class**	***Cymodocea nodosa***	***Posidonia oceanica***	***Thalassia testudinum***	***Zostera marina v3.1***	***Potamogeton acutifolius***
CC-NBS (CN)	2	0	0	0	2
CC-NBS-LRR (CNL)	59	58	27	21	71
NBS-LRR (NL)	26	37	27	23	40
NBS (N)	0	0	0	0	2
TIR-NBS (TN)	0	0	0	0	0
TIR-NBS-LRR (TNL)	0	0	0	0	0
**Total**	**87**	**95**	**54**	**44**	**115**
**NLR gene completeness**					
Complete	69	62	42	26	87
Complete pseudogene	20	28	15	18	27
Partial	13	14	12	0	25
Partial pseudogene	44	41	13	4	17
**% Complete**	**61**	**62**	**70**	**92**	**73**

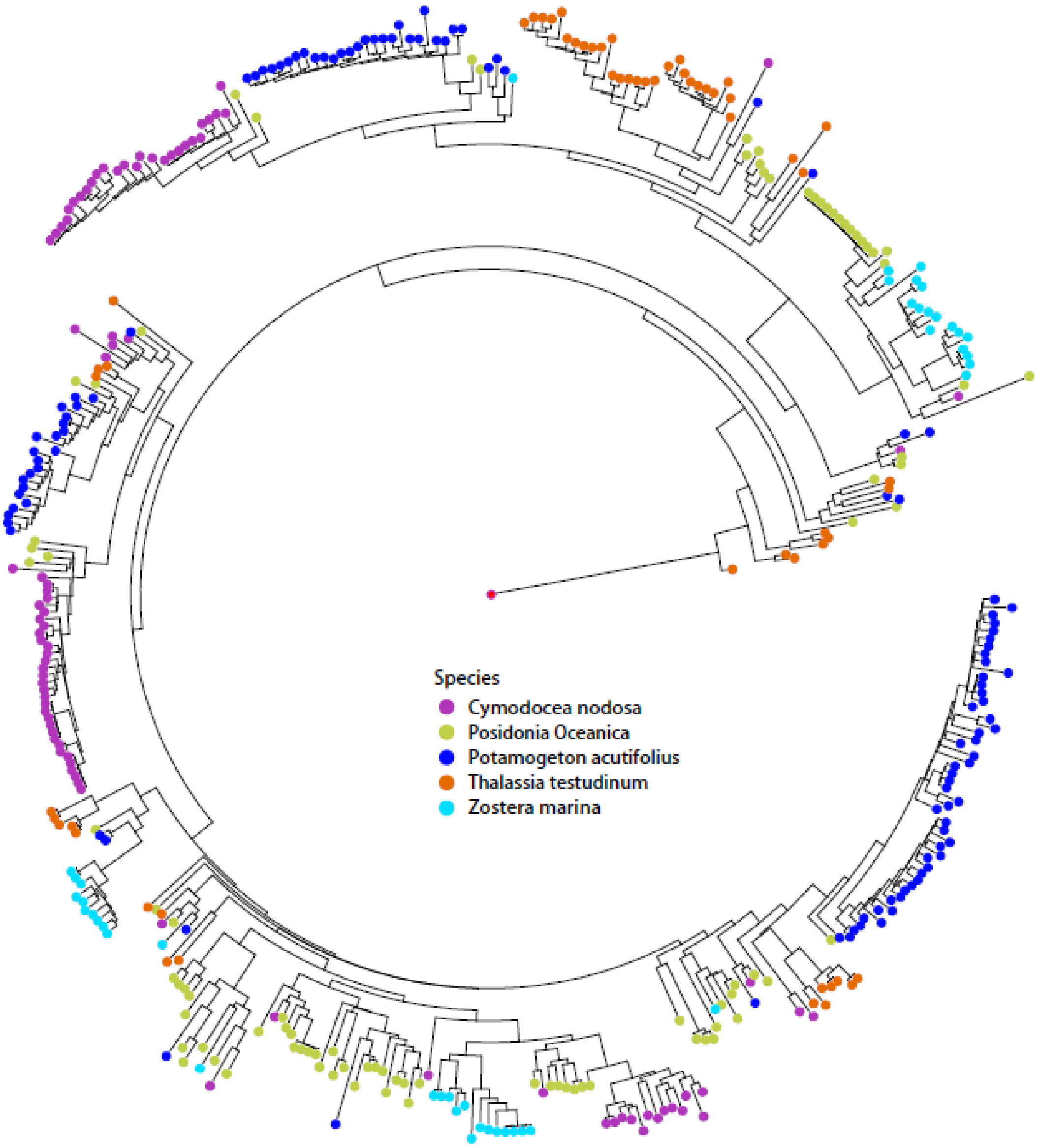

Supplementary Figure 5.2.1 Phylogenetic tree of seagrass *NLR* genes based on NBS domain.

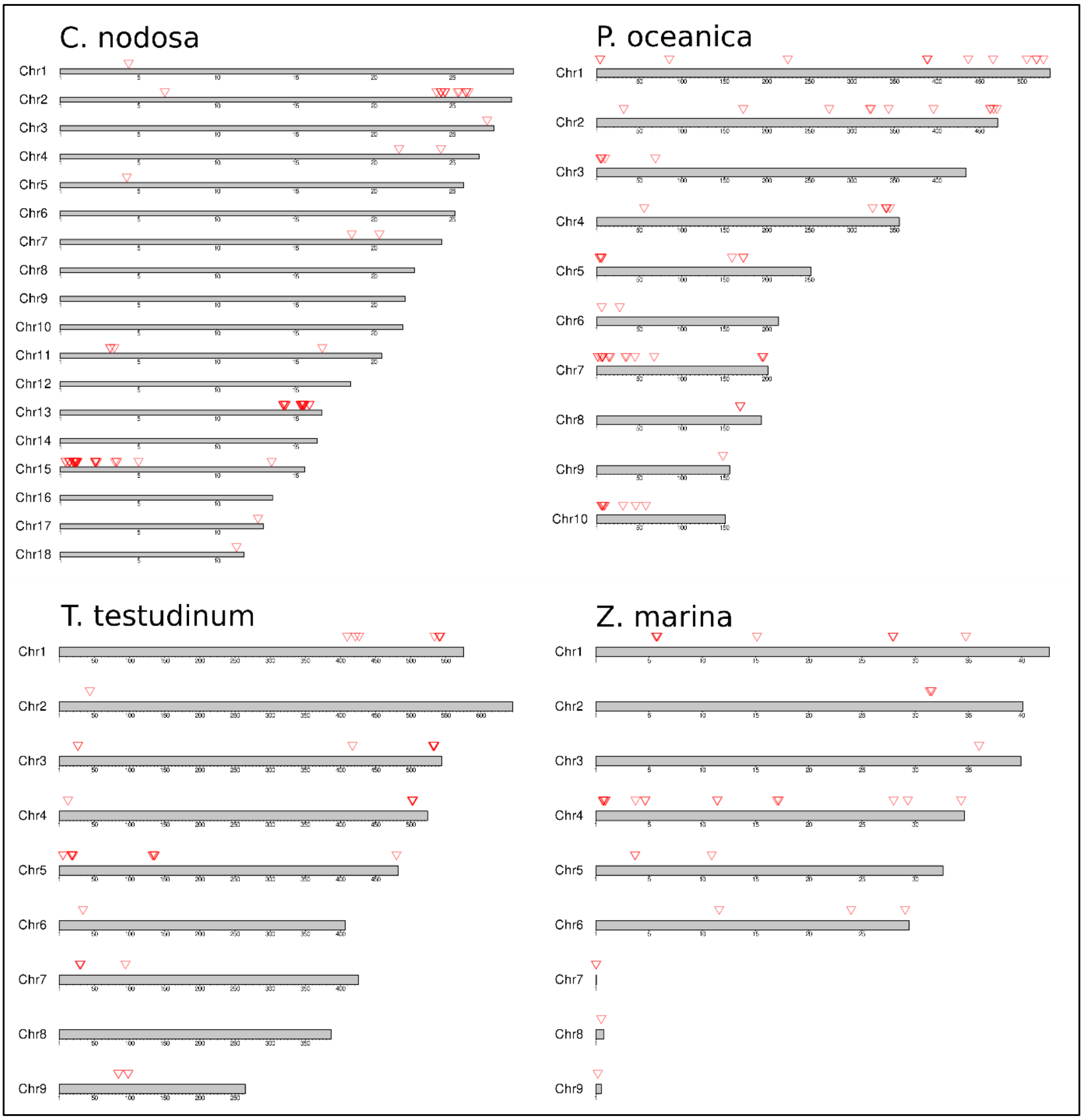

Supplementary Figure 5.2.2 Distribution of seagrass *NLR* genes across chromosomes.

NLR genes are indicated by red arrows and predominantly occur in the distal regions.

5.3 Heat Shock factor (*HSF*) gene family evolution

Supplementary Note 5.3 *HSF* gene family

Heat shock transcription factors (*HSFs*) are a family of DNA-binding proteins that activate a cascading network of genes that act together to enhance plant tolerance to abiotic stress conditions, including heat, cold, drought, hypoxia, salinity, toxicity and excessive irradiance (Scharf et al. 2012). Based on the topology of their domains, *HSFs* are classified into three major classes (*HSFA*, *HSFB* and *HSFC*), which are further subdivided into 16 subfamilies: *HSFA1-HSFA9*, *HSFB1-HSFB5* and *HSFC1-HSFC2*. Individual *HSFs* have unique functions as part of different signal transduction pathways operating in response to environmental stress and during plant development (von Koskull-Döring et al. 2007). To examine the different composition of *HSF* gene families in seagrasses, *HSF* families of *Cymodocea nodosa*, *Posidonia oceanica*, *Thalassia testudinum* and *Zostera marina*,

were compared with those of the freshwater plant *Pomatogeton acutifolius*, ten terrestrial eudicots (*Amborella trichopoda, Arabidopsis thaliana, Beta vulgaris, Solanum lycopersicum, Populus trichocarpa, Vitis vinifera, Coffea canephora, Theobroma cacao* and the mangroves *Avicennia marina* and *Rhizophora apiculata*), five terrestrial monocots (*Oryza sativa, Brachypodium distachyon, Elaeis guineensis, Ananas comosus* and *Asparagus officinalis*) and three other freshwater plant species (one eudicot: *Utricularia gibba*; two monocots: *Spirodela polyrhiza* and *Wolffia australiana*). *HSF* sequences were searched in the Plant Tanscriptional Factor Data Base (PlantTFDB, http://planttfdb.gao-lab.org/) and in Phytozome 13 (https://phytozome-next.jgi.doe.gov/). Protein sequences were subsequently downloaded from PLAZA (https://bioinformatics.psb.ugent.be/plaza/), the PlantTFDB or, when needed, from the species genome web page (*Wolffia australiana*: https://duckweeds.plantprofile.net/; *Utricularia gibba*: http://genomevolution.org/CoGe/). Sequences were then uploaded to the HEATSTER platform to check their identity as *HSF*s and to use a single criterion for their classification within the 16 *HSF* sub-classes. Classification and annotation were performed in HEATSTER via two successive steps of repeated searches in a motif database (motifs: DBD and OD with HR-A and HR-B region). In those cases where a specified sequence did not contain all *HSF*-associated motifs, classification was based on the recognition of the most conserved domain (DBD) with an E-value < 1e-20.

In our analysis, the average number of *HSF*s in land plants (Supplementary Table 5.3) was similar to the values recently reported in a comprehensive study involving 29 eudicots and 10 monocots (Wang et al. 2018). We found that aquatic plants have a lower number of *HSF*s than terrestrial plants (54% on average), with no clear differences between marine and freshwater species. The four studied seagrass species have on average 11.8 (± 1.0) sequences recognized as *HSF*s, with all three types of *HSF*s (A, B and C) showing a strong contraction. The average number of class A and B members is reduced by 67.4% and 43% respectively in seagrasses compared to terrestrial monocots (Supplementary Table 5.3). In addition, some subclasses are completely absent in seagrasses (Extended Data Table 5). In the *HSFA* class, subclass A3, A7, A8 and A9 are lacking in seagrasses but are common in terrestrial monocots (except A9 found only in eudicots). Along with their role in the response to heat stress, these subfamilies have specialized functions in the response of plants and seeds to different abiotic stresses, mainly dehydration, drought stress and oxidative stress (Sakuma et al. 2006; von Koskull-Döring et al. 2007; Scharf et al. 2012; Personat et al. 2014). The emergence of new functionalities has been associated with the weak purifying selection of these subfamilies in terrestrial plants (Wang et al. 2018). Contrarily, subfamilies A1, A2, A5 and A6, which are subjected to more severe selection pressure and directly involved in the heat stress response (Heerklotz et al. 2001; Mishra et al. 2002; Scharf et al. 2012; Xue et al. 2014), are represented in seagrasses. These results reveal that marine plants have lost several subclasses of *HSFA* previously acquired to cope with the stress conditions associated with a terrestrial lifestyle.

Regarding the *HSFB* class, subclasses B3 and B5 are not present in seagrasses, but neither are they present in terrestrial monocots as they presumably arose after the split of monocots and eudicots (Scharf et al. 2012; Guo et al. 2016). Seagrasses retained B1 and B2 subclasses (Extended Data Table 5), both of which are involved in promoting the activity of *HSFA1*, which is the master regulator of the heat stress response in *Arabidopsis* (Ikeda et al. 2011; Scharf et al. 2012). Moreover, *HSFB1* form a triad with *HSFA1* and *HSFA2* in tomato, acting as synergistic coactivator of heat stress responsive genes during the exposure and recovery to high temperatures (Mishra et al. 2002; Scharf et al. 2012). It is therefore evident that marine plants have conserved all major *HSF* subclasses that functionally cooperate in the heat stress response and thermotolerance of plants (A1, A2, A5, A6, B1 and B2).

HSFC family members (i.e., C1 and C2) are completely absent in seagrasses. Although the function of class C *HSF*s is the least known, they appear to be integrated into signaling pathways not directly related to the heat stress response (Scharf et al. 2012). *HSFC2* act as transcriptional activator of heat shock protein (*HSP*) genes in wheat during heat, drought and salt stress (Xue et al. 2014), and is up-regulated in rice by oxidative and heat stress (Mittal et al. 2012) . Similarly, *HSFC1* showed altered expression levels in Arabidopsis under several stress conditions, including cold stress, freeze stress and dehydration stress (Lee et al. 2005; Xin et al. 2007; Ding et al.

2013; Zhuang et al. 2018). These results indicate that the functionally specialized *HSFC* family, which emerged during the evolution of plants towards a terrestrial lifestyle, has been lost when plants returned to the sea.

In summary, plants with an aquatic lifestyle, including those adapted to the marine environment (seagrasses), have a reduced number of *HSFs* compared to terrestrial plants. Despite having a small number of members, seagrasses have retained those *HSF* subfamilies under strong purifying selection in land plants, probably to maintain important biological functions. Among them is the main group of *HSF*s directly related to heat stress response and thermotolerance in plants. In contrast, subfamilies of *HSF*s with specialized functionalities for plant adaptation to terrestrial habitats and not directly related to heat stress response but to other types of abiotic stresses (e.g., drought) have been lost in seagrasses. The greater homogeneity and stability of environmental conditions at sea relative to those on land is the most likely cause of these changes. Finally, only tropical seagrasses retained some of the key heat stress-related *HSF*s from WGD and WGT events (*C. nodosa*: *HSFA1* and *HSFB4*; *T. testudinum*: *HSFB2*), which could be related to their warmer native environment and higher heat stress tolerance compared to temperate seagrasses (*P. oceanica* and *Z. marina*).

Supplementary Table 5.3 Average (± SD) number of total *HSF*s and number of *HSF*s from the three main classes (*HSFA*, *HSFB* and *HSFC*) in the analyzed plant genomes.

The basal angiosperm *Amborella trichopoda* (11 sequences) and the freshwater eudicot plant *Utricularia gibba* (21 sequences) were not included in the table.

Plant group	No. species	Total *HSFs*	*HSFA*	*HSFB*	*HSFC*
TERRESTRIAL	14	28.0 ± 10.0	16.5 ± 6.6	8.3 ± 3.7	2.1 ± 1.8
Eudicots	9	26.1 ± 10.3	15.1 ± 6.5	8.3 ± 4.3	1.2 ± 0.9
Monocots	5	31.4 ± 9.7	19.2 ± 6.5	8.4 ± 2.5	3.8 ± 1.8
AQUATIC (monocots)	7	12.9 ± 3.3	7.4 ± 2.6	5.4 ± 1.3	0.0 ± 0.0
Freshwater	3	14.3 ± 5.1	9.0 ± 3.6	5.3 ± 1.5	0.0 ± 0.0
Seagrasses	4	11.8 ± 1.0	6.3 ± 0.5	5.5 ± 1.3	0.0 ± 0.0

5.4 Cellular salt tolerance

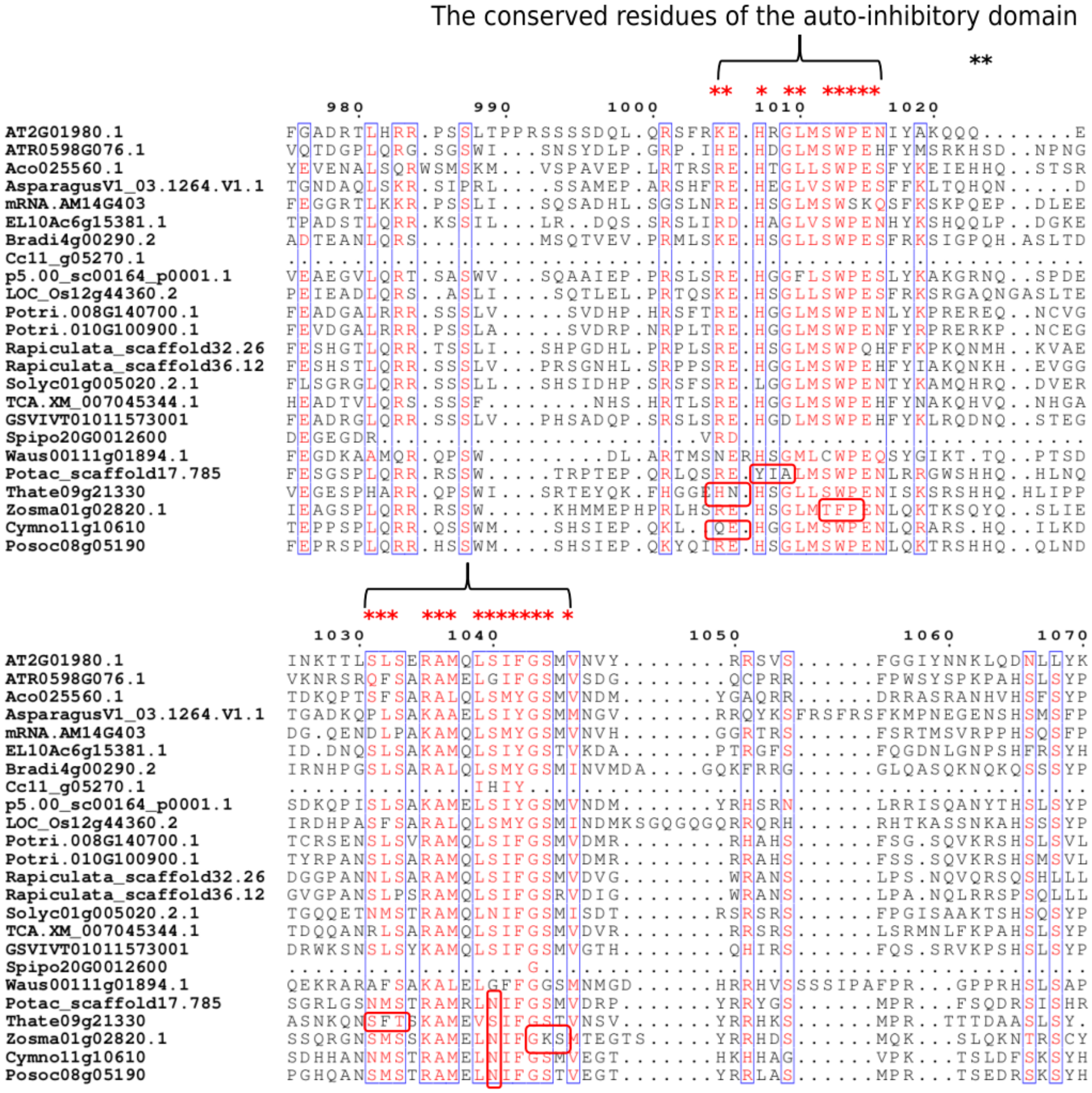

Supplementary Figure 5.4.1 Sequence alignment showing amino acid substitutions in regulatory domains of *SOS1* orthologs of seagrasses, indicating a diverged but convergent regulation of *SOS1*/*NHX7* in these species.

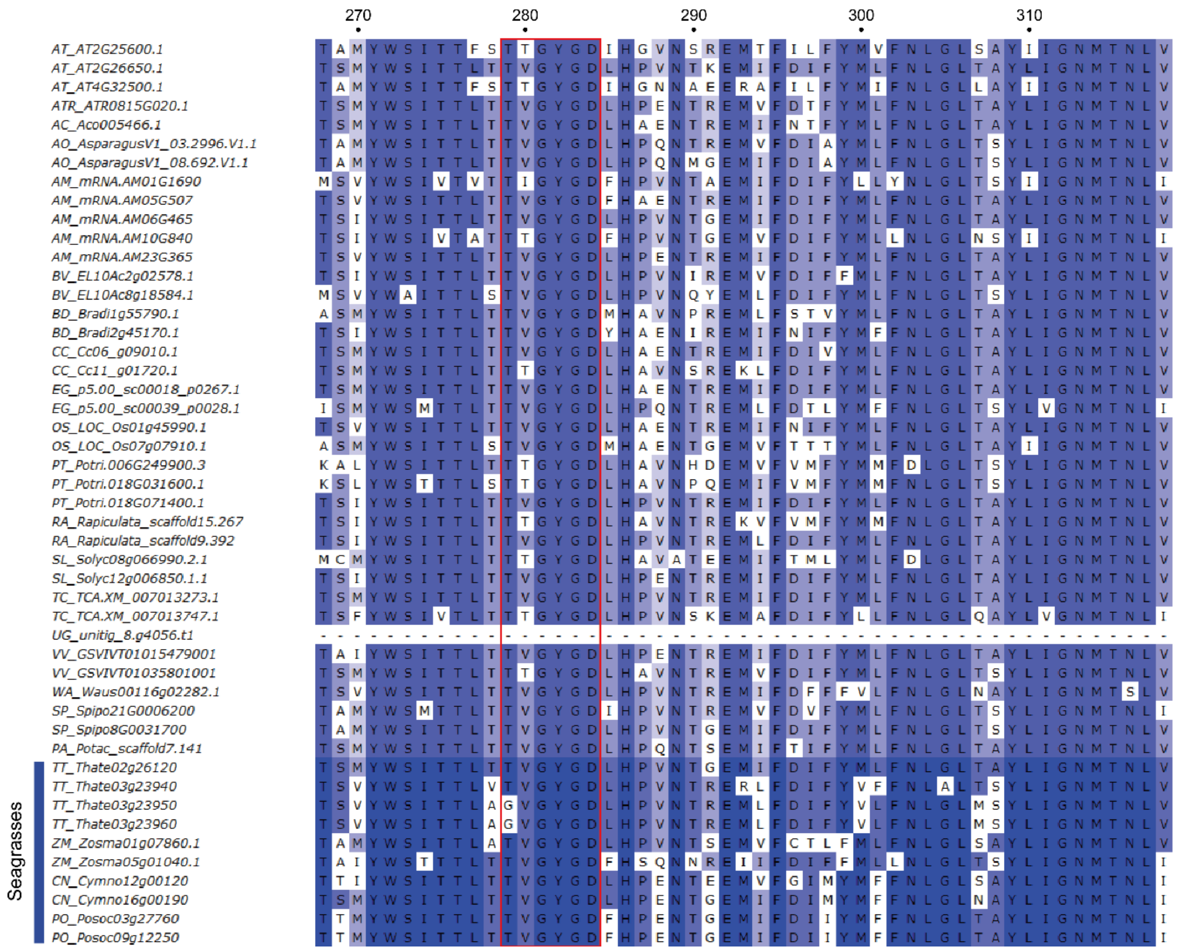

Supplementary Figure 5.4.2 Sequence alignment of *AKT5/6/1* showing the loss of Shaker-type K+ channels with a TTGYGD-selectivity filter in all seagrasses.

A TTGYGD-selectivity filter is indicated by the red box; Full list of abbreviation of the species names used in the figure: AT *Arabidopsis thaliana*; SL *Solanum lycopersicum*; ATR *Amborella trichopoda*; AC *Ananas comosus*; AO *Asparagus officinalis*; AM *Avicennia marina*; BV *Beta vulgaris*; BD *Brachypodium distachyon*; CC *Coffea canephora*; CN *Cymodocea nodosa*; EG *Elaeis guineensis*; OS *Oryza sativa*; PO *Posidonia oceanica*; PT *Populus trichocarpa*; PA *Potamogeton acutifolius*; RA *Rhizophora apiculate*; SP *Spirodela polyrhiza*; TT *Thalassia testudinum*; TC *Theobroma cacao*; VV *Vitis vinifera*; UG *Utricularia gibba*; WA *Wolffia australiana*; ZM *Zostera marina*.

5.5 Hypoxia

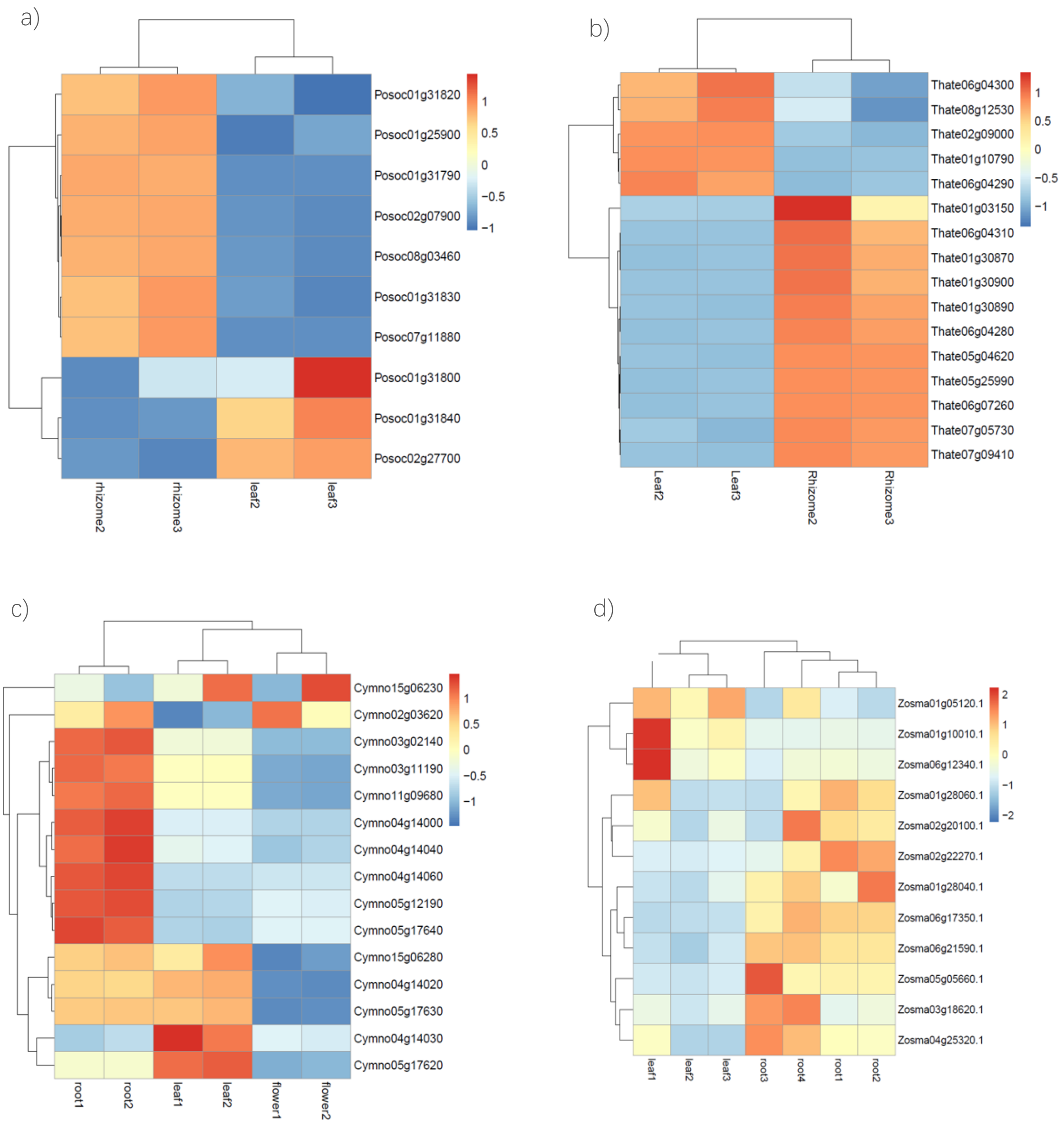

Supplementary Figure 5.5.1 Differential expression of *ERF-VII*s in the four seagrass species.

a. Leaf vs. rhizome in *P. oceanica*, b. Leaf vs. rhizome in *T. testudinum*, c. leaf vs. rhizome vs. flower in *C. nodosa*; d. leaf vs. root in *Z. marina*. Most *ERF-VII*s had higher expression in rhizomes and roots as compared to leaves in four seagrasses.

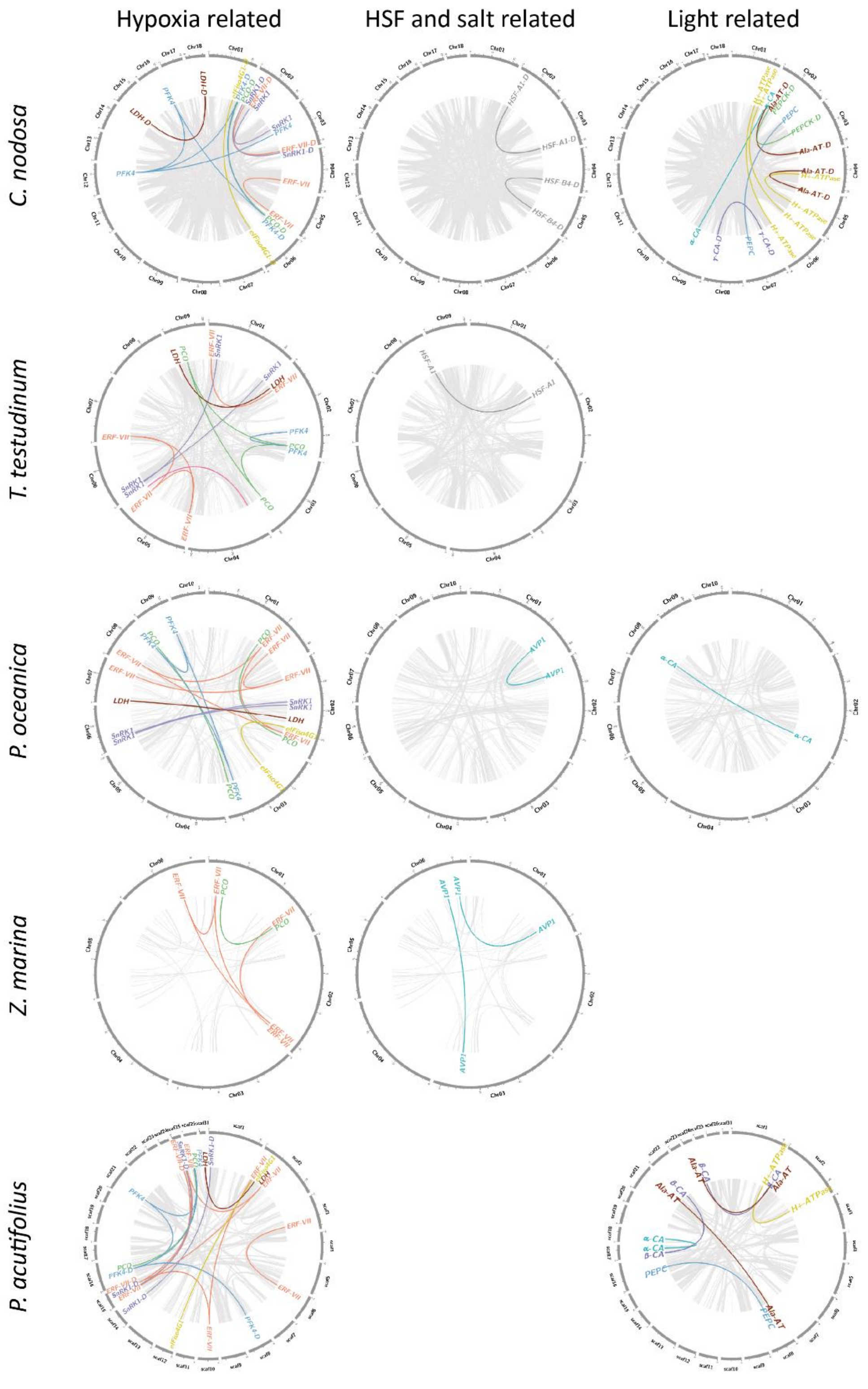

Supplementary Figure 5.5.2 Syntenic relationship of genes mentioned in the main text for *P. oceanica*, *T. testudinum*, *Z. marina*, *C. nodosa*, and *P. acutifolius*.

Different colors represent different families derived from the whole-genome triplication event, as well as from the whole-genome duplication events for *P. oceanica, T. testudinum, Z. marina, C. nodosa*, and *P. acutifolius*. Gene names without the -D suffix means they are derived from the WGT event; Gene names with -D suffix in *C. nodosa*, and *P. acutifolius* means they are derived from the WGD event.

5.6 Light perception, circadian clock, and photosynthetic carbon acquisition

Supplementary Note 5.6.1 CO_2-concentrating mechanisms (CCMs) and photosynthetic carbon acquisition

One of the major challenges that face seagrasses is the acquisition of inorganic carbon (Ci) for photosynthesis. Photosynthetic carbon limitation in the marine environment results from several physicochemical factors that restrict the supply of Ci to the leaf surface of seagrasses. Of the DIC (dissolved inorganic carbon) pool in seawater, the bicarbonate ion (HCO_3^-) accounts for nearly 90%, while the primary Ci source for RuBisCO ($CO_{2(aq)}$) is in limited supply (roughly 1% of the DIC pool) (Campbell and Fourqurean 2013). Many algae and terrestrial plants have thus evolved various carbon-concentrating mechanisms (CCMs) to enhance their photosynthetic capabilities under Ci limitation. The occurrence of true CCMs in seagrasses is currently still a matter of debate (Larkum et al. 2017; Larkum et al. 2018), although recent findings point to the existence of biophysical CCMs and demonstrate an evolutionary adaptation of RuBisCO kinetics across submerged angiosperms (Capó-Bauçà et al. 2022). This closely resembles what seen in eukaryotic algae rather than that of terrestrial C4 plants (biochemical CCMs). The acquisition of HCO_3^- for photosynthesis can occur via two (non-exclusive) basic models: (a) apoplastic conversion of HCO_3^- to CO_2 and OH^-, catalyzed by external carbonic anhydrases (*CA*); (b) direct uptake of HCO_3^- by anion transporters or an H^+-HCO_3^- symport based on H^+-ATPase pumps (Larkum et al. 2018). In *P. oceanica*, a direct HCO_3^- uptake via a fusicoccin-sensitive H^+-ATPase pump has recently been demonstrated (Rubio et al. 2017).

The four seagrass species studied, as well as *Potamogeton*, possess genes encoding for all three carbonic anhydrase gene families present in higher plants (i.e., α, β and λ) (DiMario et al. 2017). Six orthogroups (OGs) encode for *α-CA*, the most abundant family, two are associated to *β-CA* and two to *λ-CA*, respectively (Extended Data Table 9). Overall, 87% of seagrass *α-CA* are identified as extracellular/soluble proteins (Supplementary Table 5.6), which could be excreted from epidermal cells for catalyzing the conversion of HCO_3^- to CO_2 and OH^-, likely contributing to CCMs (Larkum et al. 2017). 64% of seagrass *β-CA* are targeted to the chloroplast, generally to the chloroplast stroma or thylakoid membranes, while 100% of *λ-CA* are targeted to the mitochondria (data not shown). This seems to confirm that seagrasses do have a requirement for an extracellular *CA* activity for adequate photosynthesis, as previously demonstrated by using chemical inhibitors (Larkum et al. 2017; Larkum et al. 2018; Capó-Bauçà et al. 2022). *α-CA* OG0013954 is exclusive of seagrasses (except for *T. testidinum*) and *P. acutifolius* (Extended Data Table 9). All sequences within this OG possess the typical *α-CA* domain, but most of them have variable mutations of the canonical His at the active site (data not shown), with unknown effects. Almost all OG0013954 members, as well as other *α-CA* transcripts, are highly expressed in leaf tissue (Supplementary Figure 5.6.1), which could support the hypothesis that they constantly increase the CO_2 concentration in the periplasmic space, thus enhancing its diffusive transfer to RuBisCO and the photosynthetic rate in seagrass leaves. *α-CA* OG0028785 appears unique to *Z. marina*. A slight expansion of *α-CA* genes is evident in seagrasses with respect to terrestrial and freshwater/brackish-water species, when considering the average number of genes per species (terrestrial: 7; freshwater/brackish: 6; marine: 8). This increase in copy number of *α-CA* in *P. oceanica* and *P. acutifolius* results from the WGT event as well as specific tandem duplications.

A C4 or C3-C4 intermediate photosynthetic metabolism could also contribute to CCMs in seagrasses. We screened the components of the C4 photosynthesis pathway as outlined in Rao et al. (2016). The analysis of C4-pathway related genes revealed that all genes typical of terrestrial plants are present in seagrasses (Extended Data Table 9). However, this is not diagnostic of a functional C4 biochemical pathway, as they have functions other than C4 photosynthesis. In addition, none of the studied species possesses the Ser residue characteristic of C4 Phosphoenolpyruvate carboxylase (PEPC) (data not shown). In *C. nodosa*, which has been previously hypothesized to be a C4 species (Koch et al. 2013), there were 15 C4-related genes retained specifically after WGT or WGD events, including two encoding for PEPC (retained after WGD). Similarly, in *P. acutifolius*, 17 C4-related genes were retained following WGT or WGD (Extended Data Table 9). We cannot exclude the presence

of some kind of C3-C4 intermediate metabolism at least in these species, similar to what observed in the freshwater hydrophyte *Hydrilla verticillata* (Hydrochariraceae), where facultative single cell C4 type photosynthesis is known to occur (Rao et al. 2002).

Four orthogroups are annotated as boron transporters (HCO_3^- transporter family) in the studied species (Extended Data Table 9). One orthogroup is completely absent in aquatic species (OG0012730). Regarding proton pumps (H^+-ATPases), only three OGs encoded for plasma membrane *H^+-ATPases* (Extended Data Table 9), whose role could be associated to bicarbonate symport for photosynthesis. Overall, seagrasses have less genes, on average per species, than terrestrial ones, but several of them were retained following WGT or WGD events (7 in *C. nodosa*, 6 in *P. oceanica* and 3 in *P. acutifolius*) (Extended Data Table 9). This confirms that *ATPase* pumps could still play a role in seagrasses for driving symport of different substances across the plasma membrane, as demonstrated by Rubio et al. (2017) in *P. oceanica* for bicarbonate uptake.

Supplementary Table 5.6 Prediction of sub-cellular localizations of α-Carbonic Anhydrases (*α-CA*) in the studied seagrass species and *P. acutifolius*.

The presence of transit and signal peptides for specific cellular compartments and the determination of the protein type (soluble vs. membrane) was inferred by using multiple bioinformatics approaches. Genes in red are retained after WGT or WGD (in red) events.

Orthogroup	*C. nodosa*		*P. oceanica*		*T. testudinum*		*Z. marina*		*P. acutifolius*	
OG0000299	Cymno02g02020	Endoplasmic reticulum, Membrane	Posoc02g30740	Extracellular, Soluble	Thate05g03940	Sec/SPI; Extracellular, Soluble	Zosma01g08990	Sec/SPI; Extracellular, Soluble	Potac_scaffold2.170	Sec/SPI; Extracellular, Soluble
	Cymno02g02030	Sec/SPI; Extracellular, Soluble	Posoc02g30790	Sec/SPI; Extracellular, Soluble	Thate05g03970	Sec/SPI; Extracellular, Soluble	Zosma01g09000	Sec/SPI; Extracellular, Soluble	Potac_scaffold2.171	Sec/SPI; Extracellular, Soluble
	Cymno02g02040	Sec/SPI; Extracellular, Soluble	Posoc02g35020	Sec/SPI; Extracellular, Soluble	Thate08g22080	Sec/SPI; Extracellular, Soluble	Zosma06g13140	Sec/SPI; Extracellular, Soluble	Potac_scaffold2.173	Sec/SPI; Extracellular, Soluble
	Cymno02g02050	Extracellular, Soluble	Posoc06g06700	Endoplasmic reticulum, Membrane	Thate08g22090	Sec/SPI; Extracellular, Soluble	Zosma06g16770	Sec/SPI; Extracellular, Soluble		
	Cymno02g02060	Lysosome/ Vacuole, Soluble			Thate08g22100	Sec/SPI; Extracellular, Soluble				
	Cymno09g06350	Sec/SPI; Extracellular, Soluble			Thate08g22270	Sec/SPI; Extracellular, Soluble				
OG0002316	Cymno04g00210	Extracellular, Soluble	Posoc01g00410	Sec/SPI; Extracellular, Soluble	Thate01g08320	Cytoplasm, Soluble			Potac_scaffold2.352	Cytoplasm, Soluble
					Thate01g08350	Sec/SPI; Extracellular, Soluble			Potac_scaffold9.158	Sec/SPI; Extracellular, Soluble
					Thate06g11790	Sec/SPI; Extracellular, Soluble				
OG0013954	Cymno09g03270	Sec/SPI; Extracellular, Soluble	Posoc08g07760	Sec/SPI; Extracellular, Soluble			Zosma06g19600	Sec/SPI; Extracellular, Soluble	Potac_scaffold17.661	Sec/SPI; Extracellular, Soluble
			Posoc08g07770	Sec/SPI; Extracellular, Soluble			Zosma06g20930	Sec/SPI; Extracellular, Soluble	Potac_scaffold17.663	Sec/SPI; Extracellular, Soluble
									Potac_scaffold18.302	Sec/SPI; Extracellular, Soluble
OG0028785							Zosma01g03190	Sec/SPI; Extracellular, Soluble		

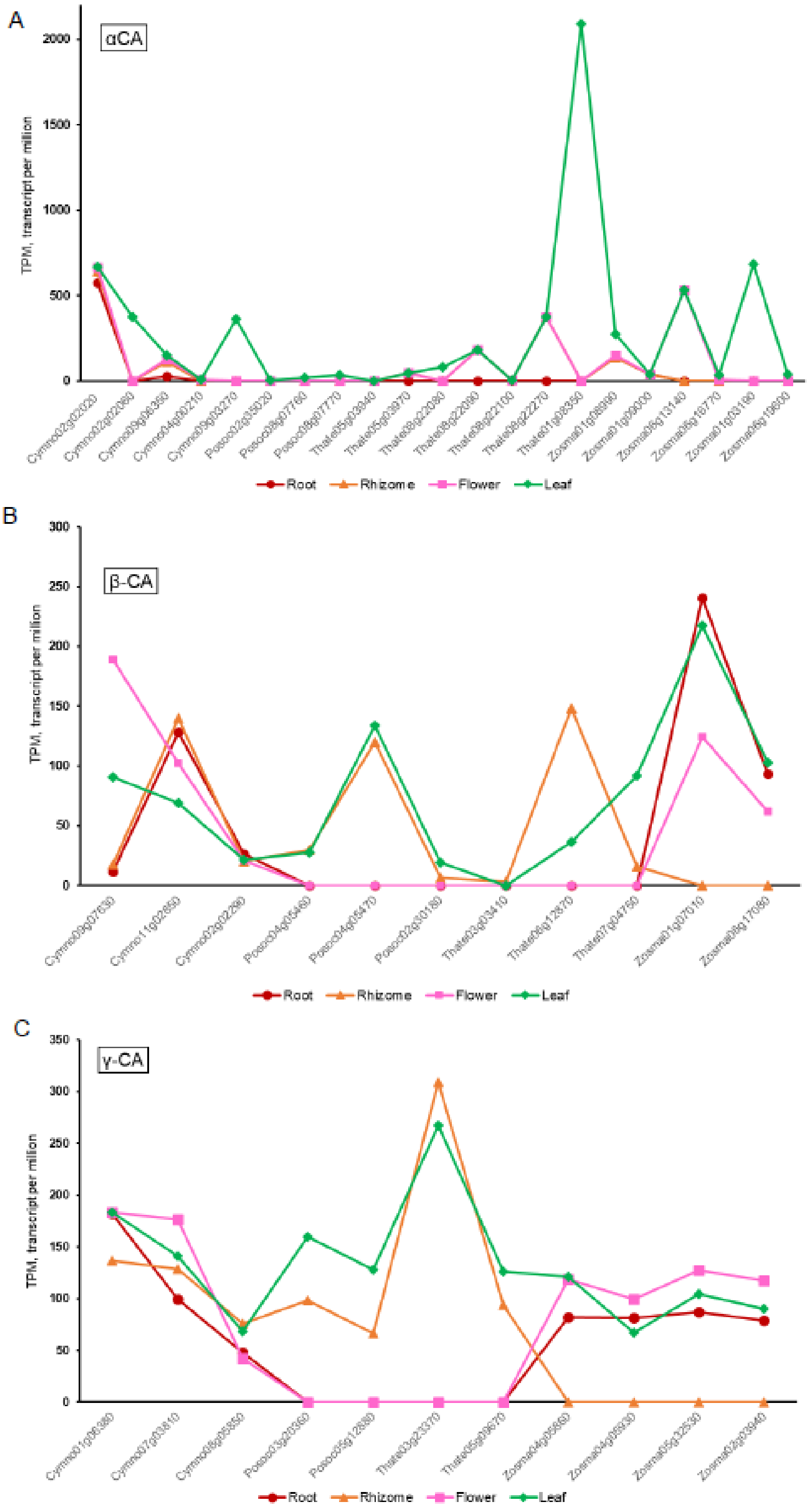

Supplementary Figure 5.6.1 Differential expression of *α-CA*, *β-CA* and *λ-CA* in root, rhizome, flower, and leaf tissues of the studied seagrass species.

Supplementary Note 5.6.2 Photosynthesis

Seagrasses face different light environments according to the depth and latitude where they live. Irradiance decreases with depth, and light quality is also altered along the water column. In order to investigate if the adaptation to the light environment experienced by seagrasses in their submerged marine life imposed evolutionary adaptations resulting in expansion/reduction of gene families, the following categories of genes have been analyzed (from KEGG Photosynthesis Proteins - *Arabidopsis thaliana*): Photosystem and electron transport system: Photosystem II (P680 chlorophyll a); Photosystem I (P700 chlorophyll a); Cytochrome b6/f complex; Photosynthetic electron transport; F-type ATPase; Antenna proteins: Light-harvesting chlorophyll-protein complex A and B (*LHCA*, *LHCB*). The total number of genes present in the orthology groups for the investigated gene families are presented in the Supplementary Figure 5.6.2. In comparison with the other Alismatales, seagrasses have a noteworthy expansion in genes of the *psA*/*psB* complexes and of the *LHCB*. Looking at the last group (*LHCB*) in particular, *Posidonia*, *Thalassia* and *Zostera* have a clear expansion in comparison to *Cymodocea*, which could be related to the larger depth gradient experienced by the formers. Genes for the *psA*/*psB* complexes are coded by the plastid genome, as well as for the Cytochrome b6/f complex and the F-type ATPase. Nevertheless, in most of case for seagrass species and for some of the other species included in the analysis there are also extra copies in the nuclear genome (numbers in red in the Supplementary Figure 5.6.2). *T. testudinum*, for example, has 6 nuclear copies of *psbA*. Nevertheless, nuclear copies of chloroplast genes are not expressed. See for example genes for the Cyt b6/f complex in *C. nodosa* and *Z. marina* leaf and flower tissues (Supplementary Figure 5.6.3 A and B).

Seagrass species analyzed feature different floral morphology. For two species (*Z. marina* and *C. nodosa*) gene expression data have been obtained also from floral tissue. Results indicate that *Zostera* flowers are photosynthetically active, while *Cymodocea* flowers are not. *LHC*s seem to be active at the same levels of leaf tissue while electron transport genes express at a lower level (Supplementary Figure 5.6.3 C and D).

In *Z. marina*, photosynthesis occurs mainly in pistillate (“female”) flowers. This could be due to the maturation timing of staminate (“male”) and pistillate flowers, as suggested for *P. oceanica*, where it was also found that only pistillate flowers expressed photosynthetic genes (Entrambasaguas et al. 2017).

			Seagrasses				Freshwater species			Mangrove		Moconots					Eudicots								
	Orthogroup	Gene name	CN	PO	TT	ZM	PA	SP	WA	AM	RA	OS	BD	AC	EG	AO	BV	UG	SL	CC	VV	PT	AT	TC	ATR
PsA	OG0001413	PsaA	2	4	2	2	0	0	0	0	1	5	3	1	0	0	5	4	16	1	2	6	1	0	0
	OG0004325	PsaB	1	1	3	1	0	1	0	0	3	3	2	2	0	0	7	2	4	0	2	1	1	0	0
	OG0012100	PsaC	1	1	1	1	0	0	0	0	0	3	2	1	0	0	1	0	1	0	1	1	1	0	0
	OG0004391	PsaD2	1	1	1	1	4	1	1	2	2	1	1	1	0	1	1	2	1	1	2	2	2	2	1
	OG0004272	PsaE	2	1	2	1	4	2	0	1	2	1	1	1	1	0	1	3	2	1	1	1	2	2	1
	OG0006616	PasF	1	1	1	2	2	2	2	2	1	1	1	1	0	0	1	1	2	1	0	1	1	1	1
	OG0012149	PsaI	1	1	1	1	0	2	0	0	0	0	4	0	0	0	0	0	2	0	1	1	1	0	0
	OG0012852	PsaJ	1	1	1	1	0	1	0	0	0	0	3	0	0	0	0	0	2	0	0	1	1	0	0
	OG0006947	PsaO	1	1	1	1	1	1	1	1	1	1	1	1	1	1	2	1	1	1	1	2	1	1	2
	OG0008605	PsaK	1	1	1	1	1	1	1	1	1	1	1	2	1	0	1	1	1	1	1	2	1	1	1
	OG0006761	PsaL	1	1	1	1	1	1	1	1	1	1	1	1	1	1	1	2	2	1	1	2	1	2	1
	OG0007957	PsaG	1	1	1	1	2	1	1	1	1	1	1	0	2	0	3	2	1	0	1	2	1	0	1
	OG0005369	PsaH	1	1	1	1	1	1	1	2	2	1	1	1	2	1	1	1	3	1	1	1	2	1	1
	OG0010572	PsaN*	1	1	1	1	1	1	1	1	0	1	1	1	0	0	1	1	1	1	1	2	1	1	1
	OG0010507	PsaN*	0	1	1	1	1	1	1	1	0	1	2	1	1	1	1	0	0	1	1	2	1	1	1
	OG0003908	PsaP	2	2	1	1	2	2	1	1	2	2	2	2	2	2	1	1	1	1	1	2	1	1	1
PsB	OG0001201	psbA	2	1	7	2	0	2	3	0	3	5	3	4	1	0	6	3	11	1	1	2	1	0	0
	OG0001495	psbB	1	3	1	2	0	1	1	0	4	5	7	6	1	1	9	1	3	0	2	3	1	0	1
	OG0003337	psbC	1	1	2	2	0	1	2	0	0	3	2	1	0	0	6	2	7	0	2	2	1	1	1
	OG0008341	psbD*	1	1	2	2	0	1	0	0	0	3	2	0	0	0	3	2	2	0	2	2	1	0	1
	OG0022254	psbD*	0	0	0	2	0	0	0	0	0	0	0	0	0	0	0	0	1	0	0	0	0	0	0
	OG0008317	psbE	1	1	1	2	0	1	0	0	0	3	5	1	0	0	5	0	1	0	1	1	1	0	1
	OG0013128	psbF	1	1	1	1	0	1	0	0	0	0	3	0	0	0	0	0	1	0	0	1	1	0	0
	OG0013809	psbL	1	1	1	1	0	0	0	0	0	0	3	0	0	0	0	0	0	0	0	1	1	0	0
	OG0013127	psbJ	1	1	1	1	0	0	0	0	0	0	4	0	0	1	0	0	1	0	0	0	1	0	0
	OG0010941	psbH	1	1	1	3	0	1	0	0	0	4	4	1	0	0	0	0	1	0	0	3	1	0	0
	OG0013442	psbI	1	1	1	1	0	0	0	0	0	0	1	0	0	0	0	0	1	1	0	2	1	0	0
	OG0011920	psbK	1	1	1	2	0	0	0	0	0	4	0	4	0	0	0	0	0	0	1	1	1	0	0
	OG0013443	psbM	1	1	1	1	0	0	0	0	0	0	1	0	0	0	0	0	1	1	0	2	1	0	0
	OG0005164	psbO	2	1	1	1	1	1	1	2	1	1	1	1	2	1	1	2	2	1	1	2	2	1	1
	OG0009830	psbP*	1	1	1	0	1	1	1	1	0	1	1	1	2	1	1	1	1	1	1	1	1	1	1
	OG0007493	psbP*	1	1	1	1	1	1	1	1	1	2	1	1	1	1	1	1	1	1	1	2	1	1	1
	OG0003480	psbP*	2	1	1	2	3	1	1	3	0	1	1	3	3	2	1	2	1	1	1	2	1	1	1
	OG0011018	pbsQ*	1	0	0	0	1	1	1	1	1	1	1	1	1	1	1	0	1	1	1	1	1	1	1
	OG0010574	pbsQ*	1	0	0	1	1	0	1	1	1	1	1	2	1	1	1	1	1	1	1	1	1	1	1
	OG0005668	pbsQ*	1	1	1	1	2	3	1	1	1	1	1	1	2	0	1	2	1	1	1	2	2	1	1
	OG0003547	psbR	1	2	2	1	2	1	1	2	1	3	3	1	2	1	1	1	2	1	1	4	1	1	1
	OG0005051	psbS	1	2	1	1	2	2	1	1	2	3	1	1	1	1	2	1	1	1	1	1	1	1	1
	OG0012853	psbT	1	1	1	1	0	1	0	0	0	0	3	0	0	0	0	0	0	0	0	3	1	0	0
	OG0003259	psbW	1	1	1	1	2	2	2	2	1	2	2	1	2	2	1	1	2	1	2	2	1	3	1
	OG0007350	psbY	1	1	1	1	1	2	1	0	2	1	1	0	2	1	1	3	1	1	0	2	1	1	1
	OG0011749	psbZ	1	1	1	1	0	1	0	0	0	4	1	2	0	0	0	0	3	0	0	1	1	0	0
	OG0007852	psb27	1	1	1	1	1	1	0	2	1	1	1	2	1	1	0	2	1	1	1	2	1	1	1
	OG0008772	psb28	1	1	1	1	3	1	0	1	1	1	1	1	1	1	1	1	1	1	1	1	1	1	1
Cytochrome b6/f complex	OG0002741	petA	2	2	2	4	0	1	0	0	0	5	6	1	0	0	6	1	3	1	2	2	1	0	1
	OG0006791	petB	1	1	1	2	0	0	0	0	0	3	5	4	0	0	2	0	3	0	0	4	1	0	0
	OG0004917	petC	1	1	2	1	4	1	2	1	1	1	1	1	1	1	1	1	1	1	3	2	1	1	1
	OG0008192	PetD	1	1	1	1	0	2	0	0	0	4	3	2	0	0	4	0	4	0	0	2	1	0	0
	OG0012851	PetG	1	1	1	1	0	1	0	0	0	0	4	0	0	0	0	0	1	0	0	1	1	0	0
	OG0013129	PetL	1	1	1	1	0	1	0	0	0	0	2	0	0	0	0	0	2	0	1	0	1	0	0
	OG0005169	petM	1	1	1	1	0	2	1	1	2	1	1	1	2	1	1	3	1	1	1	4	1	1	1
	OG0013068	petN	1	1	1	1	0	1	0	0	0	0	3	1	0	0	1	0	0	0	0	0	1	0	0
Photosynthetic e-transport	OG0003691	pet E	0	3	1	2	2	2	1	2	1	1	1	1	2	3	1	1	1	2	1	2	2	1	1
	OG0000289	petF*	2	3	2	1	12	2	12	5	2	5	6	5	5	3	3	3	7	4	1	7	4	2	3
	OG0008036	petF*	1	1	1	1	1	1	2	1	1	1	1	1	2	1	1	1	1	1	1	1	1	1	1
	OG0009442	petF*	1	1	1	1	1	0	2	1	1	1	1	1	1	0	1	1	1	1	1	2	1	1	1
	OG0004729	petH	1	1	1	1	2	1	1	1	1	2	2	1	1	2	1	1	2	1	2	2	2	1	1
	OG0006612	petJ	1	1	1	1	1	1	2	2	1	1	1	4	1	1	0	0	2	1	1	1	1	1	1
F-type ATPase	OG0000400	atpA	1	6	9	1	1	1	3	0	7	7	2	9	1	1	13	3	11	2	1	4	3	1	1
	OG0001351	atpB	2	2	1	7	0	0	1	0	2	6	5	3	0	0	7	2	3	1	0	2	1	0	9
	OG0005914	atpD	2	1	1	1	2	1	1	2	1	1	1	1	2	1	1	1	1	1	1	2	1	1	1
	OG0006895	atpE	1	2	1	3	0	0	0	0	0	5	5	1	0	1	1	0	1	0	0	1	1	0	2
	OG0007524	atpF	1	1	1	1	1	1	1	1	1	1	1	1	1	0	1	1	2	1	0	2	1	0	4
	OG0002439	atpG	2	1	1	1	5	1	1	4	2	1	1	1	3	3	2	2	2	2	0	3	2	2	1
	OG0008194	atpH	1	1	2	3	0	1	0	0	0	2	1	2	0	2	1	1	5	0	1	2	1	0	0
	OG0010619	atpI	1	1	2	2	0	1	0	0	0	2	4	1	0	0	1	1	4	0	0	1	1	0	0
	OG0007006	ATP synthase protein I	1	1	1	1	2	1	1	1	1	1	1	1	1	1	1	1	2	2	1	1	1	1	1
LHCA	OG0008288	LHCA1	1	1	1	1	2	1	1	1	1	1	1	1	0	1	0	1	2	1	1	2	1	1	1
	OG0001812	LHCA2	2	3	1	2	2	2	2	0	2	2	2	2	1	2	2	4	2	2	2	3	3	2	2
	OG0009534	LHCA3	1	1	1	1	1	1	1	1	1	1	1	1	0	1	1	1	2	1	1	1	1	1	1
	OG0005405	LHCA4	1	2	1	1	2	2	1	1	2	1	2	2	0	1	1	1	2	1	1	1	1	1	1
	OG0010862	LHCA5	1	1	0	1	1	1	1	0	1	1	1	1	0	1	1	1	1	1	1	1	1	1	1
LHCB Expansion	OG0007555	LHCB3	1	1	1	1	0	0	1	1	2	1	1	1	0	1	1	1	2	1	3	2	1	1	1
	OG0005086	LHCB4	1	1	1	1	1	1	1	1	3	1	1	2	0	1	2	1	1	1	0	3	3	2	1
	OG0000076	LHCB5+LHCB1/2	3	11	8	13	1	2	6	6	6	5	9	7	1	9	5	15	15	9	3	5	5	5	6
	OG0006482	LHCB6	1	2	1	1	1	1	1	2	1	1	1	1	0	1	1	2	2	1	1	2	1	1	1
	OG0009291	LHCB7	1	1	1	1	1	1	1	1	1	1	1	1	0	1	1	1	2	1	1	1	1	1	1

Supplementary Figure 5.6.2 Gene families containing photosystems I and II components and Light-harvesting chlorophyll protein complex. In red choloroplastic genes. Numbers exceeding 1 indicate the presence of nuclear copies. *different copies of the same gene belonging to different orthogroups. Full list of abbreviation of the species names used in the figure: CN *Cymodocea nodosa*; PO *Posidonia oceanica*; TT *Thalassia testudinum*; ZM *Zostera marina*; PA *Potamogeton acutifolius*; SP *Spirodela polyrhiza*; WA *Wolffia australiana*; AM *Avicennia marina*; RA *Rhizophora apiculate*; OS *Oryza sativa*; BD *Brachypodium distachyon*; AC *Ananas comosus*; EG *Elaeis guineensis*; AO *Asparagus officinalis*; BV *Beta vulgaris*; UG *Utricularia gibba*; SL *Solanum lycopersicum*; CC *Coffea canephora*; VV *Vitis vinifera*; PT *Populus trichocarpa*; AT Arabidopsis thaliana; TC *Theobroma cacao*; ATR *Amborella trichopoda*.

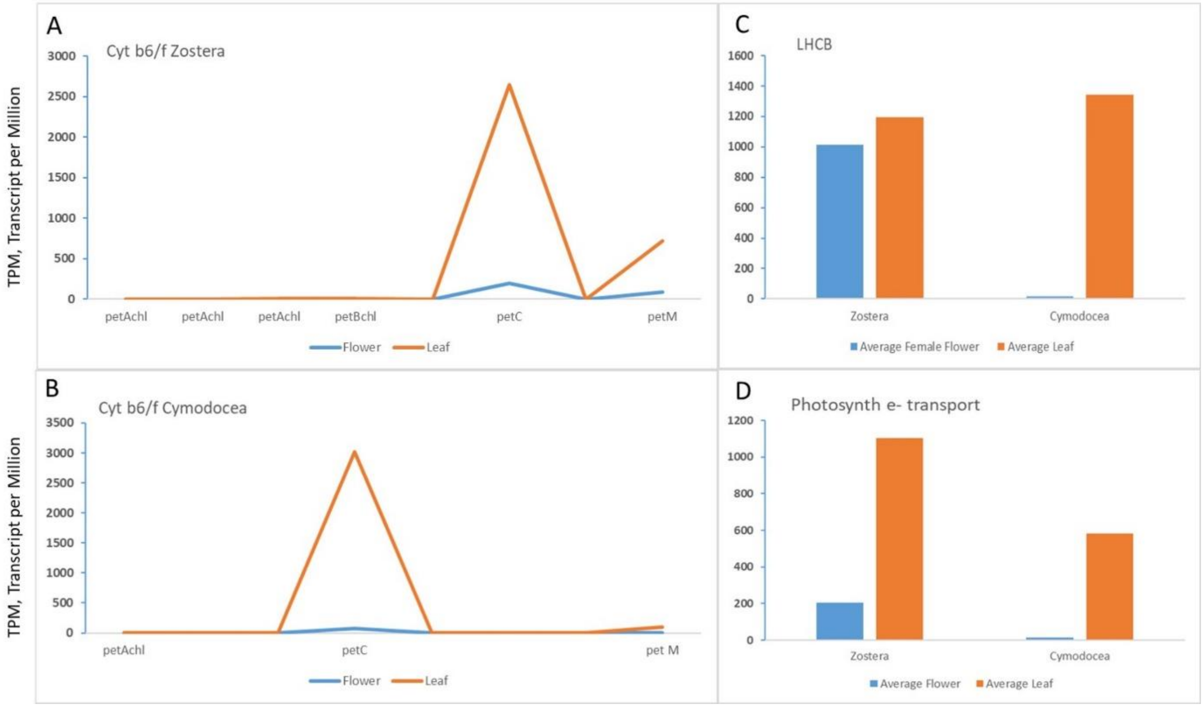

Supplementary Figure 5.6.3 Differential expression of Cyt b6/f complex, *LHCB* and electron transport genes in *Z. marina* and *C. nodosa*.

A, B: expression of nuclear copies of chloroplast genes (*chl*) and of nuclear genes in leaf and flower tissue. C, D: comparison between gene expression in leaf and flower tissue.

Supplementary Note 5.6.3 Light Signaling & Circadian Clock

Seagrasses mostly conserved the full repertoire of orthologous genes for photosensory proteins and signalling systems, evolved in the green lineage during the different stages of plant terrestrialization (Supplementary Figure 5.6.4) (Han et al. 2019; Jing and Lin 2020). Seagrasses conserved genes for all the three classes of photoreceptors UV-A/Blue, UV-B and RED/FAR-RED typical of higher plants, with few exceptions (Supplementary Figure 5.6.4) as well as at least one ortholog for each of the three main classes of UVA/Blue photoreceptors, that are Phototropins (*PHOT*), Cryptochromes (*CRY*) and the LOV/F-box protein (*FKF/LKP/ZTL*) (Supplementary Figure 5.6.4).

The UV-B receptor (UVB-Resistance 8) that is already known to be absent in *Z. marina* is still present in the other three species although the predicted protein sequence of *UVR8* in *P. oceanica* has a shorter N terminus compared to other species (data not shown) and lacks the C27 domain (Supplementary Figure 5.6.5). This is a region of 27 amino acids from the C terminus that mediates the interaction with proteins repressor of photomorphogenesis 1, 2 (*RUP1* and *RUP2*) (Yin et al. 2015), two proteins belonging to UV-B signalling including UV-B acclimation and tolerance. *RUP1* and *RUP2* are missing in the *Z. marina* genome (Supplementary Figure 5.6.4). These observations indicate that UV-B tolerance and the downstream regulation signalling pathways vary among species and are related to the relative light habitat features.

Red/far-red photoreceptors (Phytochromes) are present in a variety of organisms (Rockwell and Lagarias 2020). The phytochrome structure in plants is highly conserved, showing the same domain architecture in all members of the streptophyte (charophyte algae and land plants) PHY1/2 lineage, having originated in a common ancestor

(Li et al. 2015; Rockwell and Lagarias 2020). In some algal lineages, such as Zygnematales and Coleochaetales, phytochromes also show non-canonical forms (Li et al.2015). In seed plants, phytochromes underwent lineage-specific gene duplications, leading to three main forms (phyA, phyB, phyC), plus two additional forms (phyD and phyE), which are restricted to some taxa (Mathews 2010). The number of phytochromes varies among species: eudicots tend to have two or three phytochromes genes (up to five *PHYA-PHYE* in *A.thaliana*), while monocots tend to have a lower number of genes, generally only one gene for *PHYA* and one for *PHYB*. The transition to a submerged marine environment did not lead to a general reduction of phytochrome genes (Supplementary Figure 5.6.4); indeed, all the four species investigated possess at least one gene for phytochome A (*PHYA*) and phytochrome B (*PHYB*) while *P. oceanica* and *T. testudinum* also possess an orthologous gene for *PHYC*. Furthermore, *P. oceanica* and *C. nodosa* have a unique orthologous cluster (OG0026441) for a Phytochome E (*PHYE*) (Supplementary Figure 5.6.6) often absent in monocotyledonous plants (Smith 2000; Mathews 2006). After WDG and WGT, *P. oceanica* and *C. nodosa* as well as *P. acutifolius* retained duplicated genes for *CRY* or *PHOT*s (Supplementary Figure 5.6.4) while *P. oceanica* and *P. acutifolius* also for *ZTL/FKF1*.

Components of the downstream light signaling pathways such phytochrome interacting factors (*PIF*s), constitutive photomorphogenic protein 1 (*COP1*) and elongated hypocotyl 5 (*HY5*) are still present (Supplementary Figure 5.6.4) with several orthologous genes comparable with other aquatic and land species. Also, the repertoire of transcription factors essential for photomorphogenesis and seed emergence, like the far-red elongated hypocotyl 1,3 (*FHY1/3*), far-red-impaired response1 (*FAR1*) and long after far-red light 1 (*LAF1*) is the one typical of land angiosperms. However, further functional studies must investigate if those genes have also conserved the same pattern of expression of land plants, especially during critical stages of seed setting and plant development.

Perception of surrounding light cues is critical also for the entrainment of the circadian clock system. The circadian clock regulates a plethora of processes that affect physiology and life cycle in plants, such as daily water and carbon availability and hormone signalling pathways (McClung 2019). All seagrass species, apart from *T. testudinum*, lost ortholog genes for *Timing of Cab* (*TOC1*) (Supplementary Figure 5.6.4). *TOC1* is one of the key clockwork components of the evening transcriptional-translational loop (Harmer 2009) belonging to the PSEUDO RESPONSE REGULATOR (*PRR*) family with a crucial function in the integration of light signals to the circadian control (Pokhilko et al. 2013). *TOC1* has also a central role in adapting plant physiology to drought (Legnaioli et al. 2009; Wang et al. 2020) and in regulating the day-night energy metabolism (Cervela-Cardona et al. 2021). Remarkably, *TOC1* is also lost in the freshwater *P. acutifolius* and *W. australiana,* the latter showing a reduced circadian time control of gene expression in comparison with *Arabidopsis* (Michael et al. 2021). The loss of some genes related to the circadian system in a large part of marine and freshwater species can suggest that, in the aquatic environment, the absence of some environmental stressors typical of land habitats, such as water deficit, has led to a reduction of the regulative constraints for daily management of some metabolic and developmental plant processes. Further functional studies could highlight changes in regulative networks mediated by circadian clock genes and their implication for seagrass adaptation to marine environments.

C. nodosa, *P. oceanica* and *T. testidinum* retained, after WGT and WGD events, one gene each related to the circadian clock and photoperiodism, respectively *LNK1*, *ZTL* and *GI* (Supplementary Figure 5.6.4).

			Seagrasses				Freshwater species			Mangrove		Moconots					Eudicots								
	Orthogroup	Gene name	***CN***	***PO***	***TT***	***ZM***	***PA***	*SP*	*WA*	*AM*	*RA*	*OS*	*BD*	*AC*	*EG*	*AO*	*BV*	*UG*	*SL*	*CC*	*VV*	*PT*	*AT*	*TC*	*ATR*
Gene families containing photoreceptors	OG0007303	*PHYA*	1	1	1	1	1	1	1	2	1	1	2	1	1	1	1	2	1	1	1	1	1	1	1
	OG0003273	*PHYB/E*	1	1	1	1	1	1	1	2	3	1	1	1	2	1	1	4	3	1	2	2	3	2	1
	OG0026441	*PHYE*	1	1	0	0	0	0	0	0	0	0	0	0	0	0	0	0	0	0	0	0	0	0	0
	OG0011336	*PHYC*	0	1	1	0	1	1	1	2	1	1	1	1	1	1	1	0	1	1	1	0	1	1	0
	OG0008432	*UVR8*	1	1	1	0	0	1	1	2	1	2	2	1	1	2	1	1	1	1	1	1	1	1	1
	OG0004552	*CRY1*	2	1	1	1	1	1	1	3	2	2	2	1	2	1	1	1	2	1	1	2	1	1	1
	OG0006828	*CRY2*	1	2	2	0	1	1	2	2	1	1	1	2	1	1	1	1	1	1	1	1	1	1	1
	OG0010438	*CRY3*	1	1	1	0	1	1	1	0	1	1	1	1	1	2	1	0	1	2	1	1	1	1	1
	OG0001443	*PHOT1, PHOT2*	3	3	2	2	2	2	4	2	2	3	2	2	2	2	2	2	2	2	2	3	2	2	2
	OG0006742	*PAS/LOV*	1	1	1	1	1	1	1	2	1	1	1	2	2	1	1	0	2	1	1	2	1	1	1
	OG0003641	*ZTL, ADO1*	1	2	2	1	2	2	1	4	1	2	2	1	2	2	1	1	1	1	1	2	1	1	1
	OG0017621	*ADO2*	0	1	0	0	0	0	0	0	1	0	0	1	0	0	0	0	0	0	0	0	1	0	0
	OG0010351	*FKF1, ADO3*	1	1	1	0	2	1	2	1	1	1	1	0	1	0	1	0	1	1	1	2	1	1	1
	OG0006492	*RUP1, RUP2*	1	3	1	0	1	1	1	4	1	1	1	1	1	1	0	1	1	1	1	2	2	1	1
	OG0002079	*HY5*	3	2	1	1	2	3	2	3	2	2	3	3	3	2	1	1	1	1	2	3	2	1	2
	OG0003554	*COP1*	1	1	1	2	1	1	1	3	2	1	1	1	1	1	2	2	2	2	2	3	1	2	1
	OG0004587	*SPA1*	1	1	1	1	1	1	1	2	1	1	1	1	2	1	2	0	2	2	2	3	2	2	1
	OG0002845	*COR28*	2	2	2	1	2	0	2	2	2	3	2	2	3	1	1	2	1	1	3	2	2	1	1
	OG0008799	*CSU2*	1	1	1	1	1	1	1	1	1	1	1	1	1	1	1	1	1	2	1	1	1	1	1
	OG0010907	*COP10*	1	1	1	1	0	1	1	1	1	1	1	1	1	1	1	1	1	1	0	1	1	1	0
	OG0009317	*DET1*	1	1	1	1	0	1	1	1	1	1	1	1	2	1	1	1	1	1	1	1	1	1	1
	OG0004432	*DDB1B*	1	1	1	1	6	1	1	2	1	2	2	1	2	1	2	0	1	0	1	1	2	1	1
	OG0003768	*PIF3/15*	2	1	1	1	0	1	2	3	3	0	1	2	3	3	1	1	1	1	1	3	1	2	1
	OG0016987	*PIF-like 15*	1	0	0	1	0	0	0	0	0	0	0	0	0	0	0	0	0	0	0	0	0	0	0
	OG0009778	*PIF-like 15*	2	2	2	3	4	1	0	0	0	2	2	3	0	0	0	0	0	0	0	1	0	1	0
	OG0009607	*PIF-like 13*	2	2	0	0	1	2	1	0	0	4	2	1	0	2	1	1	0	0	1	0	3	0	1
	OG0004930	*PIF-like 13*	2	2	2	0	0	1	2	1	1	1	2	3	2	4	0	1	1	1	1	2	2	1	0
	OG0007588	*UNE10*	1	1	1	0	0	0	1	3	2	1	1	1	2	1	1	0	2	1	2	2	1	1	1
	OG0003522	*SRR1*	1	1	5	3	1	1	1	1	1	1	1	1	3	1	0	2	1	2	1	3	1	1	1
	OG0006894	*FHY1*	1	1	1	1	1	1	1	2	1	1	1	1	2	1	1	0	2	1	1	1	1	1	2
	OG0000045	*FAR1,FHY3*	8	8	7	6	6	11	6	10	19	22	17	1	0	1	10	0	16	0	9	26	12	17	0
	OG0003983	*FAR1-related*	1	2	1	1	1	2	1	1	2	1	1	0	0	3	2	0	2	0	3	4	1	2	3
	OG0000000	*LAF1 (MYB -releted)*	31	31	25	20	15	20	27	25	10	40	40	38	3	29	26	19	41	28	37	70	44	43	22
	OG0000541	*FT*	1	2	3	4	5	3	4	2	2	8	9	5	6	5	2	2	3	3	0	2	2	1	1
	OG0006964	*FRI*	0	1	0	0	0	2	1	1	1	1	1	1	1	1	0	2	6	1	2	2	1	1	1
	OG0002154	*FRI-like*	1	1	1	1	1	2	2	3	2	3	2	2	3	2	2	2	3	1	2	3	1	3	2
	OG0004911	*VRN5, VIL1*	1	1	1	1	1	1	1	3	2	2	2	1	1	1	1	2	1	1	2	2	1	1	1
Gene families involved in circadian clock toolkit genes	OG0003641	*ZTL, ADO1*	1	2	2	1	2	2	1	4	1	2	2	1	2	2	1	1	1	1	1	2	1	1	1
	OG0017621	*ADO2*	0	1	0	0	0	0	0	0	1	0	0	1	0	0	0	0	0	0	0	0	1	0	0
	OG0010351	*FKF1, ADO3*	1	1	1	0	2	1	2	1	1	1	1	0	1	0	1	0	1	1	1	2	1	1	1
	OG0003077	*LHY/ CCA1*	2	1	2	1	2	2	2	1	2	1	1	2	3	1	1	5	1	1	1	2	2	1	1
	OG0010029	*TOC1*	0	0	1	0	0	1	0	2	2	1	1	2	1	1	1	2	2	1	1	1	1	1	1
	OG0010705	*PRR2*	1	1	1	0	1	1	1	0	2	0	0	1	1	1	1	0	2	1	1	2	1	1	1
	OG0001611	*PRR9/5*	1	3	2	1	2	3	1	3	3	1	1	3	1	5	2	5	2	1	2	4	2	2	1
	OG0003230	*PRR7/3*	1	1	1	1	1	1	1	2	2	2	2	2	2	1	2	0	2	2	3	2	2	3	1
	OG0004910	*GI*	1	1	2	1	1	1	1	2	2	1	1	1	2	2	1	2	2	1	1	2	1	1	1
	OG0004704	*LUX*	2	2	3	1	0	1	2	1	1	1	1	1	1	0	2	2	2	1	1	2	2	1	1
	OG0004484	*CO*	1	1	2	0	0	1	1	2	2	2	2	2	2	1	1	0	3	1	1	2	3	2	1
	OG0000552	*ELF4*	1	2	1	2	1	1	2	5	4	3	3	6	5	0	3	6	6	3	3	7	5	4	2
	OG0003752	*LNK1*	2	2	1	0	1	0	2	1	5	1	1	2	4	1	1	3	2	1	2	1	1	1	1
	OG0022062	*LNK1*	0	0	0	1	1	0	0	0	0	0	0	0	0	0	0	0	0	0	0	0	0	0	0
	OG0003157	*LNK2*	1	1	2	0	2	1	1	2	2	1	1	1	3	5	1	1	0	4	1	7	1	1	0
	OG0011416	*LNK3/4*	0	1	0	0	1	1	1	3	0	0	0	1	0	0	1	0	2	0	1	2	2	1	1
	OG0002371	*TIC*	1	1	1	0	1	3	1	4	7	1	1	1	2	1	0	4	1	1	2	5	2	2	2

Supplementary Figure 5.6.4 Gene families containing photoreceptors and the main integration of light signalling toolkit genes. Species sequenced in this work are in bold. Full list of abbreviation of the species names used in the figure: CN *Cymodocea nodosa*; PO *Posidonia oceanica*; TT *Thalassia testudinum*; ZM *Zostera marina*; PA *Potamogeton acutifolius*; SP *Spirodela polyrhiza*; WA *Wolffia australiana*; AM *Avicennia marina*; RA *Rhizophora apiculate*; OS *Oryza sativa*; BD *Brachypodium distachyon*; AC *Ananas comosus*; EG *Elaeis guineensis*; AO *Asparagus officinalis*; BV *Beta vulgaris*; UG *Utricularia gibba*; SL *Solanum lycopersicum*; CC *Coffea canephora*; VV *Vitis vinifera*; PT *Populus trichocarpa*; AT Arabidopsis thaliana; TC *Theobroma cacao*; ATR *Amborella trichopoda*.

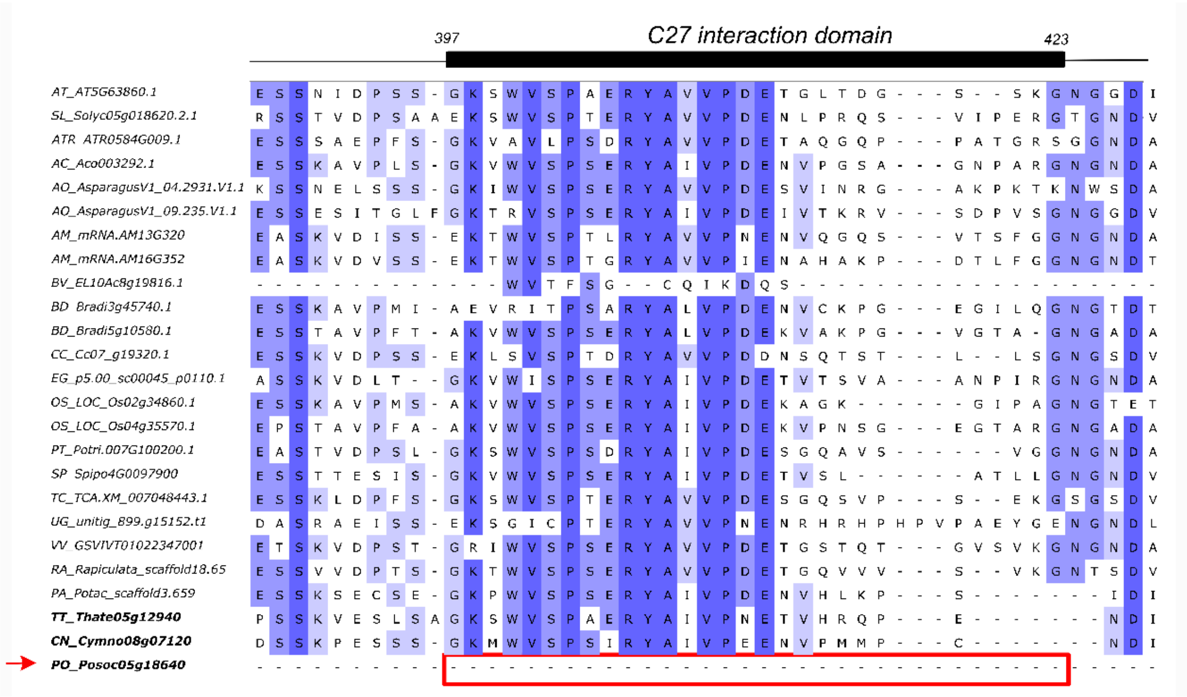

Supplementary Figure 5.6.5 The N terminus alignment of UVB-Resistance 8.

For the alignment, proteins sequences of the orthogroup OG0008432 were used. Sequences of seagrasses are in bold. Full list of abbreviation of the species names used in the figure: AT *Arabidopsis thaliana*; SL *Solanum lycopersicum*; ATR *Amborella trichopoda*; AC *Ananas comosus*; AO *Asparagus officinalis*; AM *Avicennia marina*; BV *Beta vulgaris*; BD *Brachypodium distachyon*; CC *Coffea canephora*; CN *Cymodocea nodosa*; EG *Elaeis guineensis*; OS *Oryza sativa*; PO *Posidonia oceanica*; PT *Populus trichocarpa*; PA *Potamogeton acutifolius*; RA *Rhizophora apiculate*; SP *Spirodela polyrhiza*; TT *Thalassia testudinum*; TC *Theobroma cacao*; VV *Vitis vinifera*; UG *Utricularia gibba*.

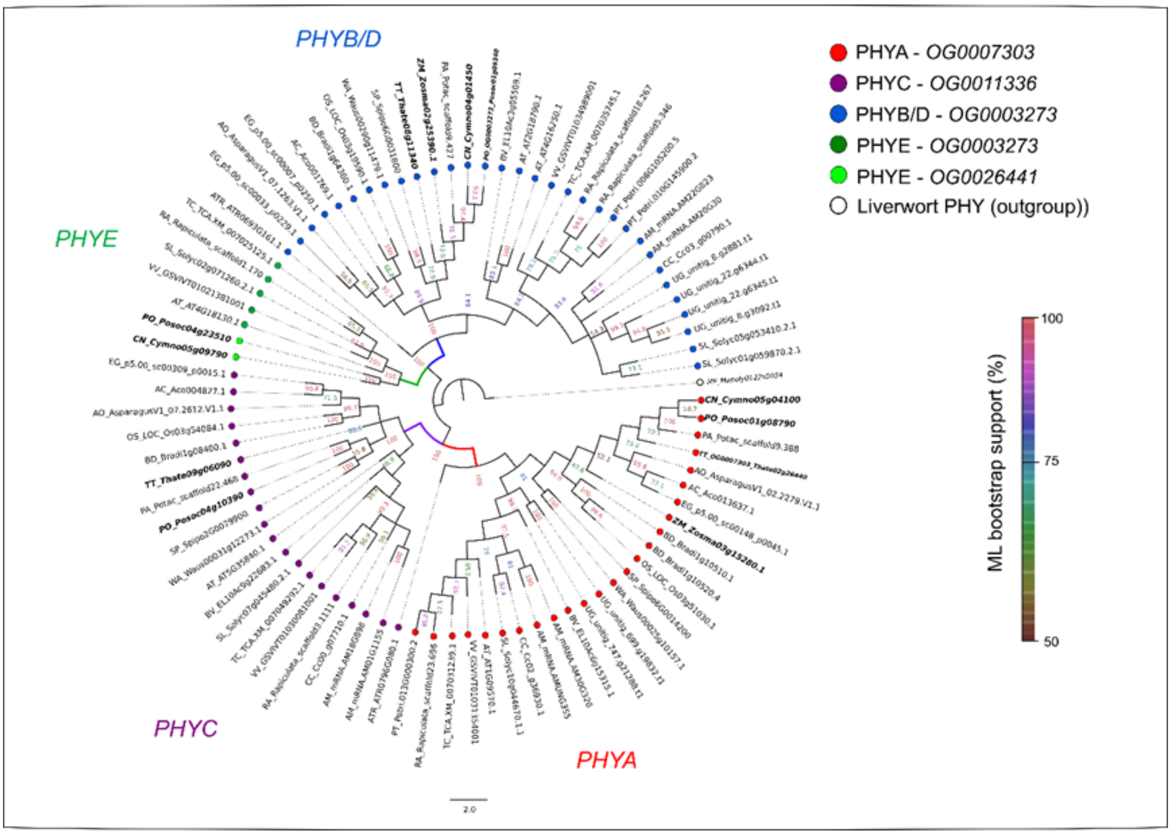

Supplementary Figure 5.6.6 Phylogenetic tree of phytochromes obtained from the 84 proteins sequences included in the orthogroups OG0007303, OG0003273, OG0011336 and OG0026441.

The 84 protein sequences of the four OGs (OG0007303, OG0003273, OG0011336 and OG0026441), which were functionally annotated as putative phytochromes, were aligned using Muscle (MEGA5), with the phytochrome sequence of the liverwort *Marchantia polymorpha* (MP_Mapoly0122s0054 white dot; selected from PLAZA 5.0 (ORTHO05M001074 https://bioinformatics.psb.ugent.be/plaza/versions/plaza_v5_monocots/) as an outgroup. The resulting multiple sequence alignment was trimmed with TRIMAL using the Automated 1 method. The ML phylogenetic tree was generated using the JTT + I +G model with MEGA5. Bootstrap values (expressed as percentages) were calculated over 1,000 replications. Only branches with a bootstrap support over 52% are shown. Protein sequences of seagrasses are indicated in bold, while *Arabidopsis thaliana* orthologs of PHYA-E were in italic (i.e., AT_AT1G09570.1 phyA, AT_AT5G35840.1 phyC, AT_AT2G18790.1 phyB, AT_AT4G16250.1 phyD, AT_AT4G18130.1 phyE; according to TAIR classification). PHYA (red branch) and PHYC (violet branch) were encoded by proteins sequences which were included respectively in the OG0007303 (red dots) and OG0011336 (violet dots). PHYB/D (blue branch) groups proteins sequences of OG0003273 (blue dots) while the PHYE form (green branch) is represented by five proteins included in the OG0003273 (dark-green dots) plus two proteins of Orthogroup OG0026441 (light-green dots) which is an OG exclusive of seagrasses, suggesting that the two seagrasses *P. oceanica* and *C. nodosa* have retained a gene copy of PHYE even if this phytochrome form is often missing in other monocotyledonous plants (Mathews 2006). Complete list of species names' abbreviations used in the figure: AM *Avicennia marina*; AC *Ananas comosus*; AT *Arabidopsis thaliana*; AO *Asparagus officinalis*; ATR *Amborella trichopoda*; BV *Beta vulgaris*; BD *Brachypodium distachyon*; CC *Coffea canephora*; CN *Cymodocea nodosa*; EG *Elaeis guineensis*; OS *Oryza sativa*; PO *Posidonia oceanica*; PT *Populus trichocarpa*; PA *Potamogeton acutifolius*; RA Rhizophora apiculata; SL *Solanum lycopersicum*; SP *Spirodela polyrhiza*; TT *Thalassia testudinum*; TC *Theobroma cacao*; VV *Vitis vinifera*; UG *Utricularia gibba*.

5.7 NAC transcriptional factors

Supplementary Note 5.7 NAC transcriptional factors

NAC proteins (NAM, ATAF1–2 and CUC2 transcription factors) are one of the largest family of transcriptional factors that are involved in different developmental processes as well as in the regulation of signaling pathways in response to abiotic stressors, especially salt stress (Puranik et al. 2012). A comparable number of sequences were found in seagrasses with respect to land plants, freshwater and mongroves species. However, specific orthogroups were found for seagrasses. One of them is annotated as JUNGBRUNNEN 1 (JUB1), and a specific functional analysis revealed that while *P. oceanica* retained JUNGBRUNNEN 1 (*JUB1*) genes with low expression values, *C. nodosa* expressed *JUB1* genes in leaves. *JUB1* is a central longevity regulator as well as a regulator of responses to abiotic stressors enhancing salt stress tolerance (Wu et al., 2012) regulating plant responses to environmental factors.

In addition, other sequences annotated as *JUB1* were found across all species belonging to different orthogroups. The OG0000816 was the most representative orthogroup counting a total of 67 sequences across all species (land plants, freshwater and mongroves species). Here too, in *P. oceanica* only Posoc08g09120 and Posoc08g09110 were weakly expressed in leaves. Contrarily, sequences found for the other seagrasses (Cymno02g16010, Cymno02g16050, Zosma03g24220.1) showed higher expression values especially in leaves. In *T. testudinum*, one single gene copy was found (Thate04g03560) specifically expressed in root and leaf. Thus, a phylogenetic tree was built including these sequences to visualize relationships of the *JUB1* sequences between seagrasses (Supplementary Figure 5.7). Sequences of *P. oceanica* (Posoc08g09120 and Posoc08g09110) and *C. nodosa* (Cymno02g16010, Cymno02g16050) belonging to the same orthogroup (OG0000816) formed a single clade, apart from *T. testudinum* (Thate04g03560), *Z. marina* (Zosma03g24220.1) and Posoc10g09490. Considering that *P. oceanica* and *C. nodosa* are phylogenetically closely related, the

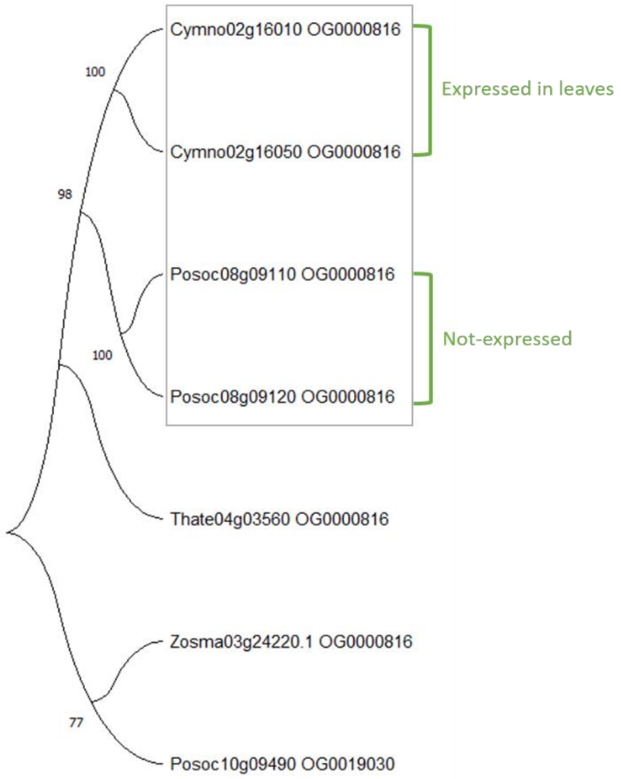

Supplementary Figure 5.7 Evolutionary analysis by Maximum Likelihood method of *JUB1* in seagrasses.

different expression levels observed for JUB1 sequences could suggests a functional re-organization that could be related to the different ecological requirements of these species, modulating stress tolerance in seagrasses, including response to salinity. *P. oceanica*, in fact, colonizes open coastal habitats with a very narrow range of salinity, contrary to *C. nodosa* which can also be present in estuarine dynamic environmental conditions and highly variable salinities.

5.8 Nitrogen metabolism

Supplementary Note 5.8 Nitrogen metabolism

Seagrass meadows act as an important nitrogen (N) filter in the coastal environments. In this context, seagrasses assimilate large amounts of N, and exude oxygen and labile carbon into sediments, which stimulate other processes in the nitrogen metabolism pathway, including nitrification-denitrification process that counterbalances the net N loads through microbial transformation (Zarnoch et al. 2017; Aoki et al. 2020). The key genes linked to nitrogen uptake/transport and assimilation were retained in all of the plants examined (Extended table 10). This corresponds to at least 66 and 25 orthogroups which function in uptake/transport and assimilation, respectively. Their existence is essential because an efficient N metabolism process is required to ensure normal growth and development in plants, regardless of their diversity, habitat or nature. Moreover, seagrasses may have acquired a more effective nitrogen metabolism in N-deficient marine environments through symbiotic N2-fixing bacteria, that could have facilitated the migration of flowering plants back to the sea some 100 million years ago (Mohr et al. 2021).

As compared to non-seagrass genomes, the nitrate transporter (*NRT*) gene families of seagrasses were contracted (40.71%), indicating that seagrasses may have evolved alternative mechanisms to utilize nitrogen sources more effectively (Extended table 10). Other than nitrate, seagrasses rely on ammonium as a primary source of nitrogen (Touchette and Burkholder 2001; Xu et al. 2020), particularly when exposed to anoxic conditions in marine sediment where nitrate is scarce due to disrupted ammonium-to-nitrate oxidation. In this case, ammonium is metabolized directly via GOGAT pathway, which catalyzes the formation of glutamine from glutamate and ammonium, instead of converting nitrate to ammonium prior to glutamine formation (Wang et al. 2021).

Nitrate reductase (*NR*) was expressed in all parts of seagrasses (flower, root, vegetative, rhizome), with *NR* robustly expressed in the root of *Zostera marina* and the leaves of *Cymodocea nodosa* and *Thalassia testudinum*. *NR* activity is widely influenced by light (Touchette and Burkholder 2001) and given that *NR* activity is highest during photosynthetic periods and lowest in the dark, nitrite reduction occurs more frequently in leaf tissues than in root tissues in most seagrasses, suggesting the importance of light on *NR* response (Manassa et al. 2017; Wang et al. 2021; Jiménez-Ramos et al. 2022). *Zostera marina* is unique from the other seagrasses as its *NR* activity can be maintained in the dark, provided that the environment is nitrate-enriched and tissue carbohydrate levels are high (Touchette and Burkholder 2001). The intensity and duration of *NR* activity is directly parallel with the soluble carbohydrate supplies (Touchette and Burkholder 2007). On the other hand, seagrass leaves such as those in *T. testudinum* tend to have higher efficiency of nitrogen assimilation compared to root, given that the NH_4^+ in the water column is relatively lower than the sediments where seagrass inhabit (Lee and Dunton 1999; Cornelisen and Thomas 2004).

5.9 Flower and pollen development

Supplementary Note 5.9 Flower and pollen development

MADS-box genes encode transcription factors that play a crucial role in controlling various developmental programs including the development of floral organs. We predicted considerable re-arrangements in seagrasses as petals and sepals are completely reduced. Type II MADS-box genes have been extensively studied for their role in specifying floral organ development.

To identify MADS-box genes in the genomes of *Z. marina, C. nodosa, P. oceanica, T. testudinum*, and *P. acutifolius*, we employed a hidden Markov model (HMM). The HMM profile for the SRF-TF domain (PF00319) was obtained from the Pfam database (Mistry et al. 2021). This profile was used to search against the local protein database using the HMMER software, with an E-value threshold of < 1e-5. Using the same method described above, MADS-box genes of *A. thaliana* and *O. sativa* were obtained. Subsequently, all candidate proteins from the seven species mentioned above were aligned using MAFFT, resulting in a concatenated dataset, which was then used for phylogenetic analysis to further identify the type II MADS-box genes. We identified 22, 26, 29, 24, and 29 Type II MADS-box genes in *Z. marina*, *P. oceanica*, *C. nodosa*, *T. testudium*, and *Potamogeton*, respectively (Supplementary Table 5.9 and Figure 4a). Among these, several are homologues of genes defining the well-known ABCE model (Lohmann and Weigel 2002; Krizek and Fletcher 2005): AP1 and AGL6 (A function for sepals and petals), PI and AP3 (B function for petals and stamen), as well as OsMADS32 (B function in rice), AG (C function for stamen and carpel), and SEP (E function for interacting with ABC function proteins).

We also analyzed the expression profile of these genes in various tissues. For the flower tissue of *Posidonia oceanica*, RNA-seq data was obtained from the NCBI Short Read Archive under BioProject ID PRJNA375717 (Entrambasaguas et al. 2017). Subsequently, the data was aligned to the *P. oceanica* assembly.

Supplementary Table 5.9. The MADS-box genes in seagrasses and *P. acutifolius*

		Z . marina	P. oceanica	C. nodosa	T. testudinum	P. acutifolius
MADS-box genes in total		48	46	44	34	38
Type II		22	26	29	24	29
A function	AP1	4	2	4	2	3
	AGL6	1	1	1	1	1
B function	PI	1	1	1	6	1
	AP3	1	1	1	1	1
	OsMADS32	1	1	0	1	1
C function	AG	4	3	6	1	3
E function	SEP	2	2	2	2	2

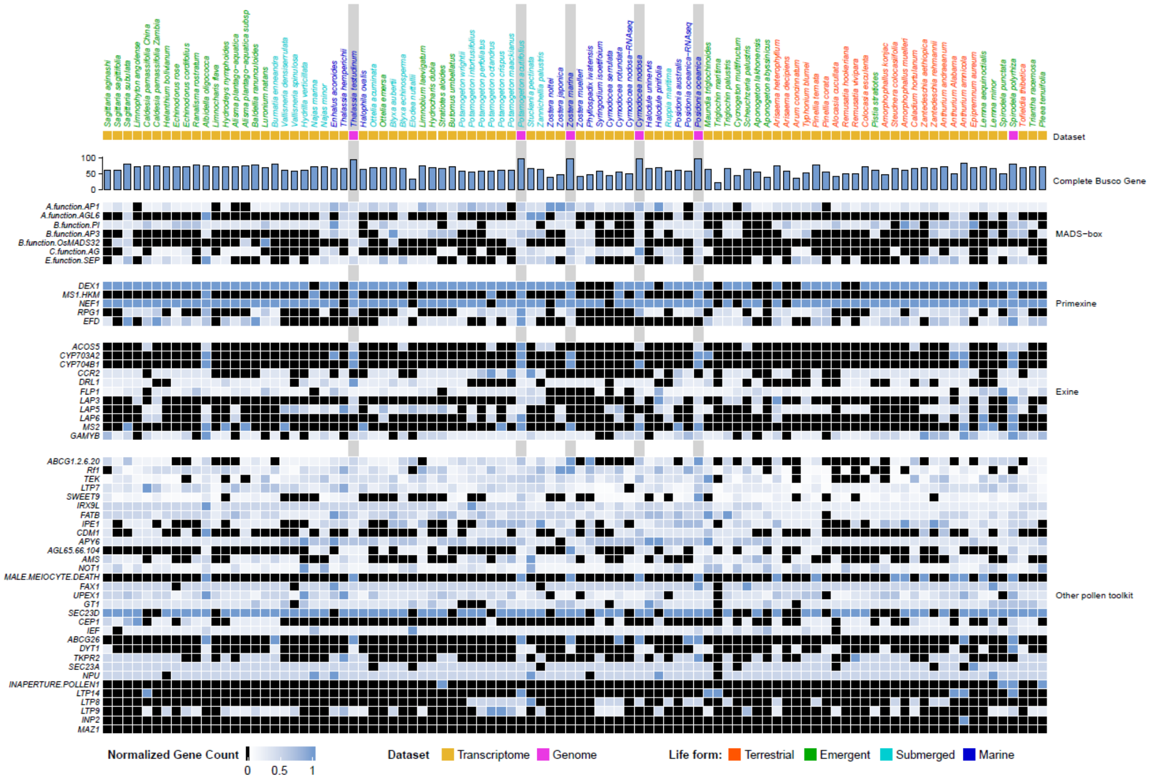

Supplementary Figure 5.9 Normalized gene copy numbers for flower and pollen development genes and gene families for 96 species, including 6 genomic data and 90 transcriptomic data. The light grey background denotes our genomic data of four seagrasses and one freshwater relative, *Potamogeton acutifolius*. Others are the transcriptomic data from Chen et al. (2022) and *Spirodela polyrhiza* genomic data. Normalization for each gene family was obtained by dividing the number of genes in that gene family for a particular species by the largest gene copy number within that family (considering all species). Genes in black are absent.

References

Altschul SF, Gish W, Miller W, Myers EW, Lipman DJ. 1990. Basic local alignment search tool. *J Mol Biol* **215**: 403-410.

An D, Zhou Y, Li C, Xiao Q, Wang T, Zhang Y, Wu Y, Li Y, Chao DY, Messing J et al. 2019. Plant evolution and environmental adaptation unveiled by long-read whole-genome sequencing of Spirodela. *Proc Natl Acad Sci U S A* **116**: 18893-18899.

Aoki LR, McGlathery KJ, Oreska MPJ. 2020. Seagrass restoration reestablishes the coastal nitrogen filter through enhanced burial. *Limnology and Oceanography* **65**: 1-12.

Berry EW. 1914. The Upper Cretaceous and Eocene floras of South Carolina and Georgia. doi:10.3133/pp84.

Campbell JE, Fourqurean JW. 2013. Mechanisms of bicarbonate use influence the photosynthetic carbon dioxide sensitivity of tropical seagrasses. *Limnology and Oceanography* **58**: 839-848.

Capella-Gutiérrez S, Silla-Martínez JM, Gabaldón T. 2009. trimAl: a tool for automated alignment trimming in large-scale phylogenetic analyses. *Bioinformatics* **25**: 1972-1973.

Capó-Bauçà S, Iñiguez C, Aguiló-Nicolau P, Galmés J. 2022. Correlative adaptation between Rubisco and CO2-concentrating mechanisms in seagrasses. *Nature Plants* **8**: 706-716.

Cervela-Cardona L, Yoshida T, Zhang Y, Okada M, Fernie A, Mas P. 2021. Circadian Control of Metabolism by the Clock Component TOC1. *Frontiers in Plant Science* **12**.

Chen C, Chen H, Zhang Y, Thomas HR, Frank MH, He Y, Xia R. 2020. TBtools: An Integrative Toolkit Developed for Interactive Analyses of Big Biological Data. *Mol Plant* **13**: 1194-1202.

Chen L-Y, Lu B, Morales-Briones DF, Moody ML, Liu F, Hu G-W, Huang C-H, Chen J-M, Wang Q-F. 2022. Phylogenomic Analyses of Alismatales Shed Light into Adaptations to Aquatic Environments. *Molecular Biology and Evolution* **39**.

Cornelisen CD, Thomas FIM. 2004. Ammonium and nitrate uptake by leaves of the seagrass Thalassia testudinum: impact of hydrodynamic regime and epiphyte cover on uptake rates. *Journal of Marine Systems* **49**: 177-194.

Crepet W, Nixon K. 1998. Fossil Clusiaceae from the late Cretaceous (Turonian) of New Jersey and implications regarding the history of bee pollination. *Am J Bot* **85**: 1122.

Dierckxsens N, Mardulyn P, Smits G. 2017. NOVOPlasty: de novo assembly of organelle genomes from whole genome data. *Nucleic Acids Res* **45**: e18.

DiMario RJ, Clayton H, Mukherjee A, Ludwig M, Moroney JV. 2017. Plant Carbonic Anhydrases: Structures, Locations, Evolution, and Physiological Roles. *Molecular Plant* **10**: 30-46.

Ding Y, Liu N, Virlouvet L, Riethoven J-J, Fromm M, Avramova Z. 2013. Four distinct types of dehydration stress memory genes in Arabidopsis thaliana. *BMC Plant Biology* **13**: 229.

Dongen SV. 2008. Graph Clustering Via a Discrete Uncoupling Process. *SIAM Journal on Matrix Analysis and Applications* **30**: 121-141.

Doyle JA, Endress PK, Upchurch GR. 2008. Early Cretaceous monocots: a phylogenetic evaluation. *Sborník Národního muzea v Praze Acta Musei nationalis Pragae* **64**: 61-87.

Edgar RC. 2004. MUSCLE: multiple sequence alignment with high accuracy and high throughput. *Nucleic Acids Res* **32**: 1792-1797.

Emms DM, Kelly S. 2015. OrthoFinder: solving fundamental biases in whole genome comparisons dramatically improves orthogroup inference accuracy. *Genome Biology* **16**: 157.

Emms DM, Kelly S. 2019. OrthoFinder: phylogenetic orthology inference for comparative genomics. *Genome Biol* **20**: 238.

Entrambasaguas L, Jahnke M, Biffali E, Borra M, Sanges R, Marin-Guirao L, Procaccini G. 2017. Tissue-specific transcriptomic profiling provides new insights into the reproductive ecology and biology of the iconic seagrass species Posidonia oceanica. *Mar Genomics* **35**: 51-61.

Gandolfo M, Nixon K, Crepet W. 1998. A new fossil flower from the Turonian of New Jersey: Dressiantha bicarpellata gen. et sp. nov. (Capparales). *Am J Bot* **85**: 964.

Guo L, Winzer T, Yang X, Li Y, Ning Z, He Z, Teodor R, Lu Y, Bowser TA, Graham IA et al. 2018. The opium poppy genome and morphinan production. *Science* **362**: 343-347.

Guo M, Liu JH, Ma X, Luo DX, Gong ZH, Lu MH. 2016. The Plant Heat Stress Transcription Factors (HSFs): Structure, Regulation, and Function in Response to Abiotic Stresses. *Front Plant Sci* **7**: 114.

Han X, Chang X, Zhang Z, Chen H, He H, Zhong B, Deng XW. 2019. Origin and Evolution of Core Components Responsible for Monitoring Light Environment Changes during Plant Terrestrialization. *Molecular Plant* **12**: 847-862.

Harmer SL. 2009. The circadian system in higher plants. *Annual review of plant biology* **60**: 357-377.

Heerklotz D, Döring P, Bonzelius F, Winkelhaus S, Nover L. 2001. The balance of nuclear import and export determines the intracellular distribution and function of tomato heat stress transcription factor HsfA2. *Mol Cell Biol* **21**: 1759-1768.

Huelsenbeck JP, Ronquist F. 2001. MRBAYES: Bayesian inference of phylogenetic trees. *Bioinformatics* **17**: 754-755.

Ikeda M, Mitsuda N, Ohme-Takagi M. 2011. Arabidopsis HsfB1 and HsfB2b act as repressors of the expression of heat-inducible Hsfs but positively regulate the acquired thermotolerance. *Plant Physiol* **157**: 1243-1254.

Iles WJ, Smith SY, Gandolfo MA, Graham SW. 2015. Monocot fossils suitable for molecular dating analyses. *Botanical Journal of the Linnean Society* **178**: 346-374.

International Peach Genome I, Verde I, Abbott AG, Scalabrin S, Jung S, Shu S, Marroni F, Zhebentyayeva T, Dettori MT, Grimwood J et al. 2013. The high-quality draft genome of peach (Prunus persica) identifies unique patterns of genetic diversity, domestication and genome evolution. *Nat Genet* **45**: 487-494.

Jacob F, Vernaldi S, Maekawa T. 2013. Evolution and Conservation of Plant NLR Functions. *Front Immunol* **4**: 297.

Janssen T, Bremer K. 2004. The age of major monocot groups inferred from 800+ rbcL sequences. *Botanical Journal of the Linnean Society* **146**: 385-398.

Jiménez-Ramos R, Villazán B, Egea LG, Cantero R, Pérez-Lloréns JL, Vergara JJ, Brun FG. 2022. Differential ecophysiological responses to inorganic nitrogen sources (ammonium versus nitrate) and light levels in the seagrass Zostera noltei. *Marine Ecology Progress Series* **702**: 57-70.

Jing Y, Lin R. 2020. Transcriptional Regulatory Network of the Light Signaling Pathways. *The New phytologist*.

Koch M, Bowes G, Ross C, Zhang X-H. 2013. Climate change and ocean acidification effects on seagrasses and marine macroalgae. *Global Change Biology* **19**: 103-132.

Kozik A, Rowan BA, Lavelle D, Berke L, Schranz ME, Michelmore RW, Christensen AC. 2019. The alternative reality of plant mitochondrial DNA: One ring does not rule them all. *PLoS Genet* **15**: e1008373.

Krizek BA, Fletcher JC. 2005. Molecular mechanisms of flower development: an armchair guide. *Nature Reviews Genetics* **6**: 688-698.

Krzywinski M, Schein J, Birol I, Connors J, Gascoyne R, Horsman D, Jones SJ, Marra MA. 2009. Circos: an information aesthetic for comparative genomics. *Genome Res* **19**: 1639-1645.

Larkum AWD, Davey PA, Kuo J, Ralph PJ, Raven JA. 2017. Carbon-concentrating mechanisms in seagrasses. *Journal of Experimental Botany* **68**: 3773-3784.

Larkum AWD, Pernice M, Schliep M, Davey P, Szabo M, Raven JA, Lichtenberg M, Brodersen KE, Ralph PJ. 2018. Photosynthesis and Metabolism of Seagrasses. In *Seagrasses of Australia: Structure, Ecology and Conservation*, doi:10.1007/978-3-319-71354-0_11 (ed. AWD Larkum, et al.), pp. 315-342. Springer International Publishing, Cham.

Laslett D, Canback B. 2004. ARAGORN, a program to detect tRNA genes and tmRNA genes in nucleotide sequences. *Nucleic Acids Res* **32**: 11-16.

Lee BH, Henderson DA, Zhu JK. 2005. The Arabidopsis cold-responsive transcriptome and its regulation by ICE1. *Plant Cell* **17**: 3155-3175.

Lee K-S, Dunton KH. 1999. Inorganic nitrogen acquisition in the seagrass Thalassia testudinum: Development of a whole-plant nitrogen budget. *Limnology and Oceanography* **44**: 1204-1215.

Legnaioli T, Cuevas J, Mas P. 2009. TOC1 functions as a molecular switch connecting the circadian clock with plant responses to drought. *The EMBO Journal* **28**: 3745-3757.

Les DH, Cleland MA, Waycott M. 1997. Phylogenetic Studies in Alismatidae, II: Evolution of Marine Angiosperms (Seagrasses) and Hydrophily. *Systematic Botany* **22**: 443.

Li F-W, Melkonian M, Rothfels CJ, Villarreal JC, Stevenson DW, Graham SW, Wong GK-S, Pryer KM, Mathews S. 2015. Phytochrome diversity in green plants and the origin of canonical plant phytochromes. *Nature Communications* **6**: 7852.

Liu Y, Zeng Z, Zhang YM, Li Q, Jiang XM, Jiang Z, Tang JH, Chen D, Wang Q, Chen JQ et al. 2021. An angiosperm NLR Atlas reveals that NLR gene reduction is associated with ecological specialization and signal transduction component deletion. *Mol Plant* **14**: 2015-2031.

Lohmann JU, Weigel D. 2002. Building beauty: the genetic control of floral patterning. *Dev Cell* **2**: 135-142.

Loytynoja A, Goldman N. 2005. An algorithm for progressive multiple alignment of sequences with insertions. *Proc Natl Acad Sci U S A* **102**: 10557-10562.

Ma X, Fan J, Wu Y, Zhao S, Zheng X, Sun C, Tan L. 2020. Whole-genome de novo assemblies reveal extensive structural variations and dynamic organelle-to-nucleus DNA transfers in African and Asian rice. *Plant J* **104**: 596-612.

Ma X, Olsen JL, Reusch TBH, Procaccini G, Kudrna D, Williams M, Grimwood J, Rajasekar S, Jenkins J, Schmutz J et al. 2021a. Improved chromosome-level genome assembly and annotation of the seagrass, Zostera marina (eelgrass). *F1000Research* **10**.

Ma X, Olsen JL, Reusch TBH, Procaccini G, Kudrna D, Williams M, Grimwood J, Rajasekar S, Jenkins J, Schmutz J et al. 2021b. Improved chromosome-level genome assembly and annotation of the seagrass, Zostera marina (eelgrass). *F1000Res* **10**: 289.

Manassa RP, Smith TM, Beardall J, Keough MJ, Cook PLM. 2017. Capacity of a temperate intertidal seagrass species to tolerate changing environmental conditions: Significance of light and tidal exposure. *Ecological Indicators* **81**: 578-586.

Marina K, Thorsten BHR, Tal D. 2023. Worldwide population genomics reveal long-term stability of the mitochondrial chromosome composition in a keystone marine plant. *bioRxiv* doi:10.1101/2023.04.21.537793: 2023.2004.2021.537793.

Mathews S. 2006. Phytochrome-mediated development in land plants: red light sensing evolves to meet the challenges of changing light environments. *Molecular ecology* **15**: 3483-3503.

Mathews S. 2010. Evolutionary Studies Illuminate the Structural-Functional Model of Plant Phytochromes. *The Plant Cell* **22**: 4-16.

McClung CR. 2019. The plant circadian oscillator. Vol 8.

Michael TP, Ernst E, Hartwick N, Chu P, Bryant D, Gilbert S, Ortleb S, Baggs EL, Sree KS, Appenroth KJ et al. 2021. Genome and time-of-day transcriptome of Wolffia australiana link morphological minimization with gene loss and less growth control. *Genome Research* **31**: 225-238.

Michalovova M, Vyskot B, Kejnovsky E. 2013. Analysis of plastid and mitochondrial DNA insertions in the nucleus (NUPTs and NUMTs) of six plant species: size, relative age and chromosomal localization. *Heredity* **111**: 314-320.

Ming R, VanBuren R, Wai CM, Tang H, Schatz MC, Bowers JE, Lyons E, Wang ML, Chen J, Biggers E et al. 2015. The pineapple genome and the evolution of CAM photosynthesis. *Nat Genet* **47**: 1435-1442.

Minh BQ, Schmidt HA, Chernomor O, Schrempf D, Woodhams MD, von Haeseler A, Lanfear R. 2020. IQ-TREE 2: New Models and Efficient Methods for Phylogenetic Inference in the Genomic Era. *Molecular Biology and Evolution* **37**: 1530-1534.

Mishra SK, Tripp J, Winkelhaus S, Tschiersch B, Theres K, Nover L, Scharf KD. 2002. In the complex family of heat stress transcription factors, HsfA1 has a unique role as master regulator of thermotolerance in tomato. *Genes Dev* **16**: 1555-1567.

Mistry J, Chuguransky S, Williams L, Qureshi M, Salazar GA, Sonnhammer ELL, Tosatto SCE, Paladin L, Raj S, Richardson LJ et al. 2021. Pfam: The protein families database in 2021. *Nucleic Acids Res* **49**: D412-d419.

Mittal D, Madhyastha DA, Grover A. 2012. Gene expression analysis in response to low and high temperature and oxidative stresses in rice: combination of stresses evokes different transcriptional changes as against stresses applied individually. *Plant Sci* **197**: 102-113.

Mohr W, Lehnen N, Ahmerkamp S, Marchant HK, Graf JS, Tschitschko B, Yilmaz P, Littmann S, Gruber-Vodicka H, Leisch N et al. 2021. Terrestrial-type nitrogen-fixing symbiosis between seagrass and a marine bacterium. *Nature* **600**: 105-109.

Morley SA, Nielsen BL. 2017. Plant mitochondrial DNA. *Front Biosci (Landmark Ed)* **22**: 1023-1032.

Murat F, Armero A, Pont C, Klopp C, Salse J. 2017. Reconstructing the genome of the most recent common ancestor of flowering plants. *Nature Genetics* **49**: 490-496.

Nauheimer L, Metzler D, Renner SS. 2012. Global history of the ancient monocot family Araceae inferred with models accounting for past continental positions and previous ranges based on fossils. *New Phytologist* **195**: 938-950.

Olsen JL, Rouze P, Verhelst B, Lin YC, Bayer T, Collen J, Dattolo E, De Paoli E, Dittami S, Maumus F et al. 2016. The genome of the seagrass Zostera marina reveals angiosperm adaptation to the sea. *Nature* **530**: 331-335.

Personat JM, Tejedor-Cano J, Prieto-Dapena P, Almoguera C, Jordano J. 2014. Co-overexpression of two Heat Shock Factors results in enhanced seed longevity and in synergistic effects on seedling tolerance to severe dehydration and oxidative stress. *BMC Plant Biol* **14**: 56.

Petersen G, Cuenca A, Zervas A, Ross GT, Graham SW, Barrett CF, Davis JI, Seberg O. 2017. Mitochondrial genome evolution in Alismatales: Size reduction and extensive loss of ribosomal protein genes. *PLoS One* **12**: e0177606.

Pokhilko A, Mas P, Millar AJ. 2013. Modelling the widespread effects of TOC1 signalling on the plant circadian clock and its outputs. *BMC Systems Biology* **7**: 23-23.

Price MN, Dehal PS, Arkin AP. 2010. FastTree 2--approximately maximum-likelihood trees for large alignments. *PLoS One* **5**: e9490.

Puranik S, Sahu PP, Srivastava PS, Prasad M. 2012. NAC proteins: regulation and role in stress tolerance. *Trends in Plant Science* **17**: 369-381.

Rao SK, Magnin NlC, Reiskind JB, Bowes G. 2002. Photosynthetic and Other Phosphoenolpyruvate Carboxylase Isoforms in the Single-Cell, Facultative C4System of Hydrilla verticillata *Plant Physiology* **130**: 876-886.

Rockwell NC, Lagarias JC. 2020. Phytochrome evolution in 3D: deletion, duplication, and diversification. *New Phytologist* **225**: 2283-2300.

Ross TG, Barrett CF, Soto Gomez M, Lam VKY, Henriquez CL, Les DH, Davis JI, Cuenca A, Petersen G, Seberg O et al. 2016. Plastid phylogenomics and molecular evolution of Alismatales. *Cladistics* **32**: 160-178.

Rozewicki J, Li S, Amada KM, Standley DM, Katoh K. 2019. MAFFT-DASH: integrated protein sequence and structural alignment. *Nucleic Acids Research* **47**: W5-W10.

Rubio L, García D, García-Sánchez MJ, Niell FX, Felle HH, Fernández JA. 2017. Direct uptake of HCO3− in the marine angiosperm Posidonia oceanica (L.) Delile driven by a plasma membrane H+ economy. *Plant, Cell & Environment* **40**: 2820-2830.

Sakuma Y, Maruyama K, Qin F, Osakabe Y, Shinozaki K, Yamaguchi-Shinozaki K. 2006. Dual function of an Arabidopsis transcription factor DREB2A in water-stress-responsive and heat-stress-responsive gene expression. *Proc Natl Acad Sci U S A* **103**: 18822-18827.

Scharf KD, Berberich T, Ebersberger I, Nover L. 2012. The plant heat stress transcription factor (Hsf) family: structure, function and evolution. *Biochim Biophys Acta* **1819**: 104-119.

Sensalari C, Maere S, Lohaus R. 2021. ksrates: positioning whole-genome duplications relative to speciation events in KS distributions. *Bioinformatics* doi:10.1093/bioinformatics/btab602.

Simillion C, Janssens K, Sterck L, Van de Peer Y. 2008. i-ADHoRe 2.0: an improved tool to detect degenerated genomic homology using genomic profiles. *Bioinformatics* **24**: 127-128.

Smith DR, Crosby K, Lee RW. 2011. Correlation between nuclear plastid DNA abundance and plastid number supports the limited transfer window hypothesis. *Genome Biol Evol* **3**: 365-371.

Smith H. 2000. Phytochromes and light signal perception by plants-an emerging synthesis. *Nature* **407**: 585-591.

Smith SA, Beaulieu JM, Donoghue MJ. 2010. An uncorrelated relaxed-clock analysis suggests an earlier origin for flowering plants. *Proceedings of the National Academy of Sciences* **107**: 5897-5902.

Steuernagel B, Witek K, Krattinger SG, Ramirez-Gonzalez RH, Schoonbeek HJ, Yu G, Baggs E, Witek AI, Yadav I, Krasileva KV et al. 2020. The NLR-Annotator Tool Enables Annotation of the Intracellular Immune Receptor Repertoire. *Plant Physiol* **183**: 468-482.

Szöllõsi GJ, Rosikiewicz W, Boussau B, Tannier E, Daubin V. 2013. Efficient exploration of the space of reconciled gene trees. *Syst Biol* **62**: 901-912.

Tang H, Bowers JE, Wang X, Ming R, Alam M, Paterson AH. 2008. Synteny and collinearity in plant genomes. *Science* **320**: 486-488.

Tang H, Bowers JE, Wang X, Paterson AH. 2010. Angiosperm genome comparisons reveal early polyploidy in the monocot lineage. *Proc Natl Acad Sci U S A* **107**: 472-477.

Tillich M, Lehwark P, Pellizzer T, Ulbricht-Jones ES, Fischer A, Bock R, Greiner S. 2017. GeSeq – versatile and accurate annotation of organelle genomes. *Nucleic Acids Research* **45**: W6-W11.

Toda N, Rustenholz C, Baud A, Le Paslier MC, Amselem J, Merdinoglu D, Faivre-Rampant P. 2020. NLGenomeSweeper: A Tool for Genome-Wide NBS-LRR Resistance Gene Identification. *Genes (Basel)* **11**.

Touchette BW, Burkholder J. 2001. Nitrate reductase activity in a submersed marine angiosperm: Controlling influences of environmental and physiological factors. *Plant Physiology and Biochemistry* **39**: 583-593.

Touchette BW, Burkholder JM. 2007. Carbon and nitrogen metabolism in the seagrass, Zostera marina L.: Environmental control of enzymes involved in carbon allocation and nitrogen assimilation. *Journal of Experimental Marine Biology and Ecology* **350**: 216-233.

Tuskan GA Difazio S Jansson S Bohlmann J Grigoriev I Hellsten U Putnam N Ralph S Rombauts S Salamov A et al. 2006. The genome of black cottonwood, Populus trichocarpa (Torr. & Gray). *Science* **313**: 1596-1604.

Vanneste K, Baele G, Maere S, Van de Peer Y. 2014. Analysis of 41 plant genomes supports a wave of successful genome duplications in association with the Cretaceous-Paleogene boundary. *Genome Res* **24**: 1334-1347.

von Koskull-Döring P, Scharf KD, Nover L. 2007. The diversity of plant heat stress transcription factors. *Trends Plant Sci* **12**: 452-457.

Walker BJ, Abeel T, Shea T, Priest M, Abouelliel A, Sakthikumar S, Cuomo CA, Zeng Q, Wortman J, Young SK et al. 2014. Pilon: an integrated tool for comprehensive microbial variant detection and genome assembly improvement. *PLoS One* **9**: e112963.

Wang H, Tang X, Chen J, Shang S, Zhu M, Liang S, Zang Y. 2021. Comparative studies on the response of Zostera marina leaves and roots to ammonium stress and effects on nitrogen metabolism. *Aquatic Toxicology* **240**.

Wang J, Du Z, Huo X, Zhou J, Chen Y, Zhang J, Pan A, Wang X, Wang F, Zhang J. 2020. Genome-wide analysis of PRR gene family uncovers their roles in circadian rhythmic changes and response to drought stress in Gossypium hirsutum L. *PeerJ* **8**: e9936-e9936.

Wang W, Haberer G, Gundlach H, Glasser C, Nussbaumer T, Luo MC, Lomsadze A, Borodovsky M, Kerstetter RA, Shanklin J et al. 2014. The Spirodela polyrhiza genome reveals insights into its neotenous reduction fast growth and aquatic lifestyle. *Nat Commun* **5**: 3311.

Wang X, Shi X, Chen S, Ma C, Xu S. 2018. Evolutionary Origin, Gradual Accumulation and Functional Divergence of Heat Shock Factor Gene Family with Plant Evolution. *Front Plant Sci* **9**: 71.

Xin Z, Mandaokar A, Chen J, Last RL, Browse J. 2007. Arabidopsis ESK1 encodes a novel regulator of freezing tolerance. *Plant J* **49**: 786-799.

Xu S, Xu S, Zhou Y, Yue S, Qiao Y, Liu M, Gu R, Song X, Zhang Y, Zhang X. 2020. Sonar and in situ surveys of eelgrass distribution, reproductive effort, and sexual recruitment contribution in a eutrophic bay with intensive human activities: Implication for seagrass conservation. *Mar Pollut Bull* **161**: 111706.

Xue GP, Sadat S, Drenth J, McIntyre CL. 2014. The heat shock factor family from Triticum aestivum in response to heat and other major abiotic stresses and their role in regulation of heat shock protein genes. *J Exp Bot* **65**: 539-557.

Yang Z. 2007a. PAML 4: phylogenetic analysis by maximum likelihood. *Mol Biol Evol* **24**: 1586-1591.

Yang ZH. 2007b. PAML 4: Phylogenetic analysis by maximum likelihood. *Molecular Biology and Evolution* **24**: 1586-1591.

Yin R, Arongaus AB, Binkert M, Ulm R. 2015. Two distinct domains of the UVR8 photoreceptor interact with COP1 to initiate UV-B signaling in arabidopsis. *Plant Cell* **27**: 202-213.

Zarnoch CB, Hoellein TJ, Furman BT, Peterson BJ. 2017. Eelgrass meadows, Zostera marina (L.), facilitate the ecosystem service of nitrogen removal during simulated nutrient pulses in Shinnecock Bay, New York, USA. *Mar Pollut Bull* **124**: 376-387.

Zhang GJ, Dong R, Lan LN, Li SF, Gao WJ, Niu HX. 2020. Nuclear Integrants of Organellar DNA Contribute to Genome Structure and Evolution in Plants. *Int J Mol Sci* **21**.

Zhao N, Grover CE, Chen Z, Wendel JF, Hua J. 2019. Intergenomic gene transfer in diploid and allopolyploid Gossypium. *BMC Plant Biol* **19**: 492.

Zheng S, Poczai P, Hyvonen J, Tang J, Amiryousefi A. 2020. Chloroplot: An Online Program for the Versatile Plotting of Organelle Genomes. *Front Genet* **11**: 576124.

Zhong X. 2020. Assembly, annotation and analysis of chloroplast genomes. doi:10.26182/5f333d9ac2bee.

Zhuang L, Cao W, Wang J, Yu J, Yang Z, Huang B. 2018. Characterization and Functional Analysis of FaHsfC1b from Festuca arundinacea Conferring Heat Tolerance in Arabidopsis. *Int J Mol Sci* **19**.

Zwaenepoel A, Van de Peer Y. 2019a. Inference of Ancient Whole-Genome Duplications and the Evolution of Gene Duplication and Loss Rates. *Mol Biol Evol* **36**: 1384-1404.

Zwaenepoel A, Van de Peer Y. 2019b. wgd-simple command line tools for the analysis of ancient whole-genome duplications. *Bioinformatics* **35**: 2153-2155.
